# WHO systematic review of prevalence of chronic pelvic pain: a neglected reproductive health morbidity

**DOI:** 10.1186/1471-2458-6-177

**Published:** 2006-07-06

**Authors:** Pallavi Latthe, Manish Latthe, Lale Say, Metin Gülmezoglu, Khalid S Khan

**Affiliations:** 1 Birmingham Women's Healthcare NHS Trust, Birmingham, UK; 2 Tower Hill Medical Centre, Great Barr, Birmingham; 3 UNDP/UNFPA/WHO/World Bank Special Programme of Research, Development and Research Training in Human Reproduction, Department of Reproductive Health and Research, World Health Organization, Geneva, Switzerland; 4 Academic Department of Obstetrics & Gynaecology, University of Birmingham, Birmingham, UK

## Abstract

**Background:**

Health care planning for chronic pelvic pain (CPP), an important cause of morbidity amongst women is hampered due to lack of clear collated summaries of its basic epidemiological data. We systematically reviewed worldwide literature on the prevalence of different types of CPP to assess the geographical distribution of data, and to explore sources of variation in its estimates.

**Methods:**

We identified data available from Medline (1966 to 2004), Embase (1980 to 2004), PsycINFO (1887 to 2003), LILACS (1982 to 2004), Science Citation index, CINAHL (January 1980 to 2004) and hand searching of reference lists. Two reviewers extracted data independently, using a piloted form, on participants' characteristics, study quality and rates of CPP. We considered a study to be of high quality (valid) if had at least three of the following features: prospective design, validated measurement tool, adequate sampling method, sample size estimation and response rate >80%. We performed both univariate and multivariate meta-regression analysis to explore heterogeneity of results across studies.

**Results:**

There were 178 studies (459975 participants) in 148 articles. Of these, 106 studies were (124259 participants) on dysmenorrhoea, 54 (35973 participants) on dyspareunia and 18 (301756 participants) on noncyclical pain. There were only 19/95 (20%) less developed and 1/45 (2.2%) least developed countries with relevant data in contrast to 22/43 (51.2%) developed countries. Meta-regression analysis showed that rates of pain varied according to study quality features. There were 40 (22.5%) high quality studies with representative samples. Amongst them, the rate of dysmenorrhoea was 16.8 to 81%, that of dyspareunia was 8 to 21.8%, and that for noncyclical pain was 2.1 to 24%.

**Conclusion:**

There were few valid population based estimates of disease burden due to CPP from less developed countries. The variation in rates of CPP worldwide was due to variable study quality. Where valid data were available, a high disease burden of all types of pelvic pain was found.

## Background

Chronic pelvic pain (CPP) is a debilitating condition among women with a major impact on health-related quality of life, work productivity and health care utilisation. It is the single most common indication for referral to women's health services accounting for 20% of all outpatient appointments in secondary care [1]. This leads to a substantial burden on limited health care resources. For example, $881.5 million are spent per year on its outpatient management in the USA [2] and an estimated £158 million are spent annually on the management of this condition in the UK National Health Service [3].

Valid information about the true extent of CPP is an essential consideration in resource allocation and health care planning. In addition, these basic epidemiological data are necessary to monitor trends of the disease burden as well as to inform design of other research in this condition, like genetic and environmental epidemiology to assess aetiology, qualitative studies to establish well being and overall quality of life, and studies aimed at the development of new treatment strategies [4]. The epidemiological features of CPP have been generously reported in the worldwide literature. The majority of the studies are limited by small sample size and hence their estimates are imprecise. The need to summarise these data have generally received scant attention [5].

A systematic literature review was performed to ascertain: the prevalence rates of CPP according to the type of pain; its geographical distribution; its variation within subgroups defined by age and development status of the country of origin; and the effect of study quality and representativeness on the rates.

## Methods

Our systematic review followed a protocol developed using widely recommended methodology [6,7] and complied with the MOOSE statement [8].

### Data sources

We searched general bibliographic databases: Medline (1966–2004), Embase (1980–2004) and PSYCHINFO (1887–2004). We also searched specialist computer databases: LILACS (Literatura Latinoamericana y del Caribe en Ciencias de la Salud 1982 to 2004), CINAHL (January 1980 to 2004) and SCISEARCH (1974–2004). Our search term combination for electronic databases was as follows: MeSH headings, text words and word variants for "pelvic pain" or "dysmenorrhoea" or "dyspareunia" or "low abdominal pain" were combined using Boolean operator and with terms like "prevalence" or "community survey" or "incidence". These were combined with terms representing relevant study designs e.g. cross-section, survey etc. according to recent recommendations [9] for searching and the search was restricted to human and female. We also hand searched the bibliographies of all relevant reviews and primary studies to identify cited articles not captured by electronic searches. The search did not have any language restrictions. We also attempted to contact authors from indexed electronic abstracts on theses for data.

### Study selection

Studies on CPP were selected using the following predefined criteria:

#### Participants

Non-pregnant women without cancer participating in surveys about rates of CPP. Women with or without known endometriosis or irritable bowel syndrome were to be included

#### Outcome

There is lack of consensus on the definition of CPP in the published literature [10]. We used a definition based on duration and nature of pain (constant or intermittent, cyclical or noncyclical pain, that persisted for 3 months or more [11]) and included three types: cyclical pain during menstruation (dysmenorrhoea), deep dyspareunia and noncyclical pelvic pain. Studies were included in the absence of information on duration of pain as long as it was explicit that cases of acute pain were excluded.

There is a debate about the definition of CPP as recurrent pain such as that associated with isolated dysmenorrhoea and dyspareunia is often considered biologically distinct from chronic pain, however many women have overlapping symptoms. For our review, CPP may be regarded as a composite of chronic and recurrent pelvic pain.

#### Study design

Cross sectional studies that reported the prevalence of CPP.

### Data extraction and quality assessment

Two reviewers (PML, ML) extracted data independently, using a piloted form, on participants' characteristics, study quality and rates of CPP. Data on studies not published in English were extracted by people with a medical background with command of the relevant language. In some studies the existence of multiple symptoms amongst individuals could not be evaluated separately due to the structure of their questionnaires and their manner of reporting, so these were excluded. Two of the datasets in the systematic review of dysmenorrhoea prevalence were extracted from the abstracts of theses were when the full copies could not be obtained [12,13].

The methodological quality of all selected papers was assessed to evaluate internal validity using the following attributes [6]: (a) Study design to determine if CPP assessment had been performed prospectively to minimise recall bias; (b) Adequacy of sampling by assessing whether recruitment of participants was random or consecutive or a convenience sample; (c) Sufficiently high response rate (>80%); (d) Use of a validated measurement tool to ascertain CPP [14] as this ensures that participants' responses are a true representation of the underlying condition; (e) Sample size calculation so as to ascertain prevalence reliably. It was considered 'adequate' if the studies had mentioned that sample size was calculated and 'inadequate' if this was not done or not mentioned explicitly. The studies were classified into high and low quality groups based on compliance with 3/5 quality criteria or more. Representativeness of the sample for general population (source of sample) was considered separately to methodological quality as this relates to external validity. This distinction is important because internally valid studies of women attending hospitals or for private health care checks may not be biased but they are less useful due to sampling of non-generalisable population groups.

Numerators and denominators were extracted or estimated from each study for computing rates and confidence intervals (CI). In most studies, prevalence measured how many women had CPP at a single point in time, i.e. point prevalence [14]. Period prevalence, based on the number of women developing CPP during a defined period of time, was reported only in a few studies.

### Data synthesis

For each study, we computed prevalence rates and their 95% CI according to the three different types of CPP. Rates of the different CPP were mapped to depict the variation in prevalence by country of origin. Heterogeneity was explored in the log rates of CPP graphically using forest plots of point estimates of rates and their 95% CI and statistically using Cochrane Q and I^2^, a statistic that quantifies the degree of inconsistency across studies in a meta-analysis on a scale ranging from 0–100% [15,16]. In reviews with high degree of inconsistency meta-analysis is not recommended. Meta-regression explored if inconsistency in results across individual studies could be explained by variations in countries' development status, participants' age, representativeness of the sample and methodological quality of the included studies [17]. For development status we used the United Nations classification (developed, less developed and least developed) for countries. Study quality was assessed separately for individual items and scores. We performed both univariate and multivariate meta-regression analysis. We did not pool results even from high quality representative studies due to the statistically significant heterogeneity in this subgroup of high quality representative studies. We however, have reported the rate ranges for representative studies [18]. Publication and related biases were examined for by plotting log rates versus their corresponding variances in a funnel plot. Funnel asymmetry was tested by Begg's test [19].

## Results

**Figure 1:**
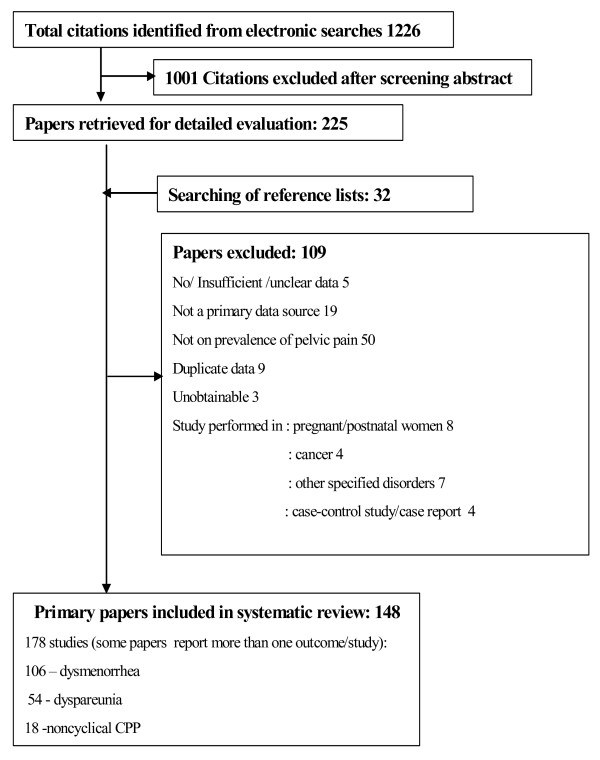
Study selection for systematic review on prevalence of chronic pelvic pain.

**Figure 2:**
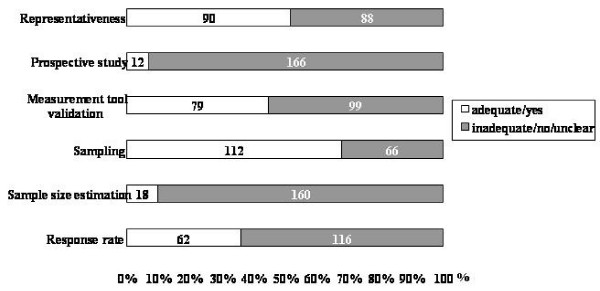
**Quality of studies included in systematic review on prevalence of chronic pelvic pain. **(Data presented as 100% stacked bars; figures in the stacks represent number of studies).

The electronic search yielded a total of 1226 citations (figure 1). On examination of titles and abstracts, 225 were found to be potentially relevant and their full papers were obtained. The reference lists of these revealed 32 further citations. After reviewing these, 109 papers were excluded (see additional file 3). The remaining 148 papers (see additional file 2 for a complete list of included articles) met the inclusion criteria, which provided data on 459972 participants. 30 studies overlapped and reported more than one outcome or more than one group of population. There is very little data (1/148 papers) available from the least developed countries. 22/43 developed countries had published data on prevalence of CPP in contrast to 19/95 less developed and 1/46 least developed countries (P < 0.0001) [the denominator is the number of developed, less developed and least developed countries in the world according to UN country classification]. Study quality assessment (figure 2) revealed deficiencies in many areas of methodology: Two (1.2%) studies met all five high quality criteria, 13 (7.1%) met 4/5 criteria and 40 (22.5%) met three or more criteria.

**Figure 3:**
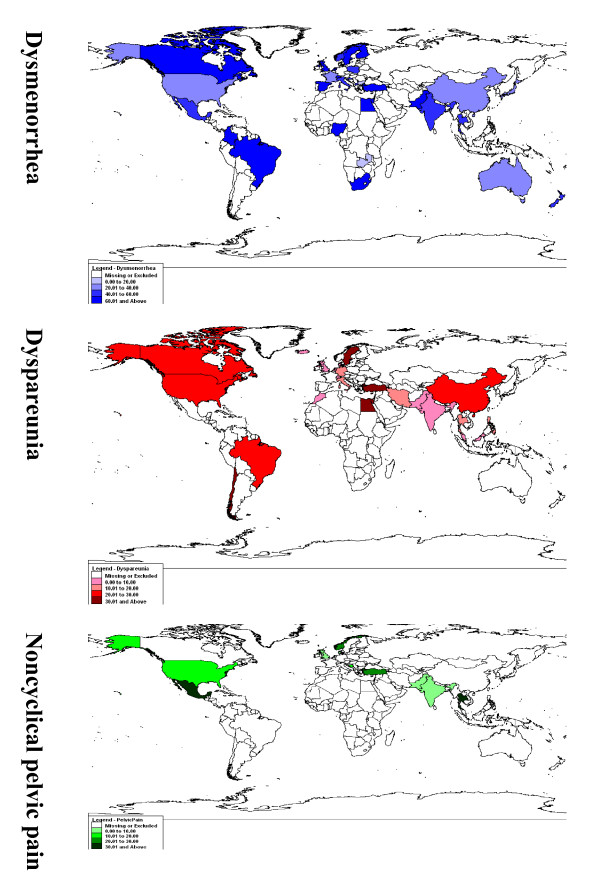
Epimaps of worldwide prevalence of chronic pelvic pain.

**Figure 4:**
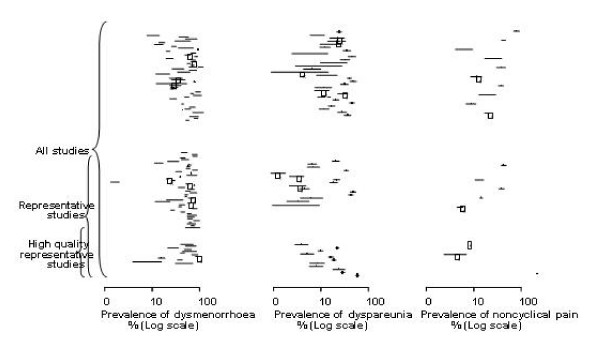
Prevalence of different types of chronic pelvic pain.

The details of studies included in the systematic review on prevalence of dysmenorrhoea, dyspareunia and noncyclical pelvic pain are given in table 1, 2 and 3 respectively (additional file 1). The data on prevalence of CPP in included studies is summarised in figures 3, 4. Epimaps in Figure 3 depict the available data by countries, on worldwide prevalence of different types of chronic pelvic pain by percentage.

### Dysmenorrhoea

**Figure 5:**
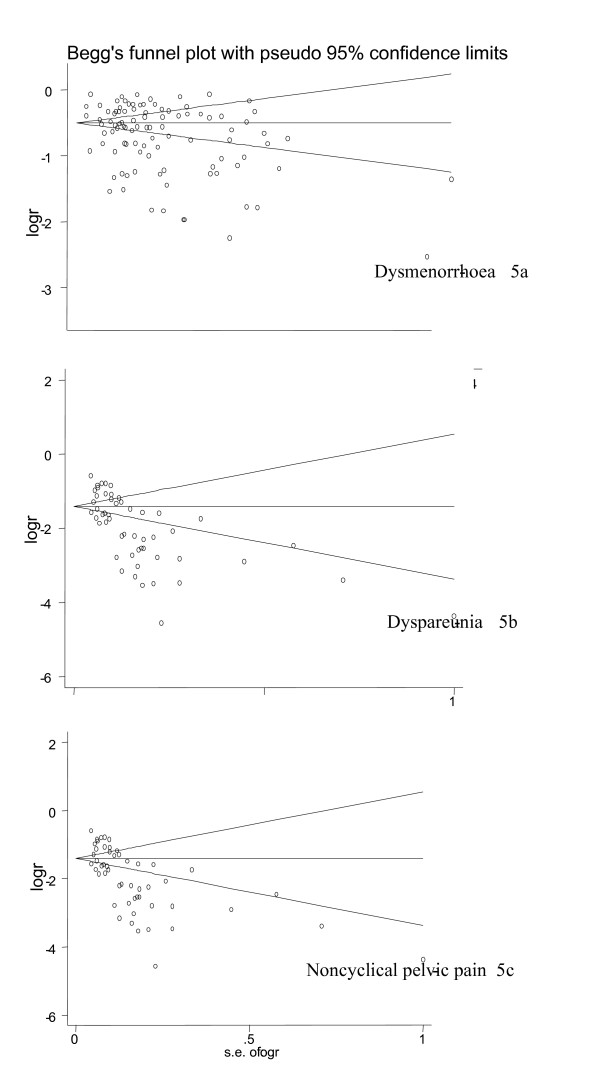
**Funnel plots of the three types of pelvic pain prevalence studies. **[a – dysmenorrhoea; b – dyspareunia; c – noncyclical pelvic pain]

The prevalence rates ranged from 1.7% [2] to 97% [20] in 106 studies including 125249 women. Prevalence rates for cyclical pelvic pain in the UK reported were between 45% (12% reporting severe dysmenorrhoea) [21] to 97% [20] (14% severe) for any dysmenorrhoea in community based studies and between 41–62% in hospital based studies [22-24]. In other European countries it was similar [25,26]. The lowest prevalence was reported in Bulgaria (8.8%) in women hospitalised with adnexitis between the ages of 19–41 years and the highest was in Finland (94%) in girls aged 10–20 years [27]. In 20 high quality studies with representative samples, the rate of dysmenorrhoea was reported between 16.8 [28] to 81% [29]. There was substantial heterogeneity in forest plots and I^2 ^statistic was 98% (figure 4). The funnel plot for dysmenorrhoea was asymmetrical (Begg's test P = 0.02; figure 5a) but not for representative studies (P = 0.333). Metaregression (Table 4) showed validated measurement tool to be a significant factor to explain heterogeneity but not study quality score, representativeness, age < 25 years or development status of the country (developed versus less developed versus least developed).

### Dyspareunia

The prevalence rates ranged from 1.3% [30] to 45.7% [31] in 54 studies including 35973 women. The rates of dyspareunia varied from 1.1% in Sweden [32] to 45% [31] in US studies. In 18 high quality studies with representative samples, the rate range of dyspareunia was reported to be 8 [33] to 21.1 [34]% Studies were markedly heterogeneous (I^2 ^= 97.9%) and the funnel plot for dyspareunia was asymmetrical (Begg's test P = 0.001; figure 5b) but not in representative studies (P = 0.227). The representativeness of sample provided the main explanation for heterogeneity that was statistically significant in meta-regression analysis (table 4) but age under 60 was not (P = 0.15).

### Non-cyclical pelvic pain

The prevalence rates ranged from 4.0% [35] to 43.4% [36] in 18 studies including 299740 women. Two recent studies stated a 3 month prevalence of 15% in women aged 18–50 years in the USA [2] and 24% in ages between 12–70 in the UK [37]. In less developed countries in South East Asia the prevalence rates varied from 5.2% in India, 8.8% in Pakistan to 43.2% in Thailand [36]. Amongst the 3 high quality studies with representative samples, the rate range of noncyclical pain was 2.1 [37] to 24 [29]% as reported from the primary studies. There was heterogeneity across studies (I^2 ^= 99%). Metaregression (table 4) revealed that prospective design, adequate sampling strategy and sample size estimation tended to describe lower prevalence of noncyclical pelvic pain though none of these were significant (table 4). The funnel plot for noncyclical pelvic pain was asymmetrical (Begg's test P = 0.048; figure 5c) but not for representative studies (Begg's test P = 0.88).

## Discussion

This is the first systematic review of the worldwide prevalence of CPP. The variation in rates of CPP worldwide was explained by variable study quality. The number of population-based studies yielding estimates of burden of CPP from less developed countries was low. High quality literature comprising representative samples revealed a high burden of disease for all types of pelvic pain, however, there remained heterogeneity in these subgroup of studies.

We believe that the findings of our study are valid as our review methodology was rigorous. A prospective review protocol was used and a concerted effort made to identify all the available evidence without language restriction. We made concerted efforts to report this systematic review as suggested by the MOOSE consensus statement [8]. Both the methodology and the rates of CPP varied among the included primary studies and we explored the reasons for variations comprehensively. This review represents the best available evidence on the estimates of the prevalence of CPP at the time of writing and rate ranges from high quality studies of representative samples provide the best information available for targeting services at women suffering from pelvic pain.

The variation in geographical distribution may be related to study characteristics, study quality, age groups included and definitions used rather than intrinsic differences between the prevalence of CPP between the different populations. Other plausible explanations might be differences in the prevalence of sexually transmitted infections, availability of medical and other resources or cultural differences. Although we have included studies from 1924 onwards, majority of the studies are from 1980 onwards. The population demographics are unlikely to have undergone major changes over this period, making the studies relevant to current populations' Nevertheless, one has to bear in mind the changes in patterns of smoking, sexually transmitted infections and contraception during this time and their consequences for pelvic pain [38]. Substantial differences in and even complete absence of definitions, together with differences in age ranges of the populations studied also complicate the interpretation of our findings.

One study noted the overlaps among different pain disorders (irritable bowel syndrome, interstitial cystitis etc) [39], A better understanding of the epidemiology of these disorders would facilitate our understanding of somato-sensory disorders in general. We explored for heterogeneity using sophisticated technique of metaregression. We found that validated measurement tool, representativeness and high quality were the variables that were statistically significant (P < 0.05) for dysmenorrhoea, dyspareunia and noncyclical pelvic pain respectively. In the meta-regression for countries classification, there are so few least developed countries that the power is very low. Also, it is worth pointing out that there is a potential risk of aggregation bias when age is used as a variable in meta-regression.

The information on the rates of dysmenorrhoea, dyspareunia have implication for provision of services to policymakers in terms of provision of improved access for these women to health care resources as well as the development of appropriate treatment protocols. Future epidemiological studies should ideally be prospective, with explicit definitions of the outcome and representative of the general population. Close attention must be paid to study design and to the use the validated measurement tools for validity and comparability of the results across studies and regions. Such efforts will need funding agencies to be willing to support broader and more systems oriented approach [40].

## Conclusion

There were few valid population based estimates of disease burden due to CPP from less developed countries. The variation in rates of CPP worldwide was due to variable study quality. Where valid data were available, a high disease burden of all types of pelvic pain was found.

## Funding source

World Health Organization

## Competing interests

The author(s) declare that they have no competing interests.

## Authors' contributions

Dr. P Latthe: Conception, design, search, study selection, data extraction, data synthesis, writing the manuscript

Dr. M Latthe: Data extraction, tables

Dr L Say, Dr AM Gülmezoglu: Revising the manuscript, figures

Prof. KS Khan: Conception, design, data synthesis, writing and revising the manuscript

## Pre-publication history

The pre-publication history for this paper can be accessed here:



## Supplementary Material

Additional file 2 List of studies included in the systematic review of prevalence of chronic pelvic pain Click here for file

Additional file 3 Appendix of excluded studies Click here for file

Additional file 1 Tables 1, 2 and 3 of tables of characterstics of included studies on dysmenorrhoea, dyspareunia and noncyclical pelvic pain respectively Click here for file

## References

[B1] Howard FM (1993). The role of laparoscopy in chronic pelvic pain: promise and pitfalls. Obstet Gynecol Surv.

[B2] Mathias SD, Kuppermann M, Liberman RF, Lipschutz RC, Steege JF (1996). Chronic pelvic pain: prevalence, health-related quality of life, and economic correlates. Obstet Gynecol.

[B3] Davies L, Ganger K, Drummond M, Saunders D, Beard R (1992). The economic burden of intractable gynaecological pain. J Obstet Gynecol.

[B4] Zondervan K, Barlow DH (2000). Epidemiology of chronic pelvic pain. Best Pract Res Clin Obstet Gynaecol.

[B5] Dickersin K (2002). Systematic reviews in epidemiology: why are we so far behind?. Int J Epidemiol.

[B6] Glasziou P, Irwig L, Bain C, Colditz G (2001). Frequency and Rate. Systematic Reviews in Health Care: A practical guide.

[B7] Khan KS, ter Riet G, Popay J, J K, Khan KS, ter Riet G, Popay J, Nixon J and J K, Nixon J, (eds.) (2001). Undertaking systematic reviews of research on effectiveness (CRD Report No 4).

[B8] Stroup DF, Berlin JA, Morton SC, Olkin I, Williamson GD, Rennie D, Moher D, Becker BJ, Sipe TA, Thacker SB (2000). Meta-analysis of observational studies in epidemiology: a proposal for reporting. Meta-analysis Of Observational Studies in Epidemiology (MOOSE) group. JAMA.

[B9] Philips Z, Sculpher M, Claxton K, Golder S, Ginelly, RiemsmaR (2004). Review of guidelines for good practice in decision analytic modelling in health technology assessment. Health Technology Assessment.

[B10] Williams RE, Hartmann KE, Steege JF (2004). Documenting the current definitions of chronic pelvic pain: implications for research. Obstet Gynecol.

[B11] Vercellini P, Fedele L, Arcaini L, Bianchi S, Rognoni MT, Candiani GB (1989). Laparoscopy in the diagnosis of chronic pelvic pain in adolescent women. J Reprod Med.

[B12] Céspedes Maturana L, Cornejo Araya P (1997). Prevalencia de síntomas premenstruales y dismenorrea en mujeres de edad fértil y su relación con el ausentismo laboral / Prevalence of premenstrual symptoms and dysmenorrhea in plentiful women and their relationship with work absence.

[B13] González Bahamonde M, Ibarra Farías M (1999). Conocimientos y prácticas de autocuidado sobre síndrome premenstrual y dismenorrea de un grupo de alumnas de la Facultad de Educación de la Pontificia Universidad Católica de Chile / Selfcare knowledge and practice about premenstrual syndrome and dysmenorrea in a group of female students from Facultad de Educación, Pontificia Universidad Católica de Chile..

[B14] Streiner DL, Norman GR (1998). Measurement. PDQ Epidemiology.

[B15] Higgins JP, Thompson SG (2002). Quantifying heterogeneity in a meta-analysis. Stat Med.

[B16] Higgins JP, Thompson SG, Deeks JJ, Altman DG (2003). Measuring inconsistency in meta-analyses. BMJ.

[B17] Song F, Sheldon TA, Sutton AJ, Abrams KR, Jones DR (2001). Methods for exploring heterogeneity in meta-analysis. Eval Health Prof.

[B18] Khan KS, Wojdyla D, Say L, Gulmezoglu AM, Van Look PF (2006). WHO analysis of causes of maternal death: a systematic review. Lancet.

[B19] Begg CB, Mazumdar M (1994). Operating characteristics of a rank correlation test for publication bias. Biometrics.

[B20] Gath D, Osborn M, Bungay G, Iles S, Day A, Bond A, Passingham C (1987). Psychiatric disorder and gynaecological symptoms in middle aged women: a community survey. BMJ (Clin Res Ed).

[B21] Kessel N, Coppen A (1963). The prevalence of common menstrual symptoms. Lancet.

[B22] Mahmood TA, Templeton AA, Thomson L, Fraser C (1991). Menstrual symptoms in women with pelvic endometriosis. BJOG.

[B23] Liu DT, Hitchcock A (1986). Endometriosis: its association with retrograde menstruation, dysmenorrhoea and tubal pathology. BJOG.

[B24] Andersch B, Milsom I (1982). An epidemiologic study of young women with dysmenorrhea. Am J Obstet Gynecol.

[B25] Sundell G, Milsom I, Andersch B (1990). Factors influencing the prevalence and severity of dysmenorrhoea in young women. BJOG.

[B26] Bergsjo P, Jenssen H, Vellar OD (1975). Dysmenorrhea in industrial workers. Acta Obstet Gynecol Scand.

[B27] Widholm O, Kantero RL (1971). A  statistical analysis of the menstrual patterns of 8000 Finnish girls and their mothers. Acta Obstet Gynecol Scand.

[B28] Woods NF, Most A, Dery GK (1982). Prevalence of perimenstrual symptoms. Am J Public Health.

[B29] Zondervan KT, Yudkin PL, Vessey MP, Jenkinson CP, Dawes MG, Barlow DH, Kennedy SH (2001). The community prevalence of chronic pelvic pain in women and associated illness behaviour. Br J Gen Pract.

[B30] Garde K, Lunde I (1980). Female sexual behaviour. A study in a random sample of 40-year-old women. Maturitas.

[B31] Jamieson DJ, Steege JF (1996). The prevalence of dysmenorrhea, dyspareunia, pelvic pain, and irritable bowel syndrome in primary care practices. Obstet Gynecol.

[B32] Oberg K, Fugl-Meyer AR, Fugl-Meyer KS (2004). On categorization and quantification of women's sexual dysfunctions: an epidemiological approach. Int J Impot Res.

[B33] Osborn M, Hawton K, Gath D (1988). Sexual dysfunction among middle aged women in the community. BMJ.

[B34] Cain VS, Johannes CB, Avis NE, Mohr B, Schocken M, Skurnick J, Ory M (2003). Sexual functioning and practices in a multi-ethnic study of midlife women: Baseline results from SWAN. Journal of Sex Research.

[B35] Rulin MC, Davidson AR, Philliber SG, Graves WL, Cushman LF (1993). Long-term effect of tubal sterilization on menstrual indices and pelvic pain. Obstet Gynecol.

[B36] Thongkrajai P, Pengsaa P, Lulitanond V (1999). An epidemiological survey of female reproductive health status: gynecological complaints and sexually-transmitted diseases. Southeast Asian J Trop Med Public Health.

[B37] Zondervan KT, Yudkin PL, Vessey MP, Dawes MG, Barlow DH, Kennedy SH (1999). Prevalence and incidence of chronic pelvic pain in primary care: Evidence from a national general practice database. Br J Obstet Gynaecol.

[B38] Latthe P, Mignini L, Gray R, Hills R, Khan K (2006). Factors predisposing women to chronic pelvic pain: systematic review. BMJ.

[B39] Zondervan KT, Yudkin PL, Vessey MP, Jenkinson CP, Dawes MG, Barlow DH, Kennedy SH (2001). Chronic pelvic pain in the community - Symptoms, investigations, and diagnoses. Am J Obstet Gynecol.

[B40] Rudan I, Lawn J, Cousens S, Rowe AK, Boschi-Pinto C, Tomaskovic L, Mendoza W, Lanata CF, Roca-Feltrer A, Carneiro I, Schellenberg JA, Polasek O, Weber M, Bryce J, Morris SS, Black RE, Campbell H (2005). Gaps in policy-relevant information on burden of disease in children: a systematic review. Lancet.

