# Supplementary material for: WHO systematic review of prevalence of chronic pelvic pain: a neglected reproductive health morbidity
Source: BMC Public Health. 2006 Jul 6;6:177. doi: 10.1186/1471-2458-6-177 (PMC1550236; doi:10.1186/1471-2458-6-177)
Supplement: Additional file 2 — List of studies included in the systematic review of prevalence of chronic pelvic pain [file 1471-2458-6-177-S2.doc]

# List of included studies

Prevalence of dysmenorrhoea^1-103^

Prevalence of dyspareunia^18;19;38;44;55;89-92;95;96;104-143^

Prevalence of noncyclical pelvic pain^18;25;44;64;90;91;94-96;109;114;141;144-148^

Reference List

1. Pawlowski B. Prevalence of menstrual pain in relation to the reproductive life history of women from the Mayan rural community. *Ann.Hum.Biol* 2004;**31**:1-8.

2. Weissman AM, Hartz AJ, Hansen MD, Johnson SR. The natural history of primary dysmenorrhoea: a longitudinal study. *BJOG* 2004;**111**:345-52.

3. Barnard K, Frayne SM, Skinner KM, Sullivan LM. Health status among women with menstrual symptoms. *J Womens Health (Larchmt.)* 2003;**12**:911-9.

4. Schmidt E,.Herter LD. Dismenorréia em adolescentes escolares / Dysmenorrhea in school adolescents . Adolesc.latinoam 2002;3:ISSN 1414-7130.

5. Walraven G, Ekpo G, Coleman R, Scherf C, Morison L, Harlow SD. Menstrual disorders in rural Gambia. *Stud Fam Plan* 2002;**33**:261-8.

6. Larroy C, Crespo M, Meseguer C. Functional dysmenorrhea in the Autonomous Community of Madrid: Study of prevalence according to age. *Revista de la Sociedad Espanola del Dolor* 2001;**8**:11-22.

7. Zondervan KT, Yudkin PL, Vessey MP, Jenkinson CP, Dawes MG, Barlow DH *et al*. The community prevalence of chronic pelvic pain in women and associated illness behaviour. *Br J Gen Pract* 2001;**51**:541-7.

8. Balbi C, Musone R, Menditto A, Di Prisco L, Cassese E, D'Ajello M *et al*. Influence of menstrual factors and dietary habits on menstrual pain in adolescence age. *Eur J Obstet Gynecol Reprod Biol* 2000;**91**:143-8.

9. Dusek T. Influence of high intensity training on menstrual cycle disorders in athletes. *Croatian Medical Journal* 2001;**42**:79-82.

10. Banikarim C, Chacko MR, Kelder SH. Prevalence and impact of dysmenorrhea on hispanic female adolescents. *Arch Pediatr Adolesc Med* 2000;**154**:1226-9.

11. Gordley LB, Lemasters G, Simpson SR, Yiin JH. Menstrual disorders and occupational, stress, and racial factors among military personnel. *Journal of Occupational & Environmental Medicine* 2000;**42**:871-81.

12. Mishra GD, Dobson AJ, Schofield MJ. Cigarette smoking, menstrual symptoms and miscarriage among young women. *Australian & New Zealand Journal of Public Health* 2000;**24**:413-20.

13. Wu D, Wang X, Chen D, Niu T, Ni J, Liu X *et al*. Metabolic gene polymorphisms and risk of dysmenorrhea. *Epidemiology* 2000;**11**:648-53.

14. Visvanathan N,.Wyshak G. Tubal ligation, menstrual changes, and menopausal symptoms. *J Womens Health Gend Based Med* 2000;**9**:521-7.

15. Hillen TI, Grbavac SL, Johnston PJ, Straton JA, Keogh JM. Primary dysmenorrhea in young Western Australian women: prevalence, impact, and knowledge of treatment. *Journal of Adolescent Health* 1999;**25**:40-5.

16. Kritz SD, Wingard DL, Garland FC. The association of behavior and lifestyle factors with menstrual symptoms. *Journal of Women's Health & Gender Based Medicine* 1999;**8**:1185-93.

17. Sultan C, Parisi F, Feki M, Rasandratana A, Attal G, Legasal P *et al*. Epidemiology of dysmenorrhea in adolescents in France. *Annales de Pediatrie* 1999;**46**:518-25.

18. Thongkrajai P, Pengsaa P, Lulitanond V. An epidemiological survey of female reproductive health status: gynecological complaints and sexually-transmitted diseases. *Southeast Asian J Trop Med Public Health* 1999;**30**:287-95.

19. Golding JM, Wilsnack SC, Learman LA. Prevalence of sexual assault history among women with common gynecologic symptoms. *Am J Obstet Gynecol* 1998;**179**:1013-9.

20. Pedron-Nuevo N, Gonzalez-Unzaga LNM, Celis-Carrillo R, Reynoso-Isla M, Torre-Romeral L. Frequency of dysmenorrhea and associated symptoms in women of 12 to 24 years. *Ginecologia y Obstetricia de Mexico, Vol 66(DEC* 1998;**)**.

21. Campbell MA,.McGrath PJ. Use of medication by adolescents for the management of menstrual discomfort. *Arch.Pediatr.Adolesc.Med* 1997;**151**:905-13.

22. Elahi N,.Parveen N. Menstrual disorders in adolescent age group. *Journal of the College of Physicians and Surgeons Pakistan* 1997;**7**:105-7.

23. Harlow SD,.Park M. A longitudinal study of risk factors for the occurrence, duration and severity of menstrual cramps in a cohort of college women. *British Journal of Obstetrics & Gynaecology* 1996;**103**:1134-42.

24. Hewison A,.van den Akker OB. Dysmenorrhoea, menstrual attitude and GP consultation. *British Journal of Nursing* 1996;**5**:480-4.

25. Mathias SD, Kuppermann M, Liberman RF, Lipschutz RC, Steege JF. Chronic pelvic pain: prevalence, health-related quality of life, and economic correlates. *Obstet Gynecol* 1996;**87**:321-7.

26. Montero P, Bernis C, Fernandez V, Castro S. Influence of body mass index and slimming habits on menstrual pain and cycle irregularity. *Journal of Biosocial Science* 1996;**28**:315-23.

27. Skierska E, Leszczynska-Bystrzanowska J, Gajewski AK. [Risk analysis of menstrual disorders in young women from urban population]. [Polish]. *Przeglad Epidemiologiczny* 1996;**50**:467-74.

28. Vicdan K, Kukner S, Dabakoglu T, Ergin T, Keles G, Gokmen O. Demographic and epidemiologic features of female adolescents in Turkey. *Journal of Adolescent Health* 1996;**18**:54-8.

29. Christiani D, Niu T, Xu X. Occupational stress and Dysmenorrhea in Women Working in Cotton Textile Mills. *Int J Occup Environ Health* 1995;**1**:9-15.

30. Jarrett M, Heitkemper MM, Shaver JF. Symptoms and self-care strategies in women with and without dysmenorrhea. *Health Care Women Int* 1995;**16**:167-78.

31. Onatra HW,.Posso VHJ. Dysmenorrhea behavior in three groups of teenagers belonging to a different social status. *Revista Colombiana de Obstetricia y Ginecologia* 1994;**45**:249-54.

32. Messing K, Saurel-Cubizolles MJ, Bourgine M, Kaminski M. Factors associated with dysmenorrhea among workers in French poultry slaughterhouses and canneries. *Journal of Occupational Medicine* 1993;**35**:493-500.

33. Ng TP, Tan NC, Wansaicheong GK. A prevalence study of dysmenorrhoea in female residents aged 15-54 years in Clementi Town, Singapore. *Annals of the Academy of Medicine, Singapore* 1992;**21**:323-7.

34. Cronje HS,.Kritzinger IE. Menstruation: symptoms, management and attitudes in university students. *Int J Gynaecol Obstet* 1991;**35**:147-50.

35. Izzo A,.Labriola D. Dysmenorrhoea and sports activities in adolescents. *Clinical & Experimental Obstetrics & Gynecology* 1991;**18**:109-16.

36. Lee KA,.Rittenhouse CA. Prevalence of perimenstrual symptoms in employed women. *Women & Health* 1991;**17**:17-32.

37. Odujinrin OM,.Ekunwe EO. Epidemiologic survey of menstrual patterns amongst adolescents in Nigeria. *West African Journal of Medicine* 1991;**10**:244-9.

38. Robinson JC, Plichta S, Weisman CS, Nathanson CA, Ensminger M. Dysmenorrhea and use of oral contraceptives in adolescent women attending a family planning clinic. *American Journal of Obstetrics & Gynecology* 1992;**166**:578-83.

39. Bukman A, Hemelhorst FM, Hengeveld MW. Prevalence and experience of dysmenorrhea in infertility patients. *J psychosom obstet gynaecol* 1990;**11**:147-53.

40. Holmlund U. The experience of dysmenorrhea and its relationship to personality variables. *Acta Psychiatr.Scand.* 1990;**82**:182-7.

41. Okonofua FE, Balogun JA, Ayangade SO, Fawole JO. Exercise and menstrual function in Nigerian university women. *African Journal of Medicine & Medical Sciences* 1990;**19**:185-90.

42. Thomas KD, Okonofua FE, Chiboka O. A study of the menstrual patterns of adolescents in Ile-Ife, Nigeria. *International Journal of Gynaecology & Obstetrics* 1990;**33**:31-4.

43. Sundell G, Milsom I, Andersch B. Factors influencing the prevalence and severity of dysmenorrhoea in young women. *BJOG* 1990;**97**:588-94.

44. Bang RA, Bang AT, Baitule M, Choudhary Y, Sarmukaddam S, Tale O. High prevalence of gynaecological diseases in rural Indian women. *Lancet* 1989;**1**:85-8.

45. Fisher M, Trieller K, Napolitano B. Premenstrual symptoms in adolescents. *J Adolesc.Health Care* 1989;**10**:369-75.

46. Teperi J,.Rimpela M. Menstrual pain, health and behaviour in girls. *Social Science & Medicine* 1989;**29**:163-9.

47. Busch CM, Costa Jr PT, Whitehead WE, Heller BR. Severe perimenstrual symptoms: Prevalence and effects on absenteeism and health care seeking in a non-clinical sample. *Women & Health* 1988;**14**:59-74.

48. Clarvit SR. Stress and menstrual dysfunction in medical students. *Psychosomatics* 1988;**29**:404-9.

49. Johnson J. Level of knowledge among adolescent girls regarding effective treatment for dysmenorrhea. *J Adolesc Health* 1988;**9**:398-402.

50. Pullon S, Reinken J, Sparrow M. Prevalance of dysmenorrhoea in Wellington women. *New Zealand Medical Journal* 1988;**101**:52-4.

51. Gruber VA,.Wildman BG. The impact of dysmenorrhea on daily activities. *Behav.Res.Ther.* 1987;**25**:123-8.

52. Silberg JL, Martin NG, Heath AC. Genetic and environmental factors in primary dysmenorrhea and its relationship to anxiety, depression, and neuroticism. *Behav.Genet.* 1987;**17**:363-83.

53. Toriola AL,.Mathur DN. Menstrual dysfunction in Nigerian athletes. *British Journal of Obstetrics & Gynaecology* 1986;**93**:979-85.

54. Flug D, Largo RH, Prader A. Symptoms related to menstruation in adolescent Swiss girls: a longitudinal study. *Ann.Hum.Biol* 1985;**12**:161-8.

55. Whorwell PJ, McCallum M, Creed FH, Roberts CT. Non-colonic features of irritable bowel syndrome. *Gut* 1986;**27**:37-40.

56. Lemasters GK, Hagen A, Samuels SJ. Reproductive outcomes in women exposed to solvents in 36 reinforced plastics companies. I. Menstrual dysfunction. *J Occup Med* 1985;**27**:490-4.

57. Mergler D,.Vezina N. Dysmenorrhea and cold exposure. *Journal of Reproductive Medicine* 1985;**30**:106-11.

58. Scambler A,.Scambler G. Menstrual symptoms, attitudes and consulting behaviour. *Soc Sci.Med* 1985;**20**:1065-8.

59. Wilson CA,.Keye WR, Jr. A survey of adolescent dysmenorrhea and premenstrual symptom frequency. A model program for prevention, detection, and treatment. *Journal of Adolescent Health Care* 1989;**10**:317-22.

60. Andersch B,.Milsom I. An epidemiologic study of young women with dysmenorrhea. *Am J Obstet Gynecol* 1982;**144**:655-60.

61. Woods NF, Most A, Dery GK. Prevalence of perimenstrual symptoms. *Am J Public Health* 1982;**72**:1257-64.

62. Klein JR,.Litt IF. Epidemiology of adolescent dysmenorrhea. *Pediatrics* 1981;**68**:661-4.

63. Svanberg L,.Ulmsten U. The incidence of primary dysmenorrhea in teenagers. *Archives of Gynecology* 1981;**230**:173-7.

64. Iglesias R, Terres A, Chavarria A. Disorders of the menstrual cycle in airline stewardesses. *Aviat.Space Environ Med* 1980;**51**:518-20.

65. Sogbanmu MO,.Aregbesola YA. Menarchal age in Nigerian schoolgirls: its relationship to their height, weight and menstrual profile. *Int J Gynaecol Obstet* 1978;**16**:339-40.

66. Webster SK, Martin HJ, Uchalik D, Gannon L. The Menstrual Symptom Questionnaire and spasmodic/congestive dysmenorrhea: measurement of an invalid construct. *J Behav Med* 1979;**2**:1-19.

67. Widholm O, Frisk M, Tenhunen T, Hortling H. Gynecological findings in adolescence. A study of 514 patients. *Acta Obstet Gynecol Scand.* 1967;**46**:Suppl-27.

68. Widholm O,.Kantero R. A statistical analysis of the menstrual patterns of 8000 Finnish girls and their mothers. *Acta Obstet Gynecol Scand* 1971;**14**:1-36.

69. Widholm O. Dysmenorrhea during adolescence. *Acta Obstet Gynecol Scand Suppl* 1979;**87**:61-6.

70. Wood C, Larsen L, Williams R. Menstrual characteristics of 2,343 women attending the Shepherd foundation. *Australian and New Zealand Journal of Obstetrics and Gynaecology, Vol 19(2) (pp 107-110), 1979* 1979.

71. Khatri R,.Gupta AN. Effect of childbirth on menstrual pattern. *Indian J Med Res.* 1978;**67**:66-72.

72. Malina RM, Spirduso WW, Tate C, Baylor AM. Age at menarche and selected menstrual characteristics in athletes at different competitive levels and in different sports. *Medicine & Science in Sports* 1978;**10**:218-22.

73. Sheldrake P,.Cormack M. Variations in menstrual cycle symptom reporting. *J Psychosom.Res.* 1976;**20**:169-77.

74. Timonen S,.Procope BJ. The premenstrual syndrome; frequency and association of symptoms. *Ann.Chir Gynaecol Fenn.* 1973;**62**:108-16.

75. Bergsjo P, Jenssen H, Vellar OD. Dysmenorrhea in industrial workers. *Acta Obstet Gynecol Scand.* 1975;**54**:255-9.

76. Sehgal K, Marwah S, Tiwari I. Symptoms associated with menstruation and some correlates of dysmenorrhoea in college girls. *Journal of obstetrics and gynaecology of India* 1972;**22**:323-9.

77. Theano G. The prevalence of menstrual symptoms in Spanish students. *Br J Psychiatry* 1968;**114**:771-3.

78. Hirt M, Kurtz R, Ross WD. The relationship between dysmenorrhea and selected personality variables. *Psychosomatics* 1967;**8**:350-3.

79. Golub LJ., Lang WR, Menduke H. The incidence of dysmenorrhea in high school girls. *Postgrad.Med* 1958;**23**:38-40.

80. Hirata M, Kumabe K, Inoue Y. Relationship between the frequency of menstrual pain and bodyweight in female adolescents. [Japanese]. *Nippon Koshu Eisei Zasshi - Japanese Journal of Public Health* 2002;**49**:516-24.

81. Mijanovic D. [Correlation between certain factors in maturation and primary dysmenorrhea in adolescence]. [Serbo-Croatian (Roman)]. *Jugoslavenska Ginekologija i Perinatologija* 1990;**30**:79-82.

82. Yang JM, Chen QY, Jiang XZ. Effects of metallic mercury on the perimenstrual symptoms and menstrual outcomes of exposed workers. *Am J Ind.Med* 2002;**42**:403-9.

83. Shye D,.Jaffe B. Prevalence and correlates of perimenstrual symptoms: a study of Israeli teenage girls. *J Adolesc Health* 1991;**12**:217-24.

1. González Bahamonde, Miriam and Ibarra Farías, Mario. Conocimientos y prácticas de autocuidado sobre síndrome premenstrual y dismenorrea de un grupo de alumnas de la Facultad de Educación de la Pontificia Universidad Católica de Chile / Selfcare knowledge and practice about premenstrual syndrome and dysmenorrea in a group of female students from Facultad de Educación, Pontificia Universidad Católica de Chile. 83. 1999. Ref Type: Thesis/Dissertation

85. Barros ACSDd, Takemoto AK, Coronado MRG, Marques JA, Nishimura A. Caracterizaçäo epidemiológica da dismenorréia / Dysmenorrhea epidemiological characterization . *Rev.IATROS* 1987;**6**:13-9.

86. Rojas J, Robles C, Rojas N. Dismenorrea en la adolescencia. *Rev.colomb.obstet.ginecol* 1997;**48**:95-105.

87. Nuñez Troconis JT. Trastornos menstruales en estudiantes universitarias: II Menarquia y dismenorrea / Menstrual upsetting in the female universitary student: II Menarch and Dysmenorrhea . *Rev.obstet.ginecol.Venezuela* 1991;**51**:105-8.

88. Peña Nina DE, Mora Cabrera RA, Chalas A. La dismenorrea como causa de ausentismo y bajo rendimiento laboral / Dismenorrea : cause of absence and low production at work . *Acta méd.domin* 1993;**15**:92-6.

89. El Defrawi, Dandash KF, Refaat AH, Eyada M. Female genital mutilation and its psychosexual impact. *Journal of Sex & Marital Therapy* 2001;**27**:465-73.

90. Gurel H,.Gurel SA. Dyspareunia, back pain and chronic pelvic pain: The importance of this pain complex in gynecological practice and its relation with grandmultiparity and pelvic relaxation. *Gynecologic & Obstetric Investigation* 1999;**48**:119-22.

91. Jamieson DJ,.Steege JF. The prevalence of dysmenorrhea, dyspareunia, pelvic pain, and irritable bowel syndrome in primary care practices. *Obstet Gynecol* 1996;**87**:55-8.

92. Shah PN, Smith JR, Wells C, Barton SE, Kitchen VS, Steer PJ. Menstrual symptoms in women infected by the human immunodeficiency virus. *Obstet Gynecol* 1994;**83**:397-400.

93. Taner CE, Hakverdi AU, Erden AC, Satici O. Menstrual disorders and pelvic pain after sterilization. *Adv.Contracept.* 1995;**11**:309-15.

94. Rulin MC, Davidson AR, Philliber SG, Graves WL, Cushman LF. Long-term effect of tubal sterilization on menstrual indices and pelvic pain. *Obstet Gynecol* 1993;**82**:118-21.

95. Mahmood TA, Templeton AA, Thomson L, Fraser C. Menstrual symptoms in women with pelvic endometriosis. *BJOG* 1991;**98**:558-63.

96. Walker EA, Katon WJ, Jemelka R, Alfrey H, Bowers M, Stenchever MA. The prevalence of chronic pelvic pain and irritable bowel syndrome in two university clinics. *J Psychosom Obstet Gynecol* 1991;**12**:65-75.

97. Stambolov B. [Adnexal inflammatory diseases and their influence on menstrual function]. [Bulgarian]. *Akusherstvo i Ginekologiia* 1989;**28**:35-7.

98. Cavanaugh RM, Jr. Obtaining a personal and confidential history from adolescents. An opportunity for prevention. *J Adolesc Health Care* 1986;**7**:118-22.

99. Liu DT,.Hitchcock A. Endometriosis: its association with retrograde menstruation, dysmenorrhoea and tubal pathology. *BJOG* 1986;**93**:859-62.

100. Sobczyk R, Braunstein ML, Solberg L, Schuman SH. A case control survey and dysmenorrhea in a family practice population: a proposed disability index. *J Fam Pract* 1978;**7**:285-90.

101. Kessel N,.Coppen A. The prevalence of common menstrual symptoms. *Lancet* 1963;**2**:61-4.

102. Clow AES. Discussion on dysmenorrhea in young women: its incidence, prevention and treatment. *BMJ* 1924;**2**:558-61.

103. Gray LA. Gynecology in adolescence. *Pediatric clinics of North America* 1960;**7**:43-63.

104. Abdo CH, Oliveira WM, Jr., Moreira ED, Jr., Fittipaldi JA. Prevalence of sexual dysfunctions and correlated conditions in a sample of Brazilian women--results of the Brazilian study on sexual behavior (BSSB). *Int J Impot Res* 2004;**16**:160-6.

105. Johnson SD, Phelps DL, Cottler LB. The association of sexual dysfunction and substance use among a community epidemiological sample. *Arch Sex Behav* 2004;**33**:55-63.

106. Oberg K, Fugl-Meyer AR, Fugl-Meyer KS. On categorization and quantification of women's sexual dysfunctions: an epidemiological approach. *Int J Impot Res* 2004;**16**:261-9.

107. Danielsson I, Sjoberg I, Stenlund H, Wikman M. Prevalence and incidence of prolonged and severe dyspareunia in women: results from a population study. *Scandinavian Journal of Public Health* 2003;**31**:113-8.

108. Cain VS, Johannes CB, Avis NE, Mohr B, Schocken M, Skurnick J *et al*. Sexual functioning and practices in a multi-ethnic study of midlife women: Baseline results from SWAN. *Journal of Sex Research* 2003;**40**:266-76.

109. Desai VK, Kosambiya JK, Thakor HG, Umrigar DD, Khandwala BR, Bhuyan KK. Prevalence of sexually transmitted infections and performance of STI syndromes against aetiological diagnosis, in female sex workers of red light area in Surat, India. *Sexually Transmitted Infections* 2003;**79**:111-5.

110. Kadri N, McHichi Alami KH, McHakra TS. Sexual dysfunction in women: population based epidemiological study. *Archives of Women's Mental Health* 2002;**5**:59-63.

111. Nazareth I, Boynton P, King M. Problems with sexual function in people attending London general practitioners: cross sectional study. *BMJ* 2003;**327**:423.

112. Avis NE, Stellato R, Crawford S, Johannes C, Longcope C. Is there an association between menopause status and sexual functioning? *Menopause* 2000;**7**:297-309.

113. Zhao G, Wang L, Yan R, Dennerstein L. Menopausal symptoms: experience of Chinese women. *Climacteric* 2000;**3**:135-44.

114. Bhurt, AW, Fikree A, Channa GZ, Soomro R, Bhurt n. Prevalence and Risk Factors of Symptoms of Pelvic Inflammatory Disease in a Rural Community of Jamshoro, Sindh, Pakistan. *J Pak Med Assoc* 1999;**49**:188-94.

115. Dunn KM, Croft PR, Hackett GI. Association of sexual problems with social, psychological, and physical problems in men and women: a cross sectional population survey. *Journal of Epidemiology & Community Health* 1999;**53**:144-8.

116. Laumann EO, Paik A, Rosen RC. Sexual dysfunction in the United States: prevalence and predictors. *JAMA* 1999;**281**:537-44.

117. Ventegodt S. Sex and the quality of life in Denmark. *Arch.Sex Behav.* 1998;**27**:295-307.

118. Barlow DH. UK prevalence study of urogenital ageing. *Round Table Series - Royal Society of Medicine* 1995;**Issue 38**.

119. Stenberg A, Heimer G, Ulmsten U, Cnattingius S. Prevalence of genitourinary and other climacteric symptoms in 61-year-old women. *Maturitas* 1996;**24**:31-6.

120. Ramoso-Jalbuena J. Climacteric Filipino women: a preliminary survey in the Philippines. *Maturitas* 1994;**19**:183-90.

121. Ernst C, Foldenyi M, Angst J. The Zurich Study: XXI. Sexual dysfunctions and disturbances in young adults. Data of a longitudinal epidemiological study. *European Archives of Psychiatry & Clinical Neuroscience* 1993;**243**:179-88.

122. Lindal E,.Stefansson JG. The lifetime prevalence of psychosexual dysfunction among 55 to 57-year-olds in Iceland. *Social Psychiatry & Psychiatric Epidemiology* 1993;**28**:91-5.

123. Rekers H, Drogendijk AC, Valkenburg HA, Riphagen F. The menopause, urinary incontinence and other symptoms of the genito-urinary tract. *Maturitas* 1992;**15**:101-11.

124. Glatt AE, Zinner SH, McCormack WM. The prevalence of dyspareunia. *Obstet Gynecol* 1990;**75**:433-6.

125. Berg G, Gottwall T, Hammar M, Lindgren R, Gottgall T. Climacteric symptoms among women aged 60-62 in Linkoping, Sweden, in 1986. *Maturitas* 1988;**10**:193-9.

126. Osborn M, Hawton K, Gath D. Sexual dysfunction among middle aged women in the community. *BMJ* 1988;**British Medical Journal. 296**:959-62.

127. Castelo-Branco C, Blumel JE, Araya H, Riquelme R, Castro G, Haya J *et al*. Prevalence of sexual dysfunction in a cohort of middle-aged women: Influences of menopause and hormone replacement therapy. *Journal of Obstetrics & Gynaecology* 2003;**23**:426-30.

128. Iosif CS,.Bekassy Z. Prevalence of genito-urinary symptoms in the late menopause. *Acta Obstet Gynecol Scand.* 1984;**63**:257-60.

129. Garde K,.Lunde I. Female sexual behaviour. A study in a random sample of 40-year-old women. *Maturitas* 1980;**2**:225-40.

130. Danaci AE, Oruc S, Adiguzel H, Yildirim Y, Aydemir O. Relationship of sexuality with psychological and hormonal features in the menopausal period. *West Indian Medical Journal* 2003;**52**:27-30.

131. Nappi RE, Verde JB, Polatti F, Genazzani AR, Zara C. Self-reported sexual symptoms in women attending menopause clinics. *Gynecologic & Obstetric Investigation* 2002;**53**:181-7.

132. Versi E, Harvey MA, Cardozo L, Brincat M, Studd JW. Urogenital prolapse and atrophy at menopause: a prevalence study. *International Urogynecology Journal* 2001;**12**:107-10.

133. Shokrollahi P, Mirmohamadi M, Mehrabi F, Babaei G. Prevalence of sexual dysfunction in women seeking services at family planning centers in Tehran. *Journal of Sex & Marital Therapy* 1999;**25**:211-5.

134. Ismael NN. A study on the menopause in Malaysia. *Maturitas* 1994;**19**:205-9.

135. Rosen RC, Taylor JF, Leiblum SR, Bachmann GA. Prevalence of sexual dysfunction in women: results of a survey study of 329 women in an outpatient gynecological clinic. *Journal of Sex & Marital Therapy* 1993;**19**:171-88.

136. Pepe F, Garozzo G, Pepe P. Incidence of sexual dysfunction in healthy sexually active Sicilian women of fertile age spontaneously attending a private gynaecologist for routine examination. *Journal of Obstetrics & Gynaecology* 1991;**11**:277-80.

137. Dudley Chapman J. A longitudinal study of sexuality and gynecologic health in abused women. *Journal of American Osteopathic Association* 1989;**89**:619-23.

138. Bachmann GA, Leiblum SR, Grill J. Brief sexual inquiry in gynecologic practice. *Obstet Gynecol* 1989;**73**:425-7.

139. Schein M, Zyzanski SJ, Levine S, Medalie JH, Dickman RL, Alemagno SA. The frequency of sexual problems among family practice patients. *Fam.Pract.Res.J* 1988;**7**:122-34.

140. Warner P. Psychiatric disorder and gynaecological symptoms in middle aged women. *Br Med J (Clin Res Ed)* 1987;**294**:1033-4.

141. Heisterberg L. Factors influencing spontaneous abortion, dyspareunia, dysmenorrhea, and pelvic pain. *Obstet Gynecol* 1993;**81**:594-7.

142. Plouffe L, Jr. Screening for sexual problems through a simple questionnaire. *Am J Obstet Gynecol* 1985;**151**:166-9.

143. Buddeberg C, Hess D, Merz J. Sexuelle Probleme von Patienten in der Allgemeinpraxis.[Sexual problems of patients in general practice]. *Schweiz Rundsch.Med Prax.* 1984;**73**:1113-8.

144. Zondervan KT, Yudkin PL, Vessey MP, Dawes MG, Barlow DH, Kennedy SH. Prevalence and incidence of chronic pelvic pain in primary care: Evidence from a national general practice database. *Br J Obstet Gynaecol* 1999;**106**:1149-55.

145. Filippi V, Marshall T, Bulut A, Graham W, Yolsal N. Asking questions about women's reproductive health: validity and reliability of survey findings from Istanbul. *Trop.Med Int Health* 1997;**2**:47-56.

146. Frljak A, Cengic S, Hauser M, Schei B. Gynecological complaints and war traumas. A study from Zenica, Bosnia-Herzegovina during the war. *Acta Obstetricia et Gynecologica Scandinavica* 1997;**76**:350-4.

147. Bhatia JC,.Cleland J. Self-reported symptoms of gynecological morbidity and their treatment in south India. *Studies in Family Planning* 1995;**26**:203-16.

148. Kirkengen AL, Schei B, Steine S. Indicators of childhood sexual abuse in gynaecological patients in a general practice. *Scand.J Prim.Health Care* 1993;**11**:276-80.
