# Supplementary material for: WHO systematic review of prevalence of chronic pelvic pain: a neglected reproductive health morbidity
Source: BMC Public Health. 2006 Jul 6;6:177. doi: 10.1186/1471-2458-6-177 (PMC1550236; doi:10.1186/1471-2458-6-177)
Supplement: Additional file 3 — Appendix of excluded studies [file 1471-2458-6-177-S3.doc]

# Appendix of Studies excluded

# No/ Insufficient /unclear data in study for analysis^1-5^

# Not a primary data source^6-24^

# Not on prevalence of pelvic pain^25-74^

# Duplicate data^75-83^

# Study performed in pregnant/postnatal women^84-91^ or in women with other disorders^92-95^ including cancer^96-99^ or women not representative of the general population^100-102^

Comment/letter^103^/discussion/ case-control study^104^/case report^105;106^

Not on file/unobtainable^107-109^

Reference List

1. Alonso C,.Coe CL. Disruptions of social relationships accentuate the association between emotional distress and menstrual pain in young women. *Health Psychology* 2001;**20**:411-6.

2. Deutch B, Jorgensen EB, Hansen JC. Menstrual discomfort in danish women reduced by dietary supplements of omega-3 PUFA and B12 (fish oil or seal oil capsules). *Nutrition Research* 2000;**20**:621-31.

3. Slatford K,.Currie C. Prevalence of psychosexual problems in patients attending a genitourinary clinic. *Br J Vener.Dis* 1984;**60**:398-401.

4. Gomes KRO,.d'A Tanaka AC. Reported morbidity and use of health services by working women, Brazil. *Revista de Saude Publica* 2003;**37**:75-82.

5. Salonia A, Zanni G, Nappi RE, Briganti A, Deho F, Fabbri F *et al*. Sexual dysfunction is common in women with lower urinary tract symptoms and urinary incontinence: results of a cross-sectional study. *Eur Urol.* 2004;**45**:642-8.

6. Bernardini P, Bondavalli C, Luciano M, Schiavon L, Dall'Oglio B, Parma P *et al*. [Interstitial cystitis: epidemiology]. [Italian]. *Archivio Italiano di Urologia, Andrologia* 1999;**71**:313-5.

7. Binik YM, Meana M, Berkley K, Khalife S. The sexual pain disorders: is the pain sexual or is the sex painful?[erratum appears in Annu Rev Sex Res 2000;11:286]. [Review] [96 refs]. *Annual Review of Sex Research* 1999;**10**:210-35.

8. Meana M,.Binik YM. Painful coitus: a review of female dyspareunia. [Review] [89 refs]. *Journal of Nervous & Mental Disease* 1994;**182**:264-72.

9. Cramer DW,.Missmer SA. The epidemiology of endometriosis. *Annals of the New York Academy of Sciences* 396;**955**:11-22.

10. Davis AR,.Westhoff CL. Primary dysmenorrhea in adolescent girls and treatment with oral contraceptives.[comment]. [Review] [40 refs]. *Journal of Pediatric & Adolescent Gynecology* 2001;**14**:3-8.

11. Dunn KM, Jordan K, Croft PR, Assendelft WJ. Systematic review of sexual problems: epidemiology and methodology. [Review] [46 refs]. *Journal of Sex & Marital Therapy* 2002;**28**:399-422.

12. Fisher M, Trieller K, Napolitano B. Premenstrual symptoms in adolescents. [Review] [27 refs]. *Journal of Adolescent Health Care* 1989;**10**:369-75.

13. Gelbaya TA,.El Halwagy HE. Focus on primary care: chronic pelvic pain in women. [Review] [57 refs]. *Obstetrical & Gynecological Survey* 2001;**56**:757-64.

14. Heim LJ. Evaluation and differential diagnosis of dyspareunia. [Review] [27 refs]. *American Family Physician* 2001;**63**:1535-44.

15. Heiman JR. Sexual dysfunction: Overview of prevalence, etiological factors, and treatments. *Journal of Sex Research* 2002;**39**:73-8.

16. Johnson RA. Diagnosis and treatment of common sexually transmitted diseases in women. *CLIN.CORNERSTONE* 2000;**3**:1-11.

17. Kusek JW,.Nyberg LM. The epidemiology of interstitial cystitis: is it time to expand our definition?. [Review] [32 refs]. *Urology* 2001;**57**:95-9.

18. Lightner DJ. Female sexual dysfunction. [Review] [15 refs]. *Mayo Clinic Proceedings* 2002;**77**:698-702.

19. Reamy KJ,.White SE. Sexuality in the puerperium: a review. *Archives of Sexual Behavior* 1987;**16**:165-86.

20. Spector IP,.Carey MP. Incidence and prevalence of the sexual dysfunctions: a critical review of the empirical literature. [Review] [41 refs]. *Archives of Sexual Behavior* 1990;**19**:389-408.

21. Spector IP, Leiblum SR, Carey MP, Rosen RC. Diabetes and female sexual function: A critical review. *Annals of Behavioral Medicine* 1993;**15**:257-64.

22. Stenberg A, Heimer G, Ulmsten U. The prevalence of urogenital symptoms in postmenopausal women. [Review] [22 refs]. *Maturitas* 1995;**22 Suppl**:S17-S20.

23. Zondervan K,.Barlow DH. Epidemiology of chronic pelvic pain. [Review] [37 refs]. *Best Practice & Research in Clinical Obstetrics & Gynaecology* 2000;**14**:403-14.

24. Zondervan KT, Yudkin PL, Vessey MP, Dawes MG, Barlow DH, Kennedy SH. The prevalence of chronic pelvic pain in women in the United Kingdom: A systematic review. *British Journal of Obstetrics & Gynaecology* 1998;**105**:93-9.

25. World Health Organization multicenter study on menstrual and ovulatory patterns in adolescent girls. II. Longitudinal study of menstrual patterns in the early postmenarcheal period, duration of bleeding episodes and menstrual cycles. World Health Organization Task Force on Adolescent Reproductive Health. *J Adolesc.Health Care* 1986;**7**:236-44.

26. Prevalence and anatomical distribution of endometriosis in women with selected gynaecological conditions: results from a multicentric Italian study. Gruppo italiano per lo studio dell'endometriosi. *Human Reproduction* 1994;**9**:1158-62.

27. Adelman AM, Revicki DA, Magaziner J, Hebel R. Abdominal pain in an HMO. *Family Medicine* 1995;**27**:321-5.

28. Agreus L, Svardsudd K, Nyren O, Tibblin G. The epidemiology of abdominal symptoms: prevalence and demographic characteristics in a Swedish adult population. A report from the Abdominal Symptom Study. *Scandinavian Journal of Gastroenterology* 1994;**29**:102-9.

29. Ajossa S, Mais V, Guerriero S, Paoletti AM, Caffiero A, Murgia C *et al*. The prevalence of endometriosis in premenopausal women undergoing gynecological surgery. *Clinical & Experimental Obstetrics & Gynecology* 1994;**21**:195-7.

30. Al Qutob R. Menopause-associated problems: types and magnitude. A study in the Ain Al-Basha area, Jordan. *Journal of Advanced Nursing* 2001;**33**:613-20.

31. Boey C, Yap S, Goh KL. The prevalence of recurrent abdominal pain in 11- to 16-year-old Malaysian schoolchildren. *Journal of Paediatrics & Child Health* 2000;**36**:114-6.

32. Clemons JL, Arya LA, Myers DL. Diagnosing interstitial cystitis in women with chronic pelvic pain. *OBSTET GYNECOL* 2002;**100**:337-41.

33. Collins A,.Landgren BM. Reproductive health, use of estrogen and experience of symptoms in perimenopausal women: a population-based study. *Maturitas* 1994;**20**:101-11.

34. Dalton VK, Haefner HK, Reed BD, Senapati S, Cook A. Victimization in patients with vulvar dysesthesia/vestibulodynia: Is there an increased prevalence? *Journal of Reproductive Medicine for the Obstetrician & Gynecologist* 2002;**47**:829-34.

35. Boey CCM,.Goh K-L. Predictors of recurrent abdominal pain among 9 to 15-year-old urban school-children in Malaysia. *Acta Paediatrica* 2001;**90**:353-5.

36. Deeb ME, Campbell OM, Kabakian TK. Safe motherhood in Lebanon: new population-based results from the Beirut 1994 survey. *Int J Gynaecol Obstet* 1997;**56**:181-2.

37. Dennerstein L, Dudley E, Burger H. Are changes in sexual functioning during midlife due to aging or menopause? *Fertility & Sterility* 2001;**76**:456-60.

38. Doezema D, Hepworth E, Young SA, Arguelles CA, Brillman JC, Tandberg D. Low prevelance of Chlamydia trachomatis by urinary ligase chain reaction in women patients in the emergency department. *Academic Emergency Medicine* 2002;**9**:646-9.

39. Dusek T. Influence of high intensity training on menstrual cycle disorders in athletes. *Croatian Medical Journal* 2001;**42**:79-82.

40. Edgardh K,.Abdelnoor M. Longstanding vulval problems and entry dyspareunia among STD-clinic visitors in Oslo-results from a cross-sectional study. *Int J STD AIDS* 2003;**14**:796-9.

41. Essau CA, Conradt J, Petermann F. Prevalence, comorbidity and psychosocial impairment of somatoform disorders in adolescents. *Psychology Health & Medicine* 1999;**4**:169-80.

42. Fontana D,.Rees V. Primary dysmenorrhea, educational performance, and cognitive and affective variables in adolescent schoolgirls. *Br J Educ.Psychol.* 1982;**52**:199-204.

43. Frank E, Anderson C, Rubinstein D. Frequency of sexual dysfunction in "normal" couples. *N Engl.J Med* 1978;**299**:111-5.

44. Golden JS, Golden M, Price S, Heinrich A. The sexual problems of family planning clinic patients as viewed by the patients and the staff. *Fam.Plann.Perspect.* 1977;**9**:25-9.

45. Greimel ER, Dorfer M, Schaffer M, Winter R. Medical and psychological symptoms of sexually abused females. *Geburtshilfe und Frauenheilkunde, Vol 59(4) (pp 180-186),* 1999.

46. Halder SL, McBeth J, Silman AJ, Thompson DG, Macfarlane GJ. Psychosocial risk factors for the onset of abdominal pain. Results from a large prospective population-based study. *International Journal of Epidemiology* 2002;**31**:1219-25.

47. Harlow BL, Wise LA, Stewart EG. Prevalence and predictors of chronic lower genital tract discomfort. *American Journal of Obstetrics & Gynecology* 2001;**185**:545-50.

48. Heitkemper MM, Shaver JF, Mitchell ES. Gastrointestinal symptoms and bowel patterns across the menstrual cycle in dysmenorrhea. *NURS.RES.* 1988;**37**:108-13.

49. James FR, Large RG, Bushnell JA, Wells JE. Epidemiology of pain in New Zealand. *Pain* 1991;**44**:279-83.

50. Jensen JT, Wilder K, Carr K, Romm J, Hansen A. Quality of life and sexual function after evaluation and treatment at a referral center for vulvovaginal disorders. *American Journal of Obstetrics & Gynecology* 2003;**188**:1629-35.

51. Kay L, Jorgensen T, Schultz-Larsen K. Abdominal pain in a 70-year-old Danish population. An epidemiological study of the prevalence and importance of abdominal pain. *Journal of Clinical Epidemiology* 1992;**45**:1377-82.

52. Kay L, Jorgensen T, Jensen KH. Epidemiology of abdominal symptoms in a random population: prevalence, incidence, and natural history. *European Journal of Epidemiology* 1994;**10**:559-66.

53. Kroenke K,.Price RK. Symptoms in the community: Prevalence, classification, and psychiatric comorbidity. *Archives of Internal Medicine* 1993;**153**:2474-80.

54. Laufer MR, Goitein L, Bush M, Cramer DW, Emans SJ. Prevalence of endometriosis in adolescent girls with chronic pelvic pain not responding to conventional therapy. *Journal of Pediatric & Adolescent Gynecology* 1997;**10**:199-202.

55. Mens JMA, Vleeming A, Snijders CJ, Koes BW, Stam HJ. Validity of the active straight leg raise test for measuring disease severity in patients with posterior pelvic pain after pregnancy. *Spine* 2002;**27**:196-200.

56. Moen MH,.Schei B. Epidemiology of endometriosis in a Norwegian county. *Acta Obstetricia et Gynecologica Scandinavica* 1997;**76**:559-62.

57. Nusbaum MR, Gamble G, Skinner B, Heiman J. The high prevalence of sexual concerns among women seeking routine gynecological care. *Journal of Family Practice* 2000;**49**:229-32.

58. Nusbaum MR,.Gamble G. The prevalence and importance of sexual concerns among female military beneficiaries. *Military Medicine* 2001;**166**:208-10.

59. Ostgaard HC, Andersson GBJ, Wennergren M. The impact of low back and pelvic pain in pregnancy on the pregnancy outcome. *Acta Obstetricia et Gynecologica Scandinavica, Vol 70(1) (pp 21-24), 1991* 1991.

60. Parazzini F, Luchini L, Vezzoli F, Mezzanotte C, Vercellini P, Romanini C *et al*. Prevalence and anatomical distribution of endometriosis in women with selected gynaecological conditions: Results from a multicentric Italian study. *Human Reproduction* 1994;**9**:1158-62.

61. Parsons CL, Bullen M, Kahn BS, Stanford EJ, Willems JJ. Gynecologic presentation of interstitial cystitis as detected by intravesical potassium sensitivity. *OBSTET GYNECOL* 2001;**98**:127-32.

62. Reese KA, Reddy S, Rock JA. Endometriosis in an adolescent population: the Emory experience. *Journal of Pediatric & Adolescent Gynecology* 1996;**9**:125-8.

63. Romans S, Belaise C, Martin J, Morris E, Raffi A. Childhood abuse and later medical disorders in women. An epidemiological study. *Psychotherapy & Psychosomatics* 2002;**71**:141-50.

64. Ryding EL. Sexuality during and after pregnancy. *Acta Obstetricia et Gynecologica Scandinavica* 1984;**63**:679-82.

65. Samil RS,.Wishnuwardhani SD. Health of Indonesian women city-dwellers of perimenopausal age. *Maturitas* 1994;**19**:191-7.

66. Sinha AK, Agarwal A, Lakhey M, Mishra A, Sah SP. Incidence of pelvic and extrapelvic endometriosis in Eastern region of Nepal. *Indian Journal of Pathology & Microbiology* 2003;**46**:20-3.

67. Stout AL, Steege JF, Dodson WC, Hughes CL. Relationship of laparoscopic findings to self-report of pelvic pain. *Am J Obstet Gynecol* 1991;**164**:73-9.

68. Tsekov V,.Duchev S. [The characteristics of the spontaneous realization of sexual intercourse in economically employed women]. [Bulgarian]. *Akusherstvo i Ginekologiia* 1990;**29**:40-5.

69. Van Lankveld JJDM,.Grotjohann Y. Psychiatric comorbidity in heterosexual couples with sexual dysfunction assessed with the Composite International Diagnostic Interview. *Archives of Sexual Behavior* 2000;**29**:479-98.

70. Vazquez-Benitez E, Garrido-Latorre F, MacGregor C, Tamayo-Orozco J, Lopez-Carrillo L, Parra S *et al*. [Reproducibility of a questionnaire for studying climacteric]. [Spanish]. *Salud Publica de Mexico* 1996;**38**:363-70.

71. Vercellini P, Fedele L, Arcaini L, Bianchi S, Rognoni MT, Candiani GB. Laparoscopy in the diagnosis of chronic pelvic pain in adolescent women. *Journal of Reproductive Medicine* 1989;**34**:827-30.

72. Wasserheit JN, Harris JR, Chakraborty J, Kay BA, Mason KJ. Reproductive tract infections in a family planning population in rural Bangladesh. *Stud.Fam.Plann.* 1989;**20**:69-80.

73. Yasmin S,.Yasin KF. Prevalence of 'experience of menopause' in women of 45-55 years of age. *Medical Forum Monthly* 2002;**13**:8-10.

74. Morison L, Scherf C, Ekpo G, Paine K, West B, Coleman R *et al*. The long-term reproductive health consequences of female genital cutting in rural Gambia: A community-based survey. *Tropical Medicine & International Health* 2001;**6**:643-53.

75. Filippi V, Marshall T, Bulut A, Graham W, Yolsal N. Asking questions about women's reproductive health: validity and reliability of survey findings from Istanbul. *Trop.Med Int Health* 1997;**2**:47-56.

76. Frisk M, Widholm O, Hortling H. Dysmenorrhea--psyche and soma in teenagers. *Acta Obstet Gynecol Scand.* 1965;**44**:339-47.

77. Hammar M, Berg G, Fahraeus L, Larsson-Cohn U. Climacteric symptoms in an unselected sample of Swedish women. *Maturitas* 1984;**6**:345-50.

78. Milsom I, Sundell G, Andersch B. The influence of different combined oral contraceptives on the prevalence and severity of dysmenorrhea. *Contraception* 1990;**42**:497-506.

79. Parazzini F, Cipriani S, Moroni S, Crosignani PG. Relationship between stage, site and morphological characteristics of pelvic endometriosis and pain. *Human Reproduction* 2001;**16**:2668-71.

80. Weber AM, Walters MD, Piedmonte MR. Sexual function and vaginal anatomy in women before and after surgery for pelvic organ prolapse and urinary incontinence. *American Journal of Obstetrics & Gynecology* 2000;**182**:1610-5.

81. Woods NF, Most A, Dery GK. Estimating perimenstrual distress: a comparison of two method. *Res.Nurs.Health* 1982;**5**:81-91.

82. Zondervan KT, Yudkin PL, Vessey MP, Jenkinson CP, Dawes MG, Barlow DH *et al*. Chronic pelvic pain in the community - Symptoms, investigations, and diagnoses. *American Journal of Obstetrics & Gynecology* 2001;**184**:1149-55.

83. Zondervan KT, Yudkin PL, Vessey MP, Jenkinson CP, Dawes MG, Barlow DH *et al*. Chronic pelvic pain in the community - Symptoms, investigations, and diagnoses. *American Journal of Obstetrics & Gynecology* 2001;**184**:1149-55.

84. Barrett G, Pendry E, Peacock J, Victor C, Thakar R, Manyonda I. Women's sexual health after childbirth. *BJOG: an International Journal of Obstetrics & Gynaecology* 2000;**107**:186-95.

85. Barrett G, Pendry E, Peacock J, Victor C, Thakar R, Manyonda I. Women's sexuality after childbirth: A pilot study. *Archives of Sexual Behavior* 1999;**28**:179-91.

86. Goetsch MF. Postpartum dyspareunia. An unexplored problem.[comment]. *Journal of Reproductive Medicine* 1999;**44**:963-8.

87. Oboro VO,.Tabowei TO. Sexual function after childbirth in Nigerian women. *International Journal of Gynecology & Obstetrics* 2002;**78**:249-50.

88. Signorello LB, Harlow BL, Chekos AK, Repke JT. Postpartum sexual functioning and its relationship to perineal trauma: a retrospective cohort study of primiparous women

104. *American Journal of Obstetrics & Gynecology* 2001;**184**:881-8.

89. Okonofu FE, Larsen U, Oronsaye F, Snow RC, Slanger TE. The association between female genital cutting and correlates of sexual and gynaecological morbidity in Edo State, Nigeria. *BJOG: an International Journal of Obstetrics & Gynaecology* 2002;**109**:1089-96.

90. Albert HB, Godskesen M, Westergaard JG. Incidence of four syndromes of pregnancy-related pelvic joint pain. *Spine* 2002;**27**:2831-4.

91. Heisterberg L. Factors influencing spontaneous abortion, dyspareunia, dysmenorrhea, and pelvic pain. *OBSTET GYNECOL* 1993;**81**:594-7.

92. Copeland CE, Bosse MJ, McCarthy ML, MacKenzie EJ, Guzinski GM, Hash CS *et al*. Effect of trauma and pelvic fracture on female genitourinary, sexual, and reproductive function. *Journal of Orthopaedic Trauma* 1997;**11**:73-81.

93. Brathwaite AR,.Figueroa JP. Survey of patients with sexually transmitted diseases seen by private physicians in Jamaica. *West Indian Medical Journal* 1997;**46**:43-6.

94. Heiberg EE. Pelvic pain and low back pain in pregnant women - An epidemiological study. *Scandinavian Journal of Rheumatology* 1995;**24**:135-41.

95. Bauer JJ. Sexual dysfunction after colectomy. *Mount Sinai Journal of Medicine* 1983;**50**:187-9.

96. Andersen BL, Anderson B, deProsse C. Controlled prospective longitudinal study of women with cancer: I. Sexual functioning outcomes. *Journal of Consulting & Clinical Psychology* 1989;**57**:683-91.

97. Bergmark K, Avall-Lundqvist E, Dickman PW, Henningsohn L, Steineck G. Vaginal changes and sexuality in women with a history of cervical cancer. *New England Journal of Medicine* 1999;**340**:1383-9.

98. Jensen PT, Groenvold M, Klee MC, Thranov I, Petersen MA, Machin D. Longitudinal study of sexual function and vaginal changes after radiotherapy for cervical cancer. *International Journal of Radiation Oncology, Biology, Physics* 2003;**56**:937-49.

99. Thranov I,.Klee M. Sexuality among gynecologic cancer patients - A cross-sectional study. *Gynecologic Oncology* 1994;**52**:14-9.

100. Chapman JD. A longitudinal study of sexuality and gynecologic health in abused women. *Journal of the American Osteopathic Association* 1989;**89**:619-24.

101. West CM, Williams LM, Siegel JA. Adult sexual revictimization among black women sexually abused in childhood: a prospective examination of serious consequences of abuse. *Child Maltreatment* 2000;**5**:49-57.

102. Tswana SA, Nystrom L, Moyo SR, Blomberg J, Tianani J, Nzara M *et al*. Hospital-based study of sexually transmitted diseases at Murewa rural district hospital, Zimbabwe 1991-1992. *Sexually Transmitted Diseases* 1995;**22**:1-6.

103. Stewart P, Slade P, Collett BJ, Cordle CJ, Stewart CR. Comparative study of pelvic and non-pelvic pain/the prevalence of chronic pelvic pain (multiple letters) [7]. *British Journal of Obstetrics & Gynaecology* 1998;**105**:1338-9.

104. Reed BD, Haefner HK, Punch MR, Roth RS, Gorenflo DW, Gillespie BW. Psychosocial and sexual functioning in women with vulvodynia and chronic pelvic pain. A comparative evaluation. *Journal of Reproductive Medicine* 2000;**45**:624-32.

105. Dimitrakov JD. A case of familial clustering of interstitial cystitis and chronic pelvic pain syndrome. *Urology* 2001;**58**:281vi-281viii.

106. Kennedy S. Primary dysmenorrhoea. *Lancet* 1997;**349**:1116.

107. Fox SD. Chronic pelvic pain in women. *Medicine & Health, Rhode Island* 2003;**86**:9-11.

108. Sanfilippo JS. Dysmenorrhea in adolescents. *Female Patient - Total Health Care for Women* 1993;**18**:29-33.

109. van Geelen JM. Prevalence studies in The Netherlands. *Round Table Series - Royal Society of Medicine* 1995;**Issue 38**.
