# Supplementary material for: WHO systematic review of prevalence of chronic pelvic pain: a neglected reproductive health morbidity
Source: BMC Public Health. 2006 Jul 6;6:177. doi: 10.1186/1471-2458-6-177 (PMC1550236; doi:10.1186/1471-2458-6-177)
Supplement: Additional file 1 — Tables 1, 2 and 3 of tables of characterstics of included studies on dysmenorrhoea, dyspareunia and noncyclical pelvic pain respectively [file 1471-2458-6-177-S1.doc]

Table 1: Table of included studies on dysmenorrhoea

| **Author, country, language, year of publication (reference list number)** | **Time period (mo)** | | **Age (yrs)** | **Population characteristics** | **Definition** | **Measurement tool ^+^** | **Response rate %** | | **Representative**  **(Y-yes, N-no)** | **Country resource*** | **Quality score (out of 5)** | **Cases** | Denominator | **Prevalence (95% CI)** | Notes | |
| --- | --- | --- | --- | --- | --- | --- | --- | --- | --- | --- | --- | --- | --- | --- | --- | --- |
| Community based studies | | | |  |  |  | |  |  |  |  |  |  |  |  | |
| Zondervan, UK, English, 2001(1) | 3 | | 14-49 | The women recruited in 1999 were registered with 85 general practices. One practice objected and hence their patients were excluded. Other exclusions were: mental illness (6), participation in the pilot study (5), 810 undelivered questionnaires | Pelvic pain with periods including irregular bleeding on oral pills or HRT | Semi structured self administered questionnaire (v) | 74 | | Y | 1 | 4 | 365 | 451 | 81  (77-84) |  | |
| Golding, USA, English, 1998(2) | Not stated | | 42.3;SE 0.5 | Los Angeles Epidemiologic Catchment Area study- 44.4% latina, 44.2% European American and 11.4% other groups, mean education11.5 years; Latin residents were interviewed in English or Spanish according to their preference | Excessively painful menstrual periods | Diagnostic interview schedule (v) | 86 | | Y | 1 | 4 | 391 | 1428 | 27.4  (25.1-29.8) |  | |
| Golding, USA, English, 1998(2) | Not stated | | Mean 43.9; SE 0.7 | NC-ECA sample was selected to represent adults in 2 mental health catchment areas in North Carolina, one consisting of Durham County, which is primarily urban and the other consisting of 4 contiguous rural counties | Excessively painful menstrual periods | Diagnostic interview schedule (v) | 79 | | Y | 1 | 4 | 479 | 1703 | 28.1  (26-30.3) |  | |
| Golding, USA, English, 1998(2) | Not stated | | Mean 46.1; SE 0.8 | In The national study of Health and Life Experiences of women; 84.3% were European American with mean education of 12.8 years | Excessively painful menstrual periods | Diagnostic interview schedule (v) | 91 | | Y | 1 | 4 | 430 | 963 | 44.6  (41.5-47.9) |  | |
| Flug, Switzerland, English, 1985(3) | 72 | | 12 to 21 | This study is part of the first Zurich Longitudinal Study of growth and development; the girls were followed for the first six years after menarche | Recurrent abdominal cramping at the time of menstrual flow for 6 months | Interview (v) | Not stated | | Y | 1 | 4 | 95 | 140 | 67.9  (59.4-75.5) |  | |
| Wilson, USA, English, 1989(4) | 18 | | 14-16 | All new female students entering a large independent coeducational secondary school answered a questionnaire about their menstrual history as part of health record in 1980 and 1981, follow up questionnaires and menstrual calendars were obtained on 3 separate occasions, mean age 15.45+/- 1.11 years, mean age at menarche 12.57+/-1.12 years, 72.5% with regular cycles, 250 boarders and 64 day students, 313 white and 14 black students | Dysmenorrhea divided into mild, moderate and severe type (current) | Questionnaire (v) | Not stated | | N | 1 | 4 | 185 | 327 | 56.6  (51-62) | 76//185 (41.1%) took prescription or non prescription medication for dysmenorrhea95 (31.7%) had mild 45(15%) had moderate and 17 (5.7%)had severe dysmenorrhea | |
| Andersch B, Sweden, English, 1982(5) | Not stated | | 19 | A sample of 1in 4 women born in 1962 (out of 2621), residing in Gothenburg city was obtained from the population register and these women were invited to participate in 1981; 3.1% used IUD and 10.1% women had been pregnant once. | Painful menstruation with severity defined by verbal multidimensional scoring system | Questionnaire (v) | 90.9 | | Y | 1 | 4 | 432 | 596 | 72.4  (68.7-76) | 15% suffered from dysmenorrhea that limited daily activity | |
| Barnard, USA, English, 2003(6) | 12 | | 28.5-43.1 | Participants were a nationally representative randomly selected sample of women veterans who had made at least one ambulatory visit to a Veterans Affairs facility between July 1 1994 and June 30, 1995 | Periods painful enough to interfere with usual schedule in the last 6 months | Structured questionnaire including SF 36 | 58.4 | | Y | 1 | 3 | 682 | 1736 | 39.2  (37-43.6) |  | |
| Gordley, USA, English, 2000(7) | Not stated | | 18-21 | Eligible military and civilian women employed at 10 US Air force bases with a mean age of 29.4 years and mean age at menarche 12.7 years agreed to participate in all aspects of the total study; 61.8% were Caucasian, 56.8% were either married or had a permanent partner and were well educated, 8.2% smoked | Need for bed rest or missing work due to menstrual pain ever | Questionnaire (nv) | 60.3 | | N | 1 | 3 | 53 | 170 | 31.2  (24.3-38.7) |  | |
| Holmlund U, Sweden, English, 1990(8) | Not stated | | 25-26 | All girls attending a classroom session in 1973 in 8th grade in the city of Norrkoping, a medium size Swedish city | Severe dysmenorrhea undefined | Attitude interest schedule, Cesarec Marke Personality Schedule (v) | 73 | | Y | 1 | 3 | 150 | 357 | 42  (36.8-47.3) | 23/150 (15%) had dysmenorrhea that limited their daily activity | |
| Mishra G, Australia, English, 2000(9) | 12 | | 18-23 | Study population were women aged 18-23 years who participated in the baseline survey of the Australian Longitudinal Study on Women's Health conducted in 1996, registered on the national Medicare database, oversampling from rural and remote areas, demographic and social background characteristics of respondents are broadly representative of Australian women in this age group, 41% had never taken OC pills | Severe period pain in the last 12 months | Self administered Questionnaire (v) | 42 | | Y | 1 | 3 | 5841 | 14762 | 39.6  (38.8-40.4) |  | |
| Sultan, France, English, 1999(10) | Not stated | | 14-18 | High school students in the Herault district of France completed questionnaire, 39% with family history of dysmenorrhea and 24% of the study subjects had a variety of psychological problems | Paroxysmal pain occurring before or during menstruation | Self administered Questionnaire (v) | Not stated | | Y | 1 | 3 | 902 | 4203 | 21  (20.3-22.8) | One third of the subjects with dysmenorrhea reported absenteeism from school due to pain | |
| Pedron-Nuevo, Mexico, Spanish, 1998(11) | Not stated | | 12 to 24 | Healthy single females from a school in the south of Mexico city, were given anonymous questionnaire; menarcheal age 8-19 (average 12.3 years) | Dysmenorrhea undefined (current) | Modified menstrual symptom questionnaire (v) | 100 | | N | 2 | 3 | 598 | 1066 | 56.1  (53.1-59.1) | Approximate rate | |
| Vicdan, Turkey, English, 1996(12) | Not stated | | 11 to18 | Between February-April 1992, 32 different schools with different socio-economic status in Ankara were chosen and 12 physicians administered the questionnaire to the school girls with average age 15.9 years; average menarcheal age was 13.28+/-1.09 yrs | Pain during menstruation | Questionnaire (nv) | Not stated | | Y | 2 | 3 | 10668 | 13665 | 78  (77.4-78.8) | 25.6% of the girls with dysmenorrhea missed school | |
| Ng, Singapore, English, 1992(13) | 12 | | 15-24 | From a sampling frame of all housing development board flats in Clementi town, a two stage cluster sampling was used to draw a random sample of 480 households; 30.3% Chinese, 28.8% Malay, 30.6% Indian; 36.8% married; 30.7% employed | One or more episodes of menstrual cramp or pain in the previous year not due to diagnosed gynaecological disease | Self administered questionnaire (nv) | 79.6 | | Y | 1 | 3 | 80 | 115 | 69.6  (60.3-77.8) |  | |
| Ng, Singapore, English, 1992(14) | 12 | | 25-51 | From a sampling frame of all housing development board flats in Clementi town, a two stage cluster sampling was used to draw a random sample of 480 households; 30.3% Chinese, 28.8% Malay, 30.6% Indian; 36.8% married; 30.7% employed | One or more episodes of menstrual cramp or pain in the previous year not due to diagnosed gynaecological disease | Self administered questionnaire | 79.6 | | Y | 1 | 3 | 133 | 200 | 66.5  (59.5-73) |  | |
| Cronje, South Africa, English, 1991(15) | Not stated | | 19-21 | Of the 10 boarding houses lodging female students on the campus of the university of the Orange Free state, Bloemfontein, 4 were randomly selected, all second year students were requested to complete a questionnaire; all were Caucasian and Afrikaans speaking who had attained menarche between 11 & 16 years | Dysmenorrhea undefined | Questionnaire (nv) | 78.5 | | N | 2 | 3 | 92 | 102 | 90  (82.7-95.2) | 37/102 (36%) experienced severe pain | |
| Robinson J, USA, English, 1992(16) | 6 | | 10 to 18 | The data are from a three wave panel study of <18 unmarried females, non pregnant, adolescents wishing contraception who attended a Planned Parenthood clinic in Baltimore during 1988, 77% black inner city population, 58% had been pregnant and 12% had given birth | Cramps during menstrual period in 3-6 months | Telephone interview (v) | 89 | | N | 1 | 3 | 245 | 308 | 79.6  (74.6-83.9) | 18.2% reported severe dysmenorrhea on Andersch-Milsolm scale | |
| Sundell, Sweden, English, 1990(17) | 12 | | 24 | 19 year old women resident in Gothemberg born in 1962 were studied in 1981; 596 women were surveyed in 1986, 1 woman had died and 6 emigrated with 453 women still resident in Gothenburg | Painful menstruation with severity defined by verbal multidimensional scoring system | Questionnaire (v) | 83 | | Y | 1 | 3 | 308 | 489 | 63  (58.5-67.3) | 72% in the same women 5 years earlier | |
| Bang RA, India, English, 1989(18) | Not stated | | Mean 32.1 | Survey conducted in Gadchiroli district of Maharashtra, India in two villages; all females over age 13 years were invited to participate; 25% of the non participating sample surveyed and found to be similar to study sample | Undefined | Interview (nv) | 59 | | Y | 2 | 3 | 269 | 468 | 57.5  (52.9-62) |  | |
| Fisher, USA, English, 1989(19) | 8 | | 16-18 | The survey was administered between Sept.85-May 86, to female patients and friends (mean age 17.6 years) in the waiting room of the Five Towns Adolescent Health Service, a general health facility for teenagers located in a middle to upper-middle class suburb of New York City between Sep 1985-May 1986; predominantly white (86%), most were catholic (55%) or Jewish (32%), 59% were in high school | Mild, moderate, severe dysmenorrhea in the last cycle | Premenstrual Assessment form (v) | 100 | | Y | 1 | 3 | 180 | 207 | 87  (81.6-91.2) | 25% mild, 39% moderate and 23% severe | |
| Teperi, Finland, English, 1989(20) | 12 | | 12 to 18 | All (3370) girls born in consecutive days in July 1964, 66, 68 and 70 derived from National Population registry | Menstrual pain-mild, moderate or sever needing medication or leading to absenteeism from work or school | Questionnaire (v) | 87 | | Y | 1 | 3 | 1876 | 2932 | 64  (62.2-65.7) |  | |
| Scambler, UK, English, 1985(21) | Not stated | | 15-44 | Women registered with two health centres serving a new estate on the eastern fringes of London | Pain before or during a period in 6 weeks | Moos Menstrual Distress Questionnaire (v) | 62.7 | | Y | 1 | 3 | 24 | 79 | 30.4  (20.5-41.4) |  | |
| Woods NF, USA,English, 1982(22) | 5 | | 18-35 | A population of non pregnant women residing in five neighbourhoods of a large south-eastern city with varied racial composition and SE status was chosen in 1979 | Dysmenorrhea undefined | Moos Menstrual Distress Questionnaire (v) | 74 | | Y | 1 | 3 | 30 | 179 | 16.8  (11.6-23.1) |  | |
| Klein J, USA, English, 1981(23) | Not stated | | 12 to 17 | Data from the national health examination survey cycle III collected between 1966-1970 by the National Centre for Health Statistics | Abdominal pain or discomfort in relation to menses | Questionnaire (v) | 100% | | Y | 1 | 3 | 1611 | 2699 | 59.7  (57.8-61.5) | This includes occasional mild pain; 44.17% have pain sometimes or always | |
| Sehgal, India, English, 1971(24) | Not stated | | 16-25 | College girls of Banaras Hindu university residing in 3 hostels inside the university campus were interviewed; 82.3% were from urban areas and 91% were Hindus; 79.5% were below 21 years of age and 5.2% were married | Dysmenorrhea- mild if girls attended to daily work without analgesics, moderate if girls could attend classes with analgesics and severe if they had to miss classes due to discomfort | Proforma (nv) | 83.8 | | N | 2 | 3 | 172 | 358 | 48  (42.8-53.4) |  | |
| Weissman, USA, English, 2004(25) | 12 | | 19-45 | Participants were nurses who graduated between 1963-1984. They were surveyed twice at an interval of six years (1985, 1991) regarding menstrual cycle characteristics. In 1985 80% of respondents were over 25 years old and 60% were parous and all gave informed consent. 14% of women who reported endometriosis, PID or uterine fibroids in either 1985 or 1991 were excluded | Menstrual cramps graded as none, minimal (can work, somewhat uncomfortable), moderate (can work but quite uncomfortable) and severe (miss work, have to be in bed) | Self administered questionnaire (nv) | 73 | | N | 1 | 2 | 301 | 404 | 74  (70-78.7) |  | |
| Hirata, Japan, Japanese, 2002(26) | 1 | | 18 to 21 | Of the college students, 34.8% of them belonged to the underweight group (BMI < 19.8), 53.8% to the normal group (19.8 < or = BMI < 24.2) and 11.4% were overweight (BMI > or = 24.2) | Menstrual pain regularly, sometimes or seldom | Questionnaire (nv) | 84 | | Y | 1 | 2 | 1876 | 2282 | 82.2  (80.6-83.8) |  | |
| Walraven, Gambia, English, 2002(27) | Not stated | | 15-54 | 20 villages were randomly selected for inclusion in the study, no specific exclusion criteria; 72% response rate15-54 year old women | Pain with menstrual periods that prevents normal activities in the last 3 months | Semi-structured interviews | 72.3 | | Y | 3 | 2 | 85 | 607 | 14  (11.3-17) |  | |
| Larroy, Spain, Spanish 2001(28) | 1 | | 13-52 | Women from urban and rural areas of Madrid were recruited from schools, universities, clinics, women's association, community centers, 0.4% were uneducated, 23.4% studied upto primary school level, 58.3% went to secondary school and 17.9% were university educated, women who used OC or IUD or had gynaecological disease excluded | Pain during menstruation without presence of any organic disease | Larroy's questionnaire (v) | Not stated | | Y | 1 | 2 | 859 | 1387 | 61.9  (59.3-64.5) |  | |
| Di Wu et al, China, English, 2000(29) | 12 | | 30-40 | Subjects were non smokers, non drinkers, no physician diagnosed organic diseases; blood sample was taken for genotyping | Pelvic or lower abdominal pain associated with menstruation - recurrent/occasional and mild/moderate/severe | Self administered Questionnaire (nv) | 87 | | N | 2 | 2 | 129 | 435 | 29.7  (25.4-34.2) |  | |
| Gonzalez, Chile, Spanish, 1999(30) | 1 month | | 16-25 | Cross sectional study of students of the Faculty of Education of the Pontificia Catholic University of Chile | 95 | Dysmenorrhoea unspecified | Self administered questionnaire (nv) | | Y | 2 | 2 | 207 | 242 | 85.5 |  | |
| Kritz-Silverstein, USA, English, 1999(31) | 11 | | 18-49 | All women serving aboard navy ships were eligible; the study was part of the Women Aboard Navy ships Comprehensive Health and Readiness Research Project; mean age 26+/-6.1 years, 58% white, 32% black and 10% were other racial groups | Menstrual cramps requiring medication or time off work | Self administered Questionnaire (v) | 63.1 | | N | 1 | 2 | 704 | 2657 | 26.5  (24.8-28.2) |  | |
| Campbell, USA, English, 1997(32) | 3 | | 14-21 | Mean age 16.26 years, a convenience sample that represented 96% of the female high school students attending a public high school in Halifax, Nova Scotia, were approached during class; 85% were English Canadian, 5% French Canadian; 0.7% of the participants were SE class I and 30% were class V | Any discomfort in association with the onset of menstruation that was not the result of an organic abnormality such PID in the last 3 cycles | Menstrual Distress Questionnaire (v) | 80 | | N | 1 | 2 | 269 | 289 | 93  (89.5-95.7) |  | |
| Elahi, Pakistan, English, 1997(33) | Not stated | | 12 to 18 | Muslim girls from Multan city | Dysmenorrhea defined by severity of signs and symptoms of pain during menses and the ability of the respondents to perform daily activity | Questionnaire (nv) | Not stated | | N | 2 | 2 | 529 | 696 | 76  (72.7-79.1) |  | |
| Harlow, USA, English, 1996(34) | 12 | | 17-19 | In 1985 nulliparous women not using OC pills or IUD and entering a local state university enrolled in a one year menstrual diary study, median age at menarche 13 years, 43% with long cycles, 20% smokers | Pain events associated with menstrual period in 12 months | Menstrual diary (v) | 84 | | N | 1 | 2 | 1000 | 1396 | 71.6  (69.2-74) | Value for observed bleeds, 13% reported having severe pain more than half the time | |
| Mathias, USA, English, 1996(35) | 6 | | 18-50 | In April-May 1994, the Gallup organization telephoned 17927 households by random digit dialing to identify women between 18-50 years; in women with more than one eligible woman, a computer generated algorithm identified the woman to be interviewed; demographic profile of the survey participants with CPP was similar to women with no CPP in terms of age, race, ethnicity, education (i.e. mean age 35.7+/-8.6 versus 36.7+/-8.6), 37% of women in both groups had less than a high school education, 10 vs. 11% had an advanced college degree, 86 vs. 82% were white, 7versus 9% were African American, 4 vs. 5% were latino | Pelvic pain with periods including irregular bleeding on oral pills or HRT in the last 6 months | Telephone interview (nv) | 88.8 | | Y | 1 | 2 | 90 | 5325 | 1.7  (1.4-2.1) | 25% has seen some health care provider in the last 3 months; total indirect cost of CPP due to time lost from work $555.3 million and estimated direct medical costs for US population of 18-50 yr old women are $881.5 million per year. | |
| Skierska, Poland, Polish, 1996(36) | Not stated | | 15-36 | Mean age 22.04+/-4.6years, cohort from Warshaw, 5.3% with university education, 48.6% with secondary education, average height 165.5 cm, average BMI 20.9+/-2.5; mean age at menarche 12.9 years | Strong crampy pain in the suprapubic region or lower back sometimes associated with nausea or headache just before or with menses needing analgesia | Standard questionnaire- anonymous interview by trained interviewers (v) | 95 | | Y | 1 | 2 | 415 | 734 | 56.3  (52.9-60.2) |  | |
| Onatra, Columbia, Spanish, 1994(37) | 3 | | 10 to 19 | Group A includes 481 adolescents of the urban area of Santafe de Bogota, attending private school; Group B includes 285 adolescents attending state school and Group C includes 30 indigenous adolescents of the Tukano tribe from the rural area of Mitu, capital of Vaupes Adolescents filled up questionnaire; no other details provided | Pain during menstruation divided into mild, moderate and severe by WHO typology | WHO questionnaire (v) | 95 | | Y | 1 | 2 | 484 | 796 | 60.8  (57.3-64.2) | Dysmenorrhea is less frequent and of lesser intensity within the indigenous group than within the urban one | |
| Lee, USA, English, 1991(38) | 12 | | 21-50 | Data from a larger study of women working as nurses in seven hospitals on the west coast. Excluded subjects were over 50 years of age, not menstruating, or had been pregnant or lactating during the six months prior to completing the questionnaire; each worked 32-55 hours per week, 86.1% Caucasian, 58.3% married or partnered, 53.5% had been pregnant and 65.8% had no living children at home | Cramps just before or at the start of menstrual period over last 6 months | Self administered questionnaire (v) | 69 | | N | 1 | 2 | 321 | 594 | 54  (49.9-58.1) | 30.7% experienced cramps every month; 34.2% rarely | |
| Odujinrin, Nigeria, English, 1991(39) | Not stated | | 10 to 18 | 10-18 years old secondary school girls were randomly selected and surveyed, 889/950 had attained menarche, 35.8% had irregular cycles and 78.6% had flow between 3-5 days | Pain during menstruation graded on a multidimensional verbal scoring system | Self administered questionnaire (nv) | Not stated | | N | 2 | 2 | 638 | 889 | 71.7  (68.7-74.7) | 350 (39.40%) had mild, 197 (22.2%) had moderate and 91(10.2%) had severe dysmenorrhea | |
| Mijanovic, Macedonia, Roman, 1990(40) | Not stated | | 10 to 19 | Randomly chosen adolescent females, aged 10 to 19 years, were questioned about their (painful or painless) menstruation | Painful menstruation | Examination (v) | Not stated | | Y | 1 | 2 | 565 | 1068 | 52.9  (49.9-55.9) |  | |
| Thomas, Nigeria, English, 1990(41) | Not stated | | 15-24 | The female students of Obafemi Awolow University, Nigeria, with mean age 19.8+/-2.4, median age at menarche 13.5 years, 64.3% had 26-28 menstrual cycle and 26.8% participated in competitive sporting activities | Presence of pain during menstruation | Questionnaire (nv) | Not stated | | Y | 2 | 2 | 555 | 768 | 72.3  (69-75.4) |  | |
| Busch, USA, English, 1988(42) | Not stated | | 18-39 | Nursing students were recruited from undergraduate and graduate classes at a university and a community college; women older than 39 years or those who did not list their age or had hysterectomy or had missing data were excluded; average age 25.1 years, 62% single, 26% married, 38% protestants, 39% catholic, 13% Jewish; 17% used oral contraceptives; 34% used other contraceptive methods | Severe cramps during menses | Questionnaire (v) | 61.8 | | N | 1 | 2 | 141 | 503 | 28  (24.1-32.2) | 26.8% report seeking medical treatment | |
| Johnson J, USA, English, 1988(43) | Not stated | | 14-18 | Students from two separate school settings in or near a metropolitan Midwestern community were surveyed; mean age 15.8 years, 6.6% social class I and mean age at menarche was 12.5 years | Painful menstruation (current) | Self administered questionnaire (nv) | 100 | | Y | 1 | 2 | 136 | 182 | 74.7  (67.8-80.9) | 21/136 (15.5%) had used a prescription medication | |
| Pullon, New Zealand, English, 1988(44) | 1 | | 16-54 | Of women surveyed from Wellington region in 1 week in June 1985,80% of the women who were currently menstruating were included in the analysis; (30 GP surgeries randomly selected and 76% of the surgeries approached participated) | Pain with periods | Self administered questionnaire (nv) | 96 | | Y | 1 | 2 | 772 | 1456 | 53  (50.4-55.6) | 176 women (12%) severe enough to take time off work or school; 176/1546 (12%) reported pain lasting 3 days or more and 520/1456 (36%) reported pain lasting for 2 or more days | |
| Silberg, Australia, English, 1987(45) | 16 | | 18-53 | Between November 1980-March 1982, 1233 pairs monozygotic and 751 pairs dizygotic twins were surveyed and repeatability was tested after 3 months; both twins were menstruating regularly and neither was pregnant at the time of completing questionnaire; mean age 29+/-8 years | Moderate or severe pain during period severe enough to limit normal activity or to require medical treatment | Questionnaire (v) | 64 | | Y | 1 | 2 | 1296 | 2492 | 52  (50-54) |  | |
| Whorwell, UK, English, 1986(46) | Not stated | | 18-54 | Age, social class matched controls for patients with IBS recruited from staff registers of local commerce and industry after completing a screening questionnaire to exclude subjects with IBS who were about 11% of the group | Dysmenorrhea undefined | Interview-Questionnaire (nv) | Not stated | | N | 1 | 2 | 42 | 90 | 46.7 (36.1-51.5) |  | |
| Mergler, Canada, English, 1985(47) | Not stated | | 15-35 | Part of the health study undertaken at the request of the employees of 8 Quebec poultry slaughterhouses- women working in 8 poultry houses and controls were full time housewives married to male slaughterhouse workers who were not pregnant, not menopausal, not amenorrhoeic or not undergone hysterectomy; 75% of the workers and 53.5% of the housewives used OC pills, 83.8% and 61% respectively were nulliparous | Lower abdominal pain during last period | Questionnaire (nv) | 100 | | Y | 1 | 2 | 211 | 318 | 66.4  (60.9-71.5) |  | |
| Svanberg L, Sweden, English, 1981(48) | Not stated | | 10 to 19 | The present study was carried out on 502 girls in primary and secondary schools in Malmo, Sweden with mean age 15 years; 66% of the subjects reported that they had the menarche between 12 and 13 years of age, mean duration of menstrual flow was 4-5 days, 72% had dysmenorrhea within the 1st year of menarche | Dysmenorrhea undefined | Questionnaire (nv) | 100% | | Y | 1 | 2 | 216 | 502 | 43  (38.6-47.5) | Occasional or consistent dysmenorrhea | |
| Webster, USA, English, 1979(49) | Not stated | | <25 | 58 students and 217 young working women were instructed to answer each item of the questionnaire but not to spend too much time on any single item; 102 were taking Oral Contraceptives, 17 had Intrauterine devices | Spasmodic and congestive dysmenorrhea | Menstrual distress questionnaire (v) | Not stated | | Y | 1 | 2 | 221 | 275 | 80.4  (75.2-84.9) |  | |
| Wood, Australia, English, 1979(50) | Not stated | | 15-53 | Data from menstruating women attending Shepherd foundation for comprehensive health check | Menstrual pain undefined | Menstrual distress questionnaire (v) | Not stated | | N | 1 | 2 | 1856 | 2343 | 79.2  (77.5-80.8) | Includes occasional mild pain; 44.17% have pain sometimes or always | |
| Theano, Spain, English, 1968(51) | Not stated | | 17-32 | Unmarried girls at college, mean age 22 years, resident in college, average age of the menarche was 13.3 years | Mild, moderate, severe dysmenorrhea | Questionnaire (v) | Not stated | | N | 1 | 2 | 56 | 60 | 93.3  (83.8-98.1) | 28.3% mild, 36.7% moderate and 28.3% severe | |
| Hirt,USA, English, 1967(52) | Not stated | | <25 | Freshman student nurses at a large metropolitan hospital, relatively homogenous in age and socio-economic status were surveyed; all received physical examination | Mild moderate or severe pain before or during menstruation | Semi-objective criteria for teenage dysmenorrhea and 16 personality factor questionnaire (v) | 100 | | N | 1 | 2 | 81 | 105 | 77  (67.9-84.7) |  | |
| Widholm, Finland, English, 1967(53) | Not stated | | 11 to 21 | Series of secondary school pupils who visited Folkhalsan teenagers' clinic with various symptoms including leucorrhoea, premenstrual tension, fatigue, headache etc. were included; mean menarcheal age 13.2 yearsControl series mean age 15.7 years from two school classes who underwent same examination; mean age at menarche 13 years | Dysmenorrhea undefined | Not specified | Not stated | | Y | 1 | 2 | 190 | 514 | 37  (32.8-41.3) | 38.1% for hospital and 24.4% for community | |
| Kessel, UK, English, 1963(54) | Not stated | | 17-32 | Details not available | Mild, moderate, severe dysmenorrhea | Questionnaire (v) | Not stated | | N | 1 | 2 | 424 | 500 | 84.8  (81.3-87.8) | 32.4% mild, 36.5% moderate and 15.9% severe | |
| Golub, USA English, 1958(55) | Not stated | | 12 to 18 | 16183 girls in 16 Philadelphia public high schools which represents over 90% of the girls attending these schools, percentage of Negro students varied from school to school, ranging from 0 to 80.5% | Lower abdominal cramping pain at the time of menstrual flow | Interview (nv) | Not stated | | Y | 1 | 2 | 10938 | 16183 | 67.6  (66.9-68.3) | Any dysmenorrhea | |
| Pawloski, Poland, English, 2004(56) | 3 | | 18-45 | Between February 1999- December 2000, data was collected on entire community of one Mayan village; women over age 45 and those who had delivered in the last 9 months were excluded; 79 women were of Mayan ancestry, 67 were half Mayan and 31 women had no Mayan ancestry | Pain during menses sometimes or always | Interview (nv) | 61.5 | | Y | 2 | 1 | 50 | 177 | 28  (21.7-35.5) |  | |
| Schmidt, Brazil, Portuguese, 2002(57) | 6 | | 12 to 19 | School girls from Sao Leopoldo, 92/100 eligible as menstruating | Discomfort during periods in the last 6 months | Self administered questionnaire (nv) | 90.9 | | Y | 2 | 1 | 64 | 92 | 69.6  (59.1-78.7) |  | |
| Dusek, Croatia, English, 2001(58) | Not stated | | 15-21 | Mean age of athletes 17.9+/- 2.1 non-athletes 17+/- 1.1The study involved female athletes from 3 Zagreb sports clubs as well as ballet dancers. The control group comprised of 3rd and 4th grade high school girls from the school of textile design in Zagreb who did not take part in any sport activity. None of the girls used Oral Contraceptives. | Painful menstruation (current) | Questionnaire (nv) | Not stated | | N | 1 | 1 | 76 | 163 | 57.3  (38.8-54.6) |  | |
| Balbi C, 2000, English, Italy(59) | Not stated | | 14-21 | Students from a professional institute in Naples were included | Pain accompanying menstruation localized in the abdominal inferior quadrants and radiates to inner thigh | Interview (v) | Not stated | | N | 1 | 1 | 201 | 356 | 56.5  (51.1-61.7) |  | |
| Banikarim, USA, English, 2000(60) | Not stated | | 13-18 | Hispanic female adolescents, in grades 9 through 12, completed a 31-item questionnaire about the presence, duration, severity, treatment, and limitations of dysmenorrhea at a local urban high school. | Painful menstruation during the previous 3 months and categorised as mild, moderate and severe | Self administered Questionnaire | Not stated | | N | 1 | 1 | 600 | 706 | 85  (82-87.1) |  | |
| Hillen T, Australia, English, 1999(61) | Not stated | | 15-17 | 47.4% respondents from private school, 34.3% from the state high school in higher SES area and 18.3% from lower SES area | Any type of pain or discomfort associated with menstrual periods including cramps, nausea and headaches | Self administered anonymous Questionnaire(nv) | 99 | | Y | 1 | 1 | 309 | 384 | 80  (76.1-84.3) |  | |
| Okonofua, Nigeria, English, 1990(62) | Not stated | | 16-24 | Women athletes participating in the bi-annual interuniversity games held at university of Abadan, Nigeria in April 1986 were approached; subjects' age weight, height ranged between 16-28 years, 42 and 83 kg and 51 and 75 inches respectively; non-contact sports athletes were younger, lighter and leaner than contact sports athletes | Undefined | Questionnaire (v) | Not stated | | N | 2 | 1 | 47 | 133 | 35.3  (27.3-44.1) |  | |
| Vishwanathan, USA, English, 2000(63) | Not stated | | 40-44 | Part of detailed baseline study of the health status of college alumnae in 1996; half of whom were college athletes and half non-athletic classmates; 516 of these were eligible for inclusion in this study as they were aged 15-44 years and had had at least one pregnancy at the time of reporting; 460 of these were not sterilized and 56 were sterilized | Menstrual pain (current) | Self administered Questionnaire (nv) | Not stated | | N | 1 | 1 | 122 | 516 | 23.6  (20-27.6) |  | |
| Thongkrajai, Thailand, English, 1999(64) | Not stated | | 15-55 | Healthy 15-55 years old women from 4 villages in Nampong district, Khon Kaen Province were recruited via a mobile health clinic to collect data, physical examination and specimen collection to screen for STD, 93.4% married, 85% farmers, 94.5% finished primary school and 20% subjects had abortion | Dysmenorrhea undefined over last 3 months | Interview (nv) | Not stated | | Y | 2 | 1 | 284 | 634 | 44.8  (40.9-48.8) |  | |
| Jarrett, USA, English, 1995(65) | 2 | | 21-44 | Participants were screened to determine that they were currently menstruating regularly without using birth control pills or an IUD and had no history of gynaecological or gastrointestinal surgery/pathology or renal pathology; 83% were white and 64% had college degree. | Menstrual cramps for 3 days in 2 cycle | Health questionnaire menstrual diary (v) | 78 | | N | 1 | 1 | 27 | 61 | 44.3  (31.5-57.3) |  | |
| Messing, Canada, English, 1993(66) | 12 | | 24-41 | Women working in 17 poultry slaughterhouses and 6 canning factories in western France employed on 1/1/87 were randomly selected; 87 women eliminated due to menopause, 62 were pregnant or reported having been pregnant in the last 2 years and thus might not have resumed normal menstruation, 2 had hysterectomies and in 4 the data was incomplete; data adjusted for age, contraceptive method and tobacco use, 249/720 women were on OC pills | Pain in the abdomen or lower back during menstrual periods or during the days preceding the period over last 12 months | Questionnaire (nv) | 98.5 | | N | 1 | 1 | 685 | 978 | 69  (66-71.9) | 58% women had pain both preceding and during period | |
| Izzo, Italy, English, 1991(67) | Not stated | | 16-23 | Women from Campania belonging to the same race and socio-cultural background; one group practiced sport in their adolescent period and the other took part in sport occasionally | Pain which precedes by a few hours or accompanies menstrual flow | Self administered questionnaire(nv) | Not stated | | Y | 1 | 1 | 441 | 764 | 57.7  (54.1-61.3) | 129/169 (76.3%) women did not practise sports regularly had dysmenorrhea | |
| Bukman, Holland, English, 1990(68) | Not stated | | 21-42 | Volunteers were obtained from a general practitioner, had no wish to become pregnant and were not using oral contraceptives or an intrauterine device | Painful menstrual flow or pelvic pain during menstruation | Questionnaire (v) | 52.7 | | N | 1 | 1 | 33 | 39 | 84.6  (69.5-94.1) | Healthy volunteers | |
| Clarvit, USA, English, 1988(69) | Not stated | | 21-46 | Questionnaire was placed in the school mailboxes of every female medical student at Albert Einstein College of Medicine in Bronx, New York along with an introductory letter explaining the purpose of the study and that all responses would be anonymous | Painful menstrual cramps before and during medical school | Self administered questionnaire (nv) | 63 | | N | 1 | 1 | 116 | 159 | 73  (65.3-79.7) | 71.1% prevalence before medical school | |
| Gruber, USA, English, 1987(70) | 1 | | 17-24 | Women recruited from Introductory Psychology classes at a Midwestern university; mean age 18.5 years, average menstrual flow 5.2 days | Dysmenorrhea according the MSQ-R scale in the last cycle | Menstrual symptom questionnaire-revised, menstrual activity questionnaire (v) | Not stated | | N | 1 | 1 | 207 | 293 | 70.6  (65.1-75.8) |  | |
| Iglesias, Mexico, English, 1980(71) | Not stated | | Not stated | Medical service of the company polled at random airline stewardesses at the time they applied for assistance of a variety of clinical problems, personnel using contraception or suffering from obvious general or gynaecological problems were excluded | Dysmenorrhea undefined | Interview (nv) | Not stated | | N | 2 | 1 | 34 | 200 | 17  (12.1-22.9) |  | |
| Widholm, Finland, English, 1979(72) | Not stated | | 13-20 | Control series of case-control study with mean age 15.7 years from two school classes who underwent same examination; mean age at menarche 13 years-study done in 1967. In order to obtain a picture of the need for drugs for pain relief and absenteeism, this second similar study was done | Dysmenorrhea undefined | Questionnaire (nv) | Not stated | | N | 1 | 1 | 35 | 97 | 36.1  (26.6-46.5) | % Calculated from responders; 3.2% consulted doctor; 9.3% absent from schools due to pain. | |
| Malina, USA,English, 1978(73) | Not stated | | <25 | 21subjects from university of Texas in Austin; college athletes, nonatheletes and Olympic basketball athletes; all except one white; 27 non-athletes were volunteers from physical instruction classes who had not participated in interscholastic sports at any school level | Dysmenorrhea undefined | Interview (nv) | Not stated | | N | 1 | 1 | 7 | 27 | 25.9  (11.1-46.3) | Non athletes | |
| Sheldrake, UK, English,1976 (74) | Not stated | | 14-18 | Survey was carried out on the student population registered for study at Edinburgh university for the academic year 1974-75, 756/3298 were taking oral contraceptive pill, mean cycle length 29.1 days | Stomach ache during period | Questionnaire (nv) | 88.4 | | N | 1 | 1 | 1458 | 3298 | 44.2  (42.5-45.9) | 40.8% for women taking Oral Contraceptives and 32% for women on Oral Contraceptives for over 2 years | |
| Bergsjo P, Norway, English, 1975(75) | Not stated | | 19-50 | Subjects examined were employed in an electrical/technical company in Oslo and in textile industry in Bergen | Pain shortly before or during menstruation during the last 3 months | Questionnaire (v) | Not stated | | N | 1 | 1 | 28 | 54 | 52  (37.8-65.7.) | 23% of the whole sample had contacted Dr for dysmenorrhea; 31% absent from work; 28% had to stay in bed | |
| Widholm and Kantero, Finland, English, 1971(76) | Not stated | | 10 to 20 | No information available | Dysmenorrhea undefined | Questionnaire (nv) | Not stated | | N | 1 | 1 | 5155 | 5485 | 94  (93.3-94.6) |  | |
| Clow, UK, English, 1924(77) | 60 | | 12 to 22 | Interview of healthy girls was taken by the author | Slight, sub acute and disabling dysmenorrhea | Interview (v) | Not stated | | N | 1 | 1 | 451 | 2050 | 22  (20.2-23.9) | 4% required bed rest during pain | |
| Rojas, Colombia, Spanish, 1997(78) | Not stated | | 12 to 23 | Cross sectional study of adolescents in a school; 7% incapicitated for 2/3 days and 12% for a day by pain during menses | Not stated | Pain during menses mild, moderate or intense | Visual analogue scale | | N | 2 | 1 |  |  | 83.2 |  | |
| Shye, Israel, English, 1991(79) | Not stated | | 16 to 21 | Cohort of adolescents were studied for premenstrual symptoms and menstrual pain; Girls of Asian or African ethnic origin reported consistently higher perimenstrual distress than those of Israeli or Western origin | Painful menses in the past | Questionnaire by interview (nv) | Not stated | | Y | 2 | 1 | 381 | 545 | 70  (65.9-73.7) | 25% experienced pain severe enough to interfere with daily activities in the past 3 cycles | |
| Yang JM, China, English, 2002 (80) | 6 | | 15-44 | A epidemiological study was conducted on 296 non pregnant female workers exposed to mercury vapor and 394 female workers from food processing plants working for at least 1 year; women using Oral Contraceptives, intrauterine device or steroid hormones were excluded | Serious menstrual pain that affected work or required medication to relieve symptom before or during menstrual cycle | Self administered questionnaire (nv) | Not stated | | N | 2 | 0 | 42 | 394 | 10.7  (7.8-14.1) |  | |
| Nunez, Venezuela, Spanish, 1991(81) | 3 months | | 16-30 | 20,20% of the total of the female population of 3787 students during the period between January and March of 1988 were surveyed | Not stated | Pain during menses | Not specified | | N | 2 | 0 | 536 | 765 | 70 |  | |
| Pena Nina, Dominican Republic, Spanish, 1993(82) | Not stated | | Not stated | laborers of business of zone franca and banking institution of the city of Holy Sunday were surveyed. | Not stated | Dysmenorrhoea unspecified | Not clear | | N | 2 | 0 |  |  | 55.3 |  | |
| Montero, Spain, English, 1996(83) | 12 | | 14-20 | Girls attending secondary schools in the municipality of Alcobendas, on the outskirts of Madrid were interviewed | Menstrual pain over the last 12 months | Questionnaire by interview (nv) | Not stated | | Y | 1 | 0 | 670 | 1146 | 58.5  (55.5-61.3) | Approximate rate | |
| Barros, Brazil, Spanish, 1987(84) | Not stated | | 18-24 | Organizaçäo Santamarense de Educaçäo e Cultura University students were interviewed on voluntary participation | Not stated | Light, moderate and severe dysmenorrhoea | Questionnaire-interview (nv) | | N | 2 | 0 | 768 | 1006 | 76.3 | 40,4% light form; 27,8% form moderate and 8,2% severe form | |
| Lemasters, USA, English, 1985 (85) | Not stated | | Not stated | Workers in Styrene industry, 123 in low exposure job, 51 in high-exposure job; 449 unexposed women-117 in unexposed jobs in styrene industry, 176 had jobs outside the plastic industry and 156 were unemployed | Dysmenorrhea undefined (current | Questionnaire (nv) | Not stated | | N | 1 | 0 | 86 | 615 | 14  (11.3-17) | 25/156 (16%) dysmenorrhea in unemployed | |
| Toriola, Nigeria, English, 1986 (86) | 4 | | 18-26 | Nov. 1984-Feb.1985, mean age 24.1 years, athletes in various sports with at least 3 years experience of national level competitive sports; non-athletes between 19-28 years with a mean age of 24.6 years were selected from women attending secondary schools, colleges or universities in the same localities where the athletes trained; 2and 3% of athletes and non athletes were excluded because they were unwilling to provide information on menarcheal age and other menstrual characteristics, data on 8.79% of athletes and nonatheletes was excluded because in incompleteness, use of OCs, pregnancy or breastfeeding or medical treatment that interfered with normal endocrine function | Dysmenorrhea undefined (current) | Questionnaire (nv) | Not stated | | N | 2 | 0 | 175 | 1081 | 16.2  (14-18.5) |  | |
| Sogbamu, Nigeria, English, 1979(87) | Not stated | | 12 to18 | Survey sample included residential pupils from one of the secondary schools in Ondo, Nigeria | Dysmenorrhea undefined | Questionnaire (nv) | 100 | | N | 2 | 0 | 38 | 119 | 31.9  (23.7-41) | 38% of those with dysmenorrhea had analgesics prescribed by the school nurse | |
| Khatri, India, English, 1978 (88) | Not stated | | 20-40 | Parous women residing in sector 10,11, 15 and 22, educated, married and not using IUD, OCs or tubal ligation for contraception were chosen if they had re-established menses after the last delivery. Women who gave history of recent dilatation and curettage were also excluded. Mean age of menarche 14.5 years, average cycle length 28-33 days with average of 5 days of bleeding | Dysmenorrhea undefined | Interview (nv) | Not stated | | N | 2 | 0 | 291 | 1000 | 29.1  (29-29.4) | 16.9% was mainly of congestive type; 9% severe and 60.1% mild dysmenorrhea.19.6% of women with dysmenorrhea took analgesics, 14.3% bed rest and 1% took hormones | |
| Timonen, Finland, English,1973 (89) | Not stated | | 18-27 | Sample was drawn by sending a questionnaire to one-tenth of the female students at the university of Helsinki and to female students at the Institute of Physical Education, Jyvaskyla; 65% were 21-24 years old | Menstrual pelvic pain | Questionnaire (nv) | Not stated | | Y | 1 | 0 | 116 | 234 | 49.6  (43-56.2) |  | |
| **Hospital/community clinic based studies** | | | | | | | | | | | | | | | |  |
| Rulin M, USA, English, 1993 (90) | | 36 | mean 28 | Low income, ethnically and regionally diverse women from three participating institutions; women taking OC (147) in the control group were excluded from analysis; modified Pomeroy technique used for puerperal sterilisation; interval sterilisation was done by falope bands or bipolar electrocautery, control group -women who had never been sterilised and a subset of this group not using OCS with mean age of 27 years | Bad menstrual cramps over 3-4.5 years | Questionnaire+ interview (v) | 78 | | N | 1 | 3 | 131 | 819 | 16  (13.5-18.7) | Values for women undergoing sterilization- 16% (80/500) 95% CI- 12.9-19.511.6% preoperatively, 15.9% 8-11 months later | |
| El-Defrawi, Egypt, English, 2001(91) | | Not stated | Not stated | Patients of maternal and childhood centre, Islamia were gynecologically examined and psychosexual interview was taken by interviewers who were blind to the woman's circumcision status, 200 circumcised and 50 were uncircumcised | Dysmenorrhea undefined | Semistructured psychiatric interview based on Arabic version of the sexual behavior Assessment Schedule-adult (SEBAS-A)(v) | 87 | | N | 2 | 1 | 161 | 200 | 80.5  (74.3-85.8) | Circumcised women | |
| Gurel, Turkey, English, 1998 (92) | | Not stated | 18-56 | Women with various gynaecological complaints admitted in the dept. were included in the study. All were married and 14 were nulliparous. Those having previous pelvic surgery including caesarean section, those with pelvic mass, uterine anomaly, pregnancy or intrauterine device were excluded | Primary dysmenorrhea influencing daily life or requiring analgesia | Questionnaire (nv) | Not stated | | N | 2 | 1 | 134 | 235 | 57  (50.4-63.4) |  | |
| Jamieson DJ, USA, English, 1996 (93) | | 3 | 18-45 | Two O&G and three family medicine practices in North Carolina were surveyed between February-April 1993, on 5 consecutive days, all nonpregnant women were asked to participate including women who were accompanying patients but were nonpatients themselves (31%), 14 (4%) refused to fill in the survey, 35 questionnaires were excluded for incompleteness, 14 were excluded for race other than African-American or white, 14 widowed women and 42 women were excluded for missing marital status data. 74.2% white, 32% had finished college, 7% had less than high school education, 21% single, 67% married and mean age 31.9+/-7.2 years | Pain with menstrual periods currently | Questionnaire (nv) | 96 | | N | 1 | 1 | 482 | 533 | 90  (87.6-92.8) | 75% of those with pain take medication; 8% miss one day of work on average per month | |
| Shah, UK, English, 1994 (94) | | 8 | 21.8-46.2 | Control group of a case control study done between Dec. 1991-August 1992 matched for age, parity and smoking recruited from the genitourinary and family planning clinics (8 HIV seronegative and rest unknown) | Painful menstruation divided into mild-not affecting daily activity and analgesics rarely required, moderate- daily activity occasionally affected but absence from work rare, simple analgesics effective; severe-daily activity clearly affected and poor response to analgesics | Interview (nv) | Not stated | | N | 1 | 1 | 34 | 55 | 61.8  (47.7-74.6) | 14/34 had mild, 14/34 had moderate and 6/35 and severe dysmenorrhea | |
| Walker, USA, English, 1991 (95) | | Not stated | >/=18 | Attenders at Women's clinic (operated by dept. of O&G) and Family medical centre (university based primary care clinic) were approached; patients are racially and ethnically diverse and approximately 20-30% are assisted by public assistance programmes; mean age of patients at Family medical centre more at 37.4+/-16.1yrs vs. 32.1+/-11.2 years, no other significant differences of clinical relevance | Excessive menstrual pain (current or lifetime) | Questionnaire (nv) | 95 | | N | 1 | 1 | 246 | 631 | 39  (35.2-42.9) |  | |
| Stambolov, Bulgaria, Bulgarian, 1988 (96) | | 60 | 19-41 | Women hospitalised in medical institute, Pleven, Bulgaria with a diagnosis of adnexitis between 1979-1984 and 1985-86; controls were healthy fertile women | Painful menstruation (current) | Interview (nv) | 100 | | N | 1 | 1 | 8 | 100 | 8  (3.5-15.1) | 24% in women with history of adnexitis | |
| Cavanagh, USA, English, 1986 (97) | | Not stated | 12 to 18 | Girls with mean age 14.9 years were surveyed during their visit to the Adolescent Clinic at the Upstate Medical Centre; 15 black, 1 Hispanic and 34 white girls; 24 middle and 18 lower income families; 26 lived in the city and 5 in rural areas with 19 from suburbs; 46/50 were postmenarcheal and 24 were menstruating regularly | Dysmenorrhea undefined (current) | Self administered confidential questionnaire (nv) | 92 | | N | 1 | 1 | 22 | 46 | 47.8  (32.9-63.1) | 4/46 sought medical attention | |
| Taner, Turkey, English, 1995 (98) | | 3 | 26-40 | Multiparous women due to undergo sterilization filled a questionnaire, none of them had had any pelvic operation before sterilization, OC and IUDs were discontinued 2 months prior to sterilization | Mild moderate or severe pain during menstruation | Questionnaire (nv) | Not stated | | N | 1 | 0 | 31 | 43 | 72.1  (56.3-84.7) | 35/43 (81.4%) had dysmenorrhea after sterilization; 11, 11 and 9 had mild, moderate and severe dysmenorrhea respectively | |
| Mahmood, UK, English, 1991(99) | | Not stated | Not stated | Questionnaires were sent to patients attending Aberdeen Royal Infirmary who were to have laparoscopy for sterilization, infertility, chronic abdominal or pelvic pain and abdominal hysterectomy for dysfunctional uterine bleeding- data given here is on women having laparoscopy for infertility or sterilization | Dysmenorrhea undefined (current) | Self administered questionnaire (nv) | 98.7 | | N | 1 | 0 | 402 | 910 | 44.2  (40.9-47.5) | 61% with endometriosis, 40% with pelvic adhesions and 41% with normal pelvis had dysmenorrhea | |
| Liu, UK, English, 1986 (100) | | Not stated | 26-48 | Study included 75 patients who were admitted for laparoscopic sterilization during menstruation; they all gave informed consent, the social status was biased towards semi-skilled workers; 7% nulliparous, 14 Para 1, 36 Para 2 | Dysmenorrhea undefined (current) | Questionnaire (nv) | Not stated | | N | 1 | 0 | 41 | 75 | 54.5  (42.7-66.2) |  | |
| Sobczyk, USA, English, 1978 (101) | | Not stated | Not stated | Two age matched study groups were selected from patient records at the Family practice centre of MUSC using the clinic's PDP-15 system from those without a coded diagnosis of dysmenorrhea; half of these were taking Propoxyphene containing medications; 45 and 52% respectively in the two groups were in the 20-29 year age group | Dysmenorrhea in the preceding 2 months | Interview (nv) | 57 | | N | 1 | 0 | 56 | 85 | 65.9  (54.8-75.8) |  | |
| Gray, UK, English, 1960 (102) | | Not stated | 13-17 | Based on a study of 200 private. Patients who presented themselves to the gynecologist for examination; 4 had primary amenorrhea | Dysmenorrhea undefined | Not specified | Not stated | | N | 1 | 0 | 55 | 196 | 28.1  (21.9-34.9) |  | |

**Table 2: Table of included studies on dyspareunia**

| **Author, country, language, year** | **Time period (mo)** | **Age (years)** | **Population characteristics** | **Definition** | **Measurement tool^+^** | **Response**  **rate %** | **Representative**  **(Y-yes, N-no)** | **Country resource*** | **Quality score (out of 5)** | **Cases** | Denominator | **Prevalence (95% CI)** | Notes |
| --- | --- | --- | --- | --- | --- | --- | --- | --- | --- | --- | --- | --- | --- |
| **Community based studies** |  |  |  |  |  |  |  |  |  |  |  |  |  |
| Cain, USA, English, 2003 (103) | Not stated | 42-52 | SWAN is a national study of mid-life women conducted in two phases at 7 US sites. Women age 42-52 either Caucasian or site-designated other ethnicity; having menses within the previous 3 months; having an intact uterus and at least one ovary; no use of medications affecting reproductive hormones within the previous 3 months were recruited in 1996-97. 5 women who responded that they never had sex but reported having intercourse in the last 6 months were excluded from the analyses. Analyses was limited to women who had sex in the last 6 months | Pain or discomfort during intercourse sometimes or always in the last 6 months | Questionnaire (v) | 99 | Y | 1 | 5 | 508 | 2406 | 21.1  (19.5-22.8) | Sample size estimation done |
| Avis N, USA, English, 2000 (104) | 60 | 51-61 | Study sample derived from a substudy of the Massachusetts Women's Health study that began with a large random population based cross sectional survey of 8050 middle-aged women. From this study, a cohort of 543 women aged 45-55 years as of 1.1.82 who had menstruated in the preceding 3 months and not had hysterectomy/ oophorectomy were screened as eligible to participate in this phase | Pain in the pelvic area during or after sexual intercourse during the last 6 months seldom to always | Sexual Activity Questionnaire (v) | 78.6 | Y | 1 | 5 | 46 | 202 | 22.8  (17.2-29.2) |  |
| Golding, USA, English, 1998 (2) | Not stated | Mean 42.3; SE 0.5 | Los Angeles Epidemiologic Catchment Area study- 44.4% latina, 44.2% European American and 11.4% other groups, mean education11.5 years; latino residents were interviewed in English or Spanish according to their preference | Physical pain during sexual relation or not pleasurable sexual relations for many months | Diagnostic interview schedule (v) | 86 | Y | 1 | 4 | 394 | 1428 | 27.6  (25.3-30) |  |
| Golding, USA, English, 1998 (2) | Not stated | Mean 43.9; SE 0.7 | NC-ECA sample was selected to represent adults in 2 mental health catchment areas in North Carolina, one consisting of Durham County, which is primarily urban and the other consisting of 4 contiguous rural counties | Physical pain during sexual relation or not pleasurable sexual relations for many months | Diagnostic interview schedule (v) | 79 | Y | 1 | 4 | 305 | 1703 | 17.9  (16.1-19.8) |  |
| Golding, USA, English, 1998 (2) | Not stated | Mean 46.1; SE 0.8 | In The national study of Health and Life Experiences of women 84.3% were European American with mean education of 12.8 years | Physical pain during sexual relation or not pleasurable sexual relations for many months | Diagnostic interview schedule (v) | 91 | Y | 1 | 4 | 538 | 963 | 56  (55.8-62.1) |  |
| Laumann. USA, English, 1999 (105) | 12 | 18-59 | The national health and social life survey conducted in 1992 is national probability sample of 1410 men and 1749 women between the ages of 18 and 59 years living in households throughout the USA; only those respondents who had a partner in the last 12 months were analysed. Excluded women tended to be older and single. | Painful intercourse over past 12 months | Questionnaire (v) | 79 | Y | 1 | 4 | 230 | 1479 | 15.5  (13.7-17.5) |  |
| Johnson, USA, English, 2004 (106) | 24 | 18-96 | Epidemiological Catchment area project, a multistage probability study of incidence and prevalence of psychiatric disorder; random sample of adult community residents in the St.Louis was queried on DSM III sexual dysfunction, largely Caucasian population, 43% respondents 45 years or older; 22% were currently single an had never married, 7% of the sample reported being in poor health | Physically painful sexual relations ever | Interview (v) | Not stated | Y | 1 | 3 | 19 | 1801 | 3.7  (2.6-5) |  |
| Oberg, Sweden, English, 2004 (107) | Not stated | 18-65 | Nationally representative population of sexually active Swedish women included in a epidemiological investigation; able to communicate in Swedish language, living in Sweden at the time of investigation and mentally and physically capable of participation; 19% did not menstruate of which 95% were over 50 years old | Genital pain in the last 12 months during intercourse | Face to face interview with questionnaire (v) | Not stated | Y | 1 | 3 | 39 | 1056 | 1.1  (0.6-1.6) |  |
| Nazareth, UK, English, 2003 (108) | Not stated | 18-75 | 13/37 approached London GP practices situated in areas of high, medium and low socio-economic deprivation participated. People who consented to take part completed questionnaire, by pen and paper or computer assisted interview; no significant differences in Jarman scores (which indicate socio-economic deprivation) between participating and nonparticipating practices; 71% of those who agreed to participate also agreed for scrutiny of their records; one quarter of the participants were non-white; 11% of the women were bi or homosexual | Pain in genitals during or after sexual intercourse | Modified brief sexual function questionnaire (v) | 71 | Y | 1 | 3 | 31 | 1065 | 3  (2-4.1) |  |
| Kadri, Morocco, English, 2002 (109) | Not stated | 20-80 | Mean age of the sample (n=728) was 36.76+/-12.6 years, 29% had no education, 78% pursued no professional activity and 58% were married; 491/728 women satisfied the criteria "sexually active or having a regular sexual relationship"; 464 responded to this question | Genital pain associated with sexual intercourse (it may also occur before or after intercourse) often or always in the last 6 months | Questionnaire (v) | 94.5 | Y | 2 | 3 | 35 | 464 | 7.5  (5.3-10.3) |  |
| Bhurt, Pakistan, English, 1999 (110) | 6 | 37+/-12.3 | Trained females conducted interviews using a pretested sindhi questionnaire in 8 villages of rural Jamshoro, Sindh. They were ever-married; 74% were illiterate; 83.6% were housewives; 7.5% were nulliparous and 15% had menstrual irregularities | Pain during intercourse during the 6 months prior to interview | Interview (v) | 98 | Y | 2 | 3 | 36 | 738 | 4.9  (3.4-6.7) |  |
| Dunn K, UK, English, 1999 (111) | Not stated | 18-75 | Sample was 4000 people between 18-75 years registered with four general practices diverse in geographical location and level of urbanization; reminders sent to nonresponders, 58% of women were on medications and 24% had anxiety while 6% women suffered from depression | Dyspareunia undefined | Questionnaire with reply paid envelope (v) | 44 | Y | 1 | 3 | 115 | 650 | 17.5  (14.7-20.7) |  |
| Ventegodt, Denmark, English, 1998 (112) | 12 | 18-88 | In 1992, a representative sample was taken from the Danish population from the CPR register by selecting a particular date in the year and then selecting all persons born on that date from 1904 and every fifth year thereafter until 1974 including the year 1961; unclear whether value given is for currently sexually active women or all women | Pain or discomfort during intercourse | Questionnaire (v) | 60.7 | Y | 1 | 3 | 23 | 753 | 3.1  (1.9-4.5) |  |
| Barlow D, UK, English, 1997 (113) | 1 | 55-75 | A six country pan-European study of urogenital ageing, 55.6% married and 3.1% living with partner; 35.9% city dwellers; median age at menopause 50 years for the whole study; 18.5% hysterectomy rate with bilateral oophorectomy in 48.8% of them; 1503/3062 women had been in sexual relationship over the past year. It is unclear whether the results given are for sexually active population or whole age range | Painful sex/sexual relations in the month prior to study | Interview (v) | 65 | Y | 1 | 3 | 64 | 1503 | 4.3  (3.3-5.4) |  |
| Stenberg A Sweden, English, 1996 (114) | 1 | 61 | Study was done in June 1993, all women in the county born during the 14 months Dec 1931 through to Jan 1933 were included; the majority of nonresponders (n=170) did not return the questionnaire. 19% of the non responders were immigrants as compared to 13% of the responders; 632/1076 who responded were sexually active | Dyspareunia undefined | Questionnaire (v) | 84 | Y | 1 | 3 | 259 | 632 | 41  (37.1-44.9) |  |
| Ernst Zurich, Switzerland, English, 1993 (115) | 120 | 20-30 | Sample of the Zurich study was selected in 1978 from a survey of 2201 females reaching age 20 years; randomly selected women were followed up with four interviews in 10 years | Dyspareunia over the previous 1 year | Interview (v) | 72.9 | Y | 1 | 3 | 13 | 216 | 6  (3.2-10.1) |  |
| Lindal, Iceland, English, 1993 (116) | 12 | 55-57 | Between March 1988-89, subjects who were born in 1931 and still living in Iceland on Dec.1, 1986, evenly distributed throughout the country were recruited. Of the 1195 randomly chosen subjects, 90 did not meet criteria for inclusion because they had moved to Iceland in early adulthood or were living abroad and 19 were dead | Functional dyspareunia undefined duration | Diagnostic Interview schedule (v) | 79.3 | Y | 1 | 3 | 13 | 417 | 3.1  (1.7-5.3) |  |
| Osborn, UK, English, 1988 (117) | Not stated | 35-59 | Study was based on two Oxford group practices with computerised age and sex registers which was used to select sample of 600, consisting of 100-150 women in each of five age bands; of the respondents 436 women with male sexual partner were interviewed | Pain or discomfort causing difficulties during sexual intercourse more than half of the time during the previous 3 months | Semistructured interview (v) | 87 | Y | 1 | 3 | 30 | 379 | 8  (5.4-11.1) | 0/86 in 35-39, 1/87 in 40-44, 6/69 in 45-49, 11/65 in 50-54 & 12/72 in 55-59 years age groups |
| Danielsson, Sweden, English, 2003 (118) | 4 | 20-60 | Between Dec.1998-April 1999, all women who participated in the screening programme for cervical cancer in a mixed but chiefly urban region in Sweden were asked to answer a short questionnaire after oral consent was obtained; 7 women who never had intercourse were omitted as were women who did not understand the written language | Dyspareunia that had lasted at least 6 months (ever) | Questionnaire (nv) | 96 | Y | 1 | 2 | 281 | 3017 | 9.3  (8.3-10.4) |  |
| Desai, India, English, 2003 (119) | 6 | 16-50 | This cross sectional study done between Feb-Sept. 2000, included sex workers from a red light area of Surat, India to measure prevalence of STI and HIV; mean age of participants was 28.5 yrs | Dyspareunia causing problems with intercourse | Interview (v) | 95.2 | N | 2 | 2 | 15 | 118 | 12.7  (7.3-20.1) |  |
| Zhao G, China, English, 2000 (120) | Not stated | 41-60 | 402 professional urban women and 404 farmers from rural area out of whom 29.2% were illiterate; mean age at menopause 47.3+/-4.6 years; 1.1% of the women used HRT and they all lived in the city | Painful intercourse undefined | Structured interview (v) | Not stated | Y | 2 | 2 | 158 | 773 | 20.4  (17.6-23.5) |  |
| Thongkrajai, Thailand, English, 1999 (121) | 3 | 15-55 | Healthy women from 4 villages in Nampong district, Khon Kaen Province were recruited via a mobile health clinic to collect data, physical examination and specimen collection to screen for STD, 93.4% married, 85% farmers, 94.5% finished primary school and 20% subjects had abortion | Painful intercourse in the previous 3 months | Interview (nv) | Not stated | Y | 2 | 2 | 124 | 634 | 19.6  (16.5-22.9) |  |
| Jamieson DJ, USA, English, 1996 (93) | 3 | 18-45 | Between Feb-April 1993, two O&G and three family medicine practices were surveyed on 5 consecutive days, all nonpregnant women were asked to participate including women who were accompanying patients but were nonpatients themselves (31%), 14 (4%) refused to fill in the survey, 35 questionnaires were excluded for incompleteness, 14 were excluded for race other than African-American or white, 14 widowed women and 42 women were excluded for missing marital status data. 74.2% white, 32% had finished college, 7% had less than high school education, 21% single, 67% married and mean age 31.9+/-7.2 years | Pain during and / or after intercourse over 1 year | Questionnaire (nv) | 96 | Y | 1 | 2 | 193 | 423 | 45.7  (40.8-50.5) |  |
| Ramoso-Jalbuena, Philippines, English, 1994 (122) | 12 | 40-55 | 90% residing in Metro Manila and10% of the sample was obtained from the private clinic; varied professions, 62% college graduates; 61% in full time employment; age at menarche 13 years and 75% lived with husband; typical respondent of Malay race, 47 years old, 154 cm tall and weighing 54 kg | Painful intercourse undefined | Interview using IHF questionnaire (v) | Not stated | N | 2 | 2 | 58 | 500 | 11.6  (8.9-14.7) | 21/58 (36.2%) consulted doctor18/21 were prescribed medications and 10/18 followed the prescription; 10% subjects recruited from clinic |
| Rekers H, Netherlands, English, 1992 (123) | Not stated | 35-80 | Study was conducted in the city of Zoetermeer with a population mix that reflects composition of Dutch population at large, in conjunction with the Bureau for Population Registration, a stratified sample was drawn from the city register; no consistent differences in the response rate by age, women living in residential homes for the elderly or who were receiving institutional care for other reasons were excluded | Dyspareunia undefined | Mailed questionnaire (nv) | 67.7 | Y | 1 | 2 | 81 | 1299 | 6.2  (5.0-7.7) |  |
| Bang RA, India, English, 1989 (124) | Not stated | Mean 32.1 | Survey conducted in Gadchiroli district of Maharashtra, India in two villages; all females over age 13 years were invited to participate; 25% of the non participating sample surveyed and found to be similar to study sample | Undefined | Interview + examination ( | 59 | Y | 2 | 2 | 43 | 654 | 6.6  (4.8-8.8) |  |
| Berg G, Sweden, English, 1988 (125) | 12 | 60-62 | All women aged 60, 61 and 62 living in the community of Linkoping sent questionnaires, response rate was equally distributed among the three age groups but no further information available on nonresponders; 881/1469 women were sexually active; all women were postmenopausal; 12% nulliparous; 15% had undergone major gynaecological surgery; 7% were receiving estrogens at the time of the study; 60% sexually active, the rate being higher in HRT users. | Local vaginal discomfort resulting in sexual difficulties | Questionnaire (nv) | 72 | Y | 1 | 2 | 287 | 881 | 32.6  (29.5-35.8) |  |
| Garde, Denmark, English, 1980 (126) | 15 | 40 | In 1976-1977, subjects were participants in a health examination intended to illuminate risk factors for illness in a 40 yr old population group (all were born in 1936) selected from the health service registers in particular municipalities in Copenhagen | Pains during intercourse | Interview (nv) | 94 | Y | 1 | 2 | 1 | 79 | 1.3  (0.03-6.8) |  |
| Glatt, USA, English, 1990 (127) | Not stated | Early 30s | Participants for this survey were sought from a group of 500 women who had taken part in a study of sexually transmitted micro-organisms in 1974-75; at the time of study the subjects were students at a large urban university | Discomfort or pain in labial, vaginal or pelvic area during or after intercourse from sexual debut | Questionnaire (nv) | 73.4 | N | 1 | 1 | 105 | 313 | 33.5  (28.3-39.1) | 51 had primary and 54 secondary dyspareunia which developed 10.2 years on average after sexual debut Of 105 women, 22 had dyspareunia rarely, 58 occasionally 17 frequently and 8 always |
| Whorwell, UK, English, 1986 (128) | Not stated | 18-64 | Age, social class matched controls for patients with IBS recruited from staff registers of local commerce and industry after completing a screening questionnaire to exclude subjects with IBS who were about 11% of the group | Dyspareunia undefined | Interview-Questionnaire (nv) | Not stated | N | 1 | 1 | 5 | 90 | 5.55  (1.8-12.5) |  |
| Iosif C, Sweden, English, 1984 (129) | Not stated | 61 | In 1982, out of approximately 3000 women born in 1921, in a defined geographical area in south Sweden, the administrative district of Malmohus county, 1200 were randomly selected from the community register for the present investigation. 21% had undergone gynaecological operations; 4% were currently on HRT and 29.2% had urinary incontinence | Vaginal dryness with difficulty in intercourse | Mailed questionnaire (nv) | 99.2 | N | 1 | 1 | 343 | 902 | 38  (34.8-41.3) |  |
| Abdo CHN, Brazil, English, 2004(130) | 3 | 23.3-37.9 | The Brazilian Study of Sexual Behaviour used a sample of 2835 subjects (53% women and 47% men) in 10 cities of seven Brazilian states. The group studied comprised convenience sample of women with an educational level (high school and college degree) higher than that of the average Brazilian woman; the sample was comparable in terms of race and religion. Subjects were weekend visitors of beaches, parks and shopping malls. Data on women who declined to answer were not recorded and hence comparisons of the answering sample were not taken into account. For statistical analysis, only women who had had sexual intercourse with at least one partner in the past 12 month period were selected | Pain during sexual intercourse (current) | Self administered anonymous questionnaire (nv) | Not stated | N | 2 | 0 | 280 | 1212 | 23.1  (20.8-25.6) |  |
| **Hospital/community clinic based studies** |  |  |  |  |  |  |  |  |  |  |  |  |  |
| Castelo-Branco, Chile, English, 2003 (131) | 5 | 40-64 | Between May-October 2001, consecutive healthy women with mean age 52.4+/-5.7 years attending southern metropolitan health service in Santiago, Chile were included. 82.8% were peri or postmenopausal, 23% had received HRT and 79.2% were sexually active. The inclusion criteria were: healthy women accompanying patients at health centres whose ages were between 40-64 years. Women with mental disorders or who had difficulty in understanding the survey were excluded. An expert committee translated the instrument into Spanish. | Dyspareunia-DSM | Questionnaire (v) | Not stated | N | 2 | 3 | 147 | 423 | 30.2  (25.7-34.6) | Sample size estimation done; 3/27, 35/145, 59/136, 31/68 dyspareunia in 40-44, 45-49, 50-54 &55-59 years age groups respectively |
| Shokrollahi, Iran, English, 1999 (132) | Not stated | 16-53 | Women were selected randomly from all those applying for services at the family planning department of 3 health and treatment canters in Tehran. The participants met 4 criteria: their husbands had only one wife; no recent life crisis; no disorder or illness or drug use that would affect sexual function and they were of reproductive age, not pregnant and had not recently given birth; all participants were Muslim and relatively well educated, 72% had a diploma or university degree | Dyspareunia on most or all occasions | Brief index of sexual functioning for women and sex knowledge and attitude test (v) | Not stated | N | 2 | 2 | 30 | 300 | 10  (6.8-14) | 6% had dyspareunia 50% of the times and 4% always |
| Gurel, Turkey, English, 1999 (92) | Not stated |  | In Jan-June 1995, women with various gynaecological complaints admitted in the dept. were included in the study. All were married and 14 were nulliparous. Those having previous pelvic surgery including caesarean section, those with pelvic mass, uterine anomaly, pregnancy or intrauterine device were excluded | Painful sex/sexual relations in the month prior to study | Questionnaire (nv) | Not stated | N | 2 | 2 | 72 | 235 | 30.6  (24.8-37) |  |
| Rosen, USA, English, 1993 (133) | Not stated | 18-73 | Healthy women mean age 43.6+/-11.9 years, enrolled in a Women's Wellness Centre; two thirds were married or living with partner and most women were employed outside home 28.4% were not sexually active at the time of study, 28% were postmenopausal and 2.4% were pregnant | Dyspareunia undefined | Brief Index of Sexual Satisfaction and Health Background Questionnaire (v) | Not stated | N | 1 | 2 | 65 | 236 | 27.6  (21.9-33.7) |  |
| Robinson J, USA, English, 1992 (134) | 3 | >/=18 | The data are from a three wave panel study of <18 unmarried females, not pregnant, adolescents wishing contraception who attended a Planned Parenthood clinic in Baltimore during 1988, 77% black inner city population, 58% had been pregnant and 12% had given birth | Intercourse is sometimes or always painful | Telephone interview (v) | 89.9 | N | 1 | 2 | 82 | 308 | 26.6  (21.8-31.9) |  |
| Danaci, Turkey, English, 2003 (135) |  |  | Women who attended the GOPD of Celal Bayar university hospital were recruited, exclusion criteria were: having a gynecologic disorder, chronic medical disorder, surgical menopause, psychiatric disorder, using HRT and being illiterate. Women were divided into 3 groups according to their menopausal status: those with regular menses were premenopausal, those with irregular menses in the last year were perimenopausal and those with no menses in the last year were postmenopausal. | Painful intercourse in the last 6 months | Interview on sexual desire, behavior, Beck’s Depression Inventory and STAI (v) | 90 | N | 2 | 1 | 148 | 324 | 45.7  (40.2-51.3) | Postmenopausal- 34/116; Perimenopausal- 24/84; Premenopausal-54/124 |
| Nappi, Italy, English, 2002 (136) | Not stated | 46-60 | Women coming to ten menopause clinics located in different areas of Italy who satisfied the following criteria were consecutively enrolled: being married, having at least 1 child, having a spontaneous menopausal status of at least 6 months with FSH>30 mIU/l, coming for first consultation and being negative for gynaecological diseases Exclusion criteria were: HRT, pelvic surgery, chronic diseases, intake of neuroactive drugs, smoking > 10 cigarettes a day and regular alcohol consumption | Painful intercourse undefined | Visual scale for sexual symptoms questionnaire (v) | Not stated | N | 1 | 1 | 106 | 355 | 29.8  (25.1-34.9) |  |
| El-Defrawi, Egypt, English, 2001(137) | Not stated | Not stated | Patients of maternal and childhood centre, Islamia were gynaecologically examined and psychosexual interview was taken by interviewers who were blind to the woman's circumcision status, 200 circumcised and 50 were uncircumcised | Dyspareunia undefined | Semistructured psychiatric interview based on Arabic version of the sexual behavior Assessment Schedule-adult (SEBAS-A) (v) | Not stated | N | 2 | 1 | 108 | 250 | 43.2  (37-49.6) |  |
| Versi, UK, English, 2001(138) | Not stated | Postmenopausal age group | Women consecutively seen at Dulwich Menopause Clinic in London for non urogenital complaints related to climacteric and had never been on HRT were included; 70 (25%) women reported no sexual activity | Painful intercourse since menopause | Questionnaire (nv) | 93.2 | N | 1 | 1 | 23 | 215 | 10.7  (6.9-15.6) |  |
| Shah, UK, English, 1994 (139) | 8 | 21-46 | Control group of a case control study done between Dec. 1991-August 1992 matched for age, parity and smoking recruited from the genitourinary and family planning clinics (8 HIV seronegative and rest unknown) | Dyspareunia undefined | Interview (nv) | Not stated | N | 1 | 1 | 9 | 51 | 17.6  (8.4-30.8) |  |
| Pepe F, Italy, English, 1991(140) | 6 | 15-58 | In 1987, healthy women who attended a private gynecologist for routine cervical smear and breast examination and who reported a steady heterosexual relationship with only one partner for more than 1 year were approached; no woman had been drug abuser or treated for sexual dysfunction, all were born in Catania or its province, all were roman catholic, most had attended primary school upto different levels, none was postmenopausal, 56.1% were married and 20.5% used Oral contraceptives | Painful coitus | Interview (nv) | 100 | N | 1 | 1 | 40 | 360 | 11.1  (8.1-14.8) |  |
| Mahmood, UK, English, 1991 (141) | Not stated | Not stated | Subjects were patients attending Aberdeen Royal Infirmary who were to have laparoscopy for sterilization, infertility, chronic abdominal or pelvic pain and abdominal hysterectomy for dysfunctional uterine bleeding- data given here is on women having laparoscopy for infertility or sterilization | Dyspareunia (current) of undefined duration | Questionnaire (nv) | 98.7 | N | 1 | 1 | 180 | 910 | 19.8  (17.2-22.5) |  |
| Walker, USA, English, 1991 (95) | Not stated | >18 | In March 1989, attenders at Women's clinic (operated by dept. of O&G) and Family medical centre (university based primary care clinic) were approached; patients were racially and ethnically diverse and approximately 20-30% are assisted by public assistance programmes; mean age of patients at Family medical centre more at 37.4+/-16.1yrs vs. 32.1+/-11.2 years, no other significant differences of clinical relevance | Coital pain (lifetime or current) | Questionnaire (nv) | 95 | N | 1 | 1 | 271 | 631 | 43  (39-46.9) |  |
| Bachmann, USA, English, 1989 (142) | 15 | 18-87 | White, middle class and high school educated women seeking gynecologic evaluation were interviewed over 15 months | Undefined | Interview (nv) | 100 | N | 1 | 1 | 142 | 887 | 16  (13.7-18.6) |  |
| Schein, USA, English, 1988 (143) | Not stated | 18-78 | People attending family practice centre participated in the study on sexual identity and function; mean age 35 years; 70% females; 54% black; 63% married and 37% belonged to social class I and II | Undefined | Questionnaire (v) | Not stated | N | 1 | 1 | 31 | 148 | 21  (14.7-28.4) |  |
| Warner, UK, English, 1987 (144) | 36 | <21->56 | 3 years' work of a co-ordinated clinical service or sexual problems was surveyed; this involved 5 clinical settings and equal distribution between male and female presenters | Undefined | Case records (v) | 100 | N | 1 | 1 | 64 | 577 | 11  (8.6-13.9) |  |
| Heisterberg, Denmark, English, 1986 (145) | 12 | Reproductive age group | The patients included in the present study comprised of women who had participated in a clinical controlled trial in 1978-79 on prophylactic antibiotics and development of postabortal PID. Before the abortion, information about previous births, spontaneous and induced abortions, episodes of PID, and the occurrence of CPP were obtained | Dyspareunia of undefined duration | Questionnaire (nv) | 77 | N | 1 | 1 | 21 | 338 | 6.2  (3.9-9.3) |  |
| Levine, USA, English, 1976 (146) | Not stated | 30-39 | Every third Black woman attending a gynecology clinic at University Hospital of Cleveland for nonsexual complaints were invited to participate, most of whom were born and raised in the South; had completed 10-12 year of formal education; were Protestant; all but 3 were mothers and most were currently not married; most belonged to the lowest socio-economic class | Undefined | Interview (nv) | 78.7 | N | 1 | 1 | 2 | 59 | 3.4  (4.1-11.7) |  |
| Ismael, Malaysia, English, 1994 (147) | Not stated | 40-60 | Study population consisted of 70% Malays, 13% Chinese and 17% Indians; 10% were either patients who came to gynaecology clinic or nursing staff; 76% of these women were married and living with husbands; 23% had ceased sexual activity, 15% were perimenopausal and 34% were postmenopausal | Dyspareunia undefined | Interview/questionnaire (nv) | Not stated | N | 2 | 0 | 32 | 400 | 8  (5.5-11.1) |  |
| Chapman Dudley J, USA, English, 1989 (148) | 20 | Not stated | Between 1986 march to November1987; 30 women were recruited from rape crisis centre and control group from private gynecologic medical practice; 80% white, 13% Hispanic and 7% black. In the geographic area in this study, people in distress can telephone the personal help line or sexual assault line for assistance. After the acute situation was attended to, the women were asked whether they would participate in a sexual and gynecologic evaluation to be repeated every 6 months over 4 year period as were the controls | Pain during arousal or orgasm | Questionnaire (nv) | Not stated | N | 1 | 0 | 3 | 35 | 9  (1.8-2.3) |  |
| Plouffe, Canada, English, 1985 (149) | Not stated | 22-79 | Sexually active women admitted on a elective basis to general gynaecology wards with mean age 37.6+/-1.5 years were studied; 38 were Anglo-American; 39 French; 68 roman catholic; 75 married; 63 not using contraception | Deep dyspareunia of undefined duration | Questionnaire (nv) | Not stated | N | 1 | 0 | 20 | 98 | 20.4  (12.9-29.7) |  |
| Buddeberg, Germany, German, 1984 (150) | 12 | 17-63 | Between 1981-1982, women attending a family practice with a mean age 31.2 years with different problems were approached, majority were employed and 18 were housewives; 67% were lower or middle rank workers | Painful intercourse of undefined duration | Questionnaire (nv) | 45.2 | N | 1 | 0 | 13 | 83 | 15.7  (8.6-25.3) | 3/83 had dyspareunia for < 1 month |

Table 3: Table of studies included in the systematic review on prevalence of noncyclical pelvic pain

| **Author, country, language, year** | **Time**  **period (mo)** | **Age (years)** | **Population characteristics** | **Definition** | **Measurement +** | **Response rate %** | **Representative ness (Y-yes/ N-no)** | **Country resource*** | **Quality score (out of 5)** | **Cases** | **Denominator** | **Prevalence% (95% CI)** | **Notes** |
| --- | --- | --- | --- | --- | --- | --- | --- | --- | --- | --- | --- | --- | --- |
| **Community based studies** | |  |  |  |  |  |  |  |  |  |  |  |  |
| Zondervan, UK, English, 1999 (151) | 60 | 12 to 70 | One hundred and thirty-six general practices in the UK. One practice objected and hence their patients were excluded. Other exclusions were: mental illness (6), participation in the pilot study (5), 810 undelivered questionnaires. Cases were identified randomly on the basis of contact with their general practice pain due to malignancy, chronic inflammatory bowel diseases or pregnancy, or which occurred only during menstruation or sexual intercourse, was excluded | Recurrent or constant pain in the area from navel down in the lower belly of at least 6 months duration unrelated to periods or intercourse. | General practice database(v) | 100 | Y | 1 | 5 |  |  | 2.15 (2.1-2.2) | Monthly prevalence of CPP 21.5/1000 and incidence 1.58/1000 with an annual prevalence of 38.3/1000 Monthly prevalence rates in 15-20 year old 18.2/1000 and >60 27.6/1000. Monthly prevalence in Scotland and Wales were 16/1000 and 29.4/1000 respectively. Sample size estimation done |
| Zondervan, English, 2001(1) | 6 | 18-49 | 4000 women randomly selected from oxfordshire health authority register were sent postal questionnaire | Recurrent or constant pain in the area from navel down in the lower belly of at least 6 months duration unrelated to periods or intercourse | Semistructured questionnaire (v) | 74 | Y | 1 | 4 | 483 | 2016 | 24 (22.1-25.8) | Sample size estimation done; 20% prevalence among 18-25 year old, 28% in 36-40 year olds; lower prevalence in Non Caucasian women (RR 04.) |
| Rulin M, USA, English, 1993 (152) | 18 | Mean 28 | These group of surveyed women formed the control group for sterilised women and were nonsterilised women in natural cycles; mean parity 2.56, 57.7% black, 23.5% Hispanic, 18.2% white | Non cyclical pelvic pain over 3 years excluding dysmenorrhea and dyspareunia | Questionnaire (v) | 78 | Y | 1 | 3 | 13 | 319 | 4.1  (2.2-6.9) | Prospective. 16% one year post sterilization versus 11.6% pre-operatively |
| Bhurt, Pakistan, English, 1999(110) | 3 | 37+/-12.3 | Trained females conducted interviews using a pretested sindhi questionnaire in 8 villages of rural Jamshoro, Sindh, using convenience sampling. They were ever-married; 74% were illiterate, 83.6% were housewives, 7.5% were nulliparous and 15% had menstrual irregularities | Lower abdominal pain with or without vaginal discharge or fever during the 6 months prior to interview | Interview (v) | 98 | N | 2 | 2 | 65 | 738 | 8.8  (8.76-9.28) | Sample size estimation done |
| Thongkrajai, Thailand, English, 1999 (153) | 12 | 15-55 | Healthy women from 4 villages in Nampong district, Khon Kaen Province were randomly recruited via a mobile health clinic to collect data, physical examination and specimen collection to screen for STD, 93.4% married, 85% farmers, 94.5% finished primary school and 20% subjects had abortion | Lower abdominal pain in the previous 3 months | Interview (nv) | Not stated | Y | 2 | 2 | 275 | 634 | 43.4  (39.5-47.3) |  |
| Filippi, Turkey, English, 1997 (154) | 3 | Average 32 | Women randomly sampled from a clinic based community register on current and past contraceptive uptake, reproductive health problems and symptoms of ill-health, pregnant women excluded; all were invited to the health centre for follow-up medical assessment for reproductive morbidities. Those who refuse the medical phase were younger, better educated and less likely to report ill-health | Abnormal vaginal discharge and lower abdominal pain in the previous three months | Questionnaire (v) | 80 | N | 2 | 2 | 139 | 694 | 20  (17.1-23.2) |  |
| Jamieson DJ, USA, English, 1996 (93) | 3 | 18 to 47 | In February-April 1993, two O&G and three family medicine practices were surveyed on 5 consecutive days, all nonpregnant women were asked to participate including women who were accompanying patients but were nonpatients themselves (31%), 14 (4%) refused to fill in the survey, 35 questionnaires were excluded for incompleteness, 14 were excluded for race other than African-American or white, 14 widowed women and 42 women were excluded for missing marital status data. 74.2% white, 32% had finished college, 7% had less than high school education, 21% single, 67% married and mean age 31.9+/-7.2 years | Pain in lower abdomen not counting menstrual pain over 1 year | Questionnaire (nv) | 96 | Y | 1 | 2 | 227 | 581 | 39  (35.1-43.2) | 16% of overall sample (45.3% of the sufferers) took medication for nonmenstrual, noncoital pain |
| Mathias, USA, English, 1996 (155) | 2 | 18 to 50 | In April-May 1994, the Gallup organisation telephoned 17927 households by random digit dialling to identify women between 18-50 years; in women with more than one eligible woman, a computer generated algorithm identified the woman to be interviewed; demographic profile of the survey participants with cpp was similar to women with no cpp in terms of age, race, ethnicity, education (i.e. mean age 35.7+/-8.6 versus 36.7+/-8.6, 37% of women in both groups had less than a high school education, 10 vs. 11% had an advanced college degree, 86 vs. 82% were white, 7versus 9% were African American, 4 vs. 5% were latino. Responses from pregnant and postmenopausal women were excluded | Chronic pelvic pain for at least 6 months experienced off and on or constantly in the last 3 months | Telephone interview (nv) | 89 | Y | 1 | 2 | 773 | 5325 | 14.7  (13.6-15.8) | 25% has seen some health care provider in the last 3 months; total indirect cost of cpp due to time lost from work $555.3 million and estimated direct medical costs for US population of 18-50 yr old women are $881.5 million per year. |
| Bhatia, India, English 1995 (156) | Not stated | 16 to 35 | Study conducted in 1991 in the subdistrict of Karnataka state, 70 km from Bangalore on women who had at least one child younger than five.2400 from rural areas; all eligible women living in the town and in the 48 villages having population of at least 500 persons were included | Lower abdominal pain or vaginal discharge with fever between 3 to 13+ months | Interview (nv) | 95 | Y | 2 | 2 | 187 | 3600 | 5.2  (7.1-8.9) |  |
| Kirkengen, Norway, English, 1993 (157) | Not stated | 20 to 49 | Women recruited from a single general practice in Oslo in 1989-1990, they were consecutive attenders for gynaecological problem other than pregnant or after abortion or delivery; 9 women with poor knowledge of Norwegian were excluded | Pelvic pain undefined for 12 months | Interview (v) | 57 | N | 2 | 2 | 22 | 85 | 25.9  (17-36.5) |  |
| Bang RA, India, English, 1989 (158) | Not stated | Over 13, mean 32.1 | Survey conducted in Gadchiroli district of Maharashtra, India in two villages; all females over age 13 years were invited to participate; 25% of the non participating sample surveyed and found to be similar to study sample | Lower abdominal pain of undefined duration | Interview + examination (v) | 59 | Y | 2 | 2 | 86 | 650 | 13.2  (10.7-16) |  |
| Frljak, Bosnia, English, 1997 (159) | 12 | 20 to 40 | Information from of gynaecological consultations in 4 different periods during 1993/94 on women attending women's Therapy Centre, Zenica, Bosnia in the war period | Pelvic pain undefined | Hospital records (v) | Not stated | N | 1 | 2 | 55 | 486 | 11.3  (8.6-14.5) |  |
| Desai, India, English, 2003 (160) | 7 | 16 to 50 | This cross sectional study of volunteers done between Feb.-Sept. 2003 included sex workers from a red light area of Surat, India to measure prevalence of STI and -HIV; mean age of participants was 28.5 yrs | Pain in lower abdomen of undefined duration | Interview (v) | 95 | N | 2 | 1 | 23 | 118 | 19.5  (12.8-27.8) | Sex workers community |
| Gurel, Turkey, English, 1999 (92) | 6 | 18 to 56 | In Jan-June 1995, women with various gynaecological complaints admitted in the dept. were included in the study. All were married and 14 were nulliparous. Those having previous pelvic surgery including caesarean section, those with pelvic mass, uterine anomaly, pregnancy or intrauterine device were excluded | Noncyclic pelvic pain for 6 months or longer influencing daily life or requiring analgesia | Questionnaire (nv) | Not stated | N | 2 | 1 | 189 | 235 | 80  (74.8-85.3) |  |
| Mahmood, UK, English, 1991 (161) | Not stated | Reproductive age | Subjects were patients consecutively attending Aberdeen Royal Infirmary who were to have laparoscopy for sterilisation, infertility, chronic abdominal or pelvic pain and abdominal hysterectomy for dysfunctional uterine bleeding- data given here is on women having laparoscopy for infertility or sterilisation | Noncyclical pelvic pain (current) of undefined duration | Questionnaire (nv) | 99 | N | 1 | 1 | 391 | 910 | 43  (39.6-46.1) |  |
| Walker, USA, English, 1991 (95) | 1 | 18 or over | In March 1989, attenders at Women's clinic (operated by dept. of O&G) and Family medical centre (university based primary care clinic) were consecutively approached; patients were racially and ethnically diverse and approximately 20-30% are assisted by public assistance programmes; mean age of patients at Family medical centre more at 37.4+/-16.1yrs vs. 32.1+/-11.2 years, no other significant differences of clinical relevance | Chronic or intermittent pelvic pain undefined-lower abdominal pain, that is, pain in any part of belly from navel down to private parts (lifetime or current) | Questionnaire (nv) | 95 | N | 1 | 1 | 247 | 651 | 38  (35.8-43.6) |  |
| Heisterberg, Denmark, English, 1986 (162) | Not stated | Reproductive age | The patients included in the present study comprised of consecutive women who had participated in a clinical controlled trial in 1978-79 on prophylactic antibiotics and development of postabortal PID. Before the abortion, information about previous births, spontaneous and induced abortions, episodes of PID, and the occurrence of CPP were obtained | Chronic pelvic pain (CPP) excluding dysmenorrhea and dyspareunia of undefined duration | Questionnaire (nv) | 77 | N | 1 | 1 | 22 | 352 | 6.2  (3.9-9.3) | CPP rate for those with postabortal PID was 4/29 (13.8%) |
| Iglesias, Mexico, English, 1980 (163) | Not stated | Not stated | Medical service of the company polled at random airline stewardesses at the time they applied for assistance of a variety of clinical problems, personnel using contraception or suffering from obvious general or gynaecological problems were excluded | Current pelvic pain or congestion after long flights | Questionnaire (nv) | 100 | N | 2 | 1 | 76 | 200 | 38  (31.2-41.1) |  |

*Country resource-1: developed; 2-less developed; 3-least developed

^+^ v-validated; nv- not validated

Table 4: Metaregression to explore heterogeneity in the systematic review on prevalence of chronic pelvic pain

| **Reasons for heterogeneity** | **Dysmenorrhea**  **(n=101)**  **Coefficient (P value)** | **Dyspareunia**  **(n=54)**  **Coefficient (P value)** | **Noncyclical pain**  **(n=17)**  **Coefficient (P value)** |
| --- | --- | --- | --- |
| **Univariate analysis** |  |  |  |
|  |  |  |  |
| Development status of country | -0.184 (0.187) | 0.203 (0.462) | 0.127 (0.767) |
| Age group* | -0.133 (0.154) | -0.210 (0.285) | - |
| Representative sample | -0.181 (0.893) | *-0.559 (0.016)* | -0.144 (0.362) |
|  |  |  |  |
| Quality items |  |  |  |
| Prospective design | -0.116 (0.594) | 0.125 (0.766) | -1.571 (0.07) |
| Validated measurement tool | *0.305 (0.013)* | -0.245 (0.312) | -0.526 (0.209) |
| Adequate sampling method | -0.414 (0.758) | -0.173 (0.558) | -0.101 (0.858) |
| Sample size estimation | 0.255 (0.480) | 0.177 (0.584) | -0.573 (0.292) |
| Response rate > 80% | 0.105 (0.424) | 0.435 (0.076) | 0.471 (0.309) |
|  |  |  |  |
| Quality score out of 5 | 0.090 (0.159) | 0.041 (0.720) | -0.213 (0.329) |
|  |  |  |  |
| Quality score > 2/5 | 0.282 (0.099) | 0.135 (0.632) | -0.345 (0.194) |
|  |  |  |  |
| **Multivariate analysis** |  |  |  |
|  |  |  |  |
| Development status of country | -0.213 (0.160) | 0.235 (0.422) | 0.101 (0.816) |
| Age group* | -0.075 (0.499) | -0.214 (0.289) | - |
| Representative sample | -0.115 (0.429) | *-0.756 (0.007)* | -0.394 (0.376) |
|  |  |  |  |
| Quality items |  |  |  |
| Prospective design | -0.117 (0.622) | 0.212 (0.611) | -0.696 (0.549) |
| Validated measurement tool | 0.267 (0.062) | -0.245 (0.312) | -0.589 (0.355) |
| Adequate sampling method | -0.058 (0.712) | 0.140 (0.681) | -0.140 (0.868) |
| Sample size estimation | 0.169 (0.662) | 0.548 (0.113) | -0.453 (0.623) |
| Response rate > 80% | 0.123 (0.422) | 0.343 (0.166) | 0.069 (0.886) |
|  |  |  |  |
| Quality score out of 5 | 0.108 (0.164) | 0.204 (0.09) | -0.469 (0.050) |
|  |  |  |  |
| Quality score > 2/5+ | 0.244 (0.200) | 0.503 (0.081) | *-0.901 (0.023)* |

Age group cutoff for dysmenorrhea < 25 and for dyspareunia >60 years

# Reference List of included studies

Reference List

(1) Zondervan KT, Yudkin PL, Vessey MP, Jenkinson CP, Dawes MG, Barlow DH et al. The community prevalence of chronic pelvic pain in women and associated illness behaviour. Br J Gen Pract 2001; 51(468):541-547.

(2) Golding JM, Wilsnack SC, Learman LA. Prevalence of sexual assault history among women with common gynecologic symptoms. Am J Obstet Gynecol 1998; 179(4):1013-1019.

(3) Flug D, Largo RH, Prader A. Symptoms related to menstruation in adolescent Swiss girls: a longitudinal study. Ann Hum Biol 1985; 12(2):161-168.

(4) Wilson CA, Keye WR, Jr. A survey of adolescent dysmenorrhea and premenstrual symptom frequency. A model program for prevention, detection, and treatment. Journal of Adolescent Health Care 1989; 10(4):317-322.

(5) Andersch B, Milsom I. An epidemiologic study of young women with dysmenorrhea. Am J Obstet Gynecol 1982; 144(6):655-660.

(6) Barnard K, Frayne SM, Skinner KM, Sullivan LM. Health status among women with menstrual symptoms. J Womens Health (Larchmt ) 2003; 12(9):911-919.

(7) Gordley LB, Lemasters G, Simpson SR, Yiin JH. Menstrual disorders and occupational, stress, and racial factors among military personnel. Journal of Occupational & Environmental Medicine 2000; 42(9):871-881.

(8) Holmlund U. The experience of dysmenorrhea and its relationship to personality variables. Acta Psychiatr Scand 1990; 82(2):182-187.

(9) Mishra GD, Dobson AJ, Schofield MJ. Cigarette smoking, menstrual symptoms and miscarriage among young women. Australian & New Zealand Journal of Public Health 2000; 24(4):413-420.

(10) Sultan C, Parisi F, Feki M, Rasandratana A, Attal G, Legasal P et al. Epidemiology of dysmenorrhea in adolescents in France. Annales de Pediatrie 1999; 46(8):518-525.

(11) Pedron-Nuevo N, Gonzalez-Unzaga LNM, Celis-Carrillo R, Reynoso-Isla M, Torre-Romeral L. Frequency of dysmenorrhea and associated symptoms in women of 12 to 24 years. Ginecologia y Obstetricia de Mexico, Vol 66(DEC 1998; )(pp 492-494).

(12) Vicdan K, Kukner S, Dabakoglu T, Ergin T, Keles G, Gokmen O. Demographic and epidemiologic features of female adolescents in Turkey. Journal of Adolescent Health 1996; 18(1):54-58.

(13) Ng TP, Tan NC, Wansaicheong GK. A prevalence study of dysmenorrhoea in female residents aged 15-54 years in Clementi Town, Singapore. Annals of the Academy of Medicine, Singapore 1992; 21(3):323-327.

(14) Ng TP, Tan NC, Wansaicheong GK. A prevalence study of dysmenorrhoea in female residents aged 15-54 years in Clementi Town, Singapore. Annals of the Academy of Medicine, Singapore 1992; 21(3):323-327.

(15) Cronje HS, Kritzinger IE. Menstruation: symptoms, management and attitudes in university students. Int J Gynaecol Obstet 1991; 35(2):147-150.

(16) Robinson JC, Plichta S, Weisman CS, Nathanson CA, Ensminger M. Dysmenorrhea and use of oral contraceptives in adolescent women attending a family planning clinic. American Journal of Obstetrics & Gynecology 1992; 166(2):578-583.

(17) Sundell G, Milsom I, Andersch B. Factors influencing the prevalence and severity of dysmenorrhoea in young women. BJOG 1990; 97(7):588-594.

(18) Bang RA, Bang AT, Baitule M, Choudhary Y, Sarmukaddam S, Tale O. High prevalence of gynaecological diseases in rural Indian women. Lancet 1989; 1(8629):85-88.

(19) Fisher M, Trieller K, Napolitano B. Premenstrual symptoms in adolescents. J Adolesc Health Care 1989; 10(5):369-375.

(20) Teperi J, Rimpela M. Menstrual pain, health and behaviour in girls. Social Science & Medicine 1989; 29(2):163-169.

(21) Scambler A, Scambler G. Menstrual symptoms, attitudes and consulting behaviour. Soc Sci Med 1985; 20(10):1065-1068.

(22) Woods NF, Most A, Dery GK. Prevalence of perimenstrual symptoms. Am J Public Health 1982; 72(11):1257-1264.

(23) Klein JR, Litt IF. Epidemiology of adolescent dysmenorrhea. Pediatrics 1981; 68(5):661-664.

(24) Sehgal K, Marwah S, Tiwari I. Symptoms associated with menstruation and some correlates of dysmenorrhoea in college girls. Journal of obstetrics and gynaecology of India 1972; 22:323-329.

(25) Weissman AM, Hartz AJ, Hansen MD, Johnson SR. The natural history of primary dysmenorrhoea: a longitudinal study. BJOG 2004; 111(4):345-352.

(26) Hirata M, Kumabe K, Inoue Y. Relationship between the frequency of menstrual pain and bodyweight in female adolescents. [Japanese]. Nippon Koshu Eisei Zasshi - Japanese Journal of Public Health 2002; 49(6):516-524.

(27) Walraven G, Ekpo G, Coleman R, Scherf C, Morison L, Harlow SD. Menstrual disorders in rural Gambia. Stud Fam Plan 2002; 33(3):261-268.

(28) Larroy C, Crespo M, Meseguer C. Functional dysmenorrhea in the Autonomous Community of Madrid: Study of prevalence according to age. Revista de la Sociedad Espanola del Dolor 2001; 8(1):11-22.

(29) Wu D, Wang X, Chen D, Niu T, Ni J, Liu X et al. Metabolic gene polymorphisms and risk of dysmenorrhea. Epidemiology 2000; 11(6):648-653.

(30) González Bahamonde M, Ibarra Farías M. Conocimientos y prácticas de autocuidado sobre síndrome premenstrual y dismenorrea de un grupo de alumnas de la Facultad de Educación de la Pontificia Universidad Católica de Chile / Selfcare knowledge and practice about premenstrual syndrome and dysmenorrea in a group of female students from Facultad de Educación, Pontificia Universidad Católica de Chile. 1999.

(31) Kritz SD, Wingard DL, Garland FC. The association of behavior and lifestyle factors with menstrual symptoms. Journal of Women's Health & Gender Based Medicine 1999; 8(9):1185-1193.

(32) Campbell MA, McGrath PJ. Use of medication by adolescents for the management of menstrual discomfort. Arch Pediatr Adolesc Med 1997; 151(9):905-913.

(33) Elahi N, Parveen N. Menstrual disorders in adolescent age group. Journal of the College of Physicians and Surgeons Pakistan 1997; 7(3):105-107.

(34) Harlow SD, Park M. A longitudinal study of risk factors for the occurrence, duration and severity of menstrual cramps in a cohort of college women. British Journal of Obstetrics & Gynaecology 1996; 103(11):1134-1142.

(35) Mathias SD, Kuppermann M, Liberman RF, Lipschutz RC, Steege JF. Chronic pelvic pain: prevalence, health-related quality of life, and economic correlates. Obstet Gynecol 1996; 87(3):321-327.

(36) Skierska E, Leszczynska-Bystrzanowska J, Gajewski AK. [Risk analysis of menstrual disorders in young women from urban population]. [Polish]. Przeglad Epidemiologiczny 1996; 50(4):467-474.

(37) Onatra HW, Posso VHJ. Dysmenorrhea behavior in three groups of teenagers belonging to a different social status. Revista Colombiana de Obstetricia y Ginecologia 1994; 45(3):249-254.

(38) Lee KA, Rittenhouse CA. Prevalence of perimenstrual symptoms in employed women. Women & Health 1991; 17(3):17-32.

(39) Odujinrin OM, Ekunwe EO. Epidemiologic survey of menstrual patterns amongst adolescents in Nigeria. West African Journal of Medicine 1991; 10(3-4):244-249.

(40) Mijanovic D. [Correlation between certain factors in maturation and primary dysmenorrhea in adolescence]. [Serbo-Croatian (Roman)]. Jugoslavenska Ginekologija i Perinatologija 1990; 30(3-4):79-82.

(41) Thomas KD, Okonofua FE, Chiboka O. A study of the menstrual patterns of adolescents in Ile-Ife, Nigeria. International Journal of Gynaecology & Obstetrics 1990; 33(1):31-34.

(42) Busch CM, Costa Jr PT, Whitehead WE, Heller BR. Severe perimenstrual symptoms: Prevalence and effects on absenteeism and health care seeking in a non-clinical sample. Women & Health 1988; 14(1):59-74.

(43) Johnson J. Level of knowledge among adolescent girls regarding effective treatment for dysmenorrhea. J Adolesc Health 1988; 9(5):398-402.

(44) Pullon S, Reinken J, Sparrow M. Prevalance of dysmenorrhoea in Wellington women. New Zealand Medical Journal 1988; 101(839):52-54.

(45) Silberg JL, Martin NG, Heath AC. Genetic and environmental factors in primary dysmenorrhea and its relationship to anxiety, depression, and neuroticism. Behav Genet 1987; 17(4):363-383.

(46) Whorwell PJ, McCallum M, Creed FH, Roberts CT. Non-colonic features of irritable bowel syndrome. Gut 1986; 27(1):37-40.

(47) Mergler D, Vezina N. Dysmenorrhea and cold exposure. Journal of Reproductive Medicine 1985; 30(2):106-111.

(48) Svanberg L, Ulmsten U. The incidence of primary dysmenorrhea in teenagers. Archives of Gynecology 1981; 230(3):173-177.

(49) Webster SK, Martin HJ, Uchalik D, Gannon L. The Menstrual Symptom Questionnaire and spasmodic/congestive dysmenorrhea: measurement of an invalid construct. J Behav Med 1979; 2(1):1-19.

(50) Wood C, Larsen L, Williams R. Menstrual characteristics of 2,343 women attending the Shepherd foundation. Australian and New Zealand Journal of Obstetrics and Gynaecology, Vol 19(2) (pp 107-110), 1979 1979.

(51) Theano G. The prevalence of menstrual symptoms in Spanish students. Br J Psychiatry 1968; 114(511):771-773.

(52) Hirt M, Kurtz R, Ross WD. The relationship between dysmenorrhea and selected personality variables. Psychosomatics 1967; 8(6):350-353.

(53) Widholm O, Frisk M, Tenhunen T, Hortling H. Gynecological findings in adolescence. A study of 514 patients. Acta Obstet Gynecol Scand 1967; 46:Suppl-27.

(54) Kessel N, Coppen A. The prevalence of common menstrual symptoms. Lancet 1963; 2:61-64.

(55) Golub LJ., Lang WR, Menduke H. The incidence of dysmenorrhea in high school girls. Postgrad Med 1958; 23(1):38-40.

(56) Pawlowski B. Prevalence of menstrual pain in relation to the reproductive life history of women from the Mayan rural community. Ann Hum Biol 2004; 31(1):1-8.

(57) Schmidt E, Herter LD. Dismenorréia em adolescentes escolares / Dysmenorrhea in school adolescents . Adolesc latinoam 2002; 3(1):ISSN 1414-7130.

(58) Dusek T. Influence of high intensity training on menstrual cycle disorders in athletes. Croatian Medical Journal 2001; 42(1):79-82.

(59) Balbi C, Musone R, Menditto A, Di Prisco L, Cassese E, D'Ajello M et al. Influence of menstrual factors and dietary habits on menstrual pain in adolescence age. Eur Jf Obstet Gynecol Reprod Biol 2000; 91(2):143-148.

(60) Banikarim C, Chacko MR, Kelder SH. Prevalence and impact of dysmenorrhea on hispanic female adolescents. Arch Pediatr Adolesc Med 2000; 154(12):1226-1229.

(61) Hillen TI, Grbavac SL, Johnston PJ, Straton JA, Keogh JM. Primary dysmenorrhea in young Western Australian women: prevalence, impact, and knowledge of treatment. Journal of Adolescent Health 1999; 25(1):40-45.

(62) Okonofua FE, Balogun JA, Ayangade SO, Fawole JO. Exercise and menstrual function in Nigerian university women. African Journal of Medicine & Medical Sciences 1990; 19(3):185-190.

(63) Visvanathan N, Wyshak G. Tubal ligation, menstrual changes, and menopausal symptoms. J Womens Health Gend Based Med 2000; 9(5):521-527.

(64) Thongkrajai P, Pengsaa P, Lulitanond V. An epidemiological survey of female reproductive health status: gynecological complaints and sexually-transmitted diseases. Southeast Asian J Trop Med Public Health 1999; 30(2):287-295.

(65) Jarrett M, Heitkemper MM, Shaver JF. Symptoms and self-care strategies in women with and without dysmenorrhea. Health Care Women Int 1995; 16(2):167-178.

(66) Messing K, Saurel-Cubizolles MJ, Bourgine M, Kaminski M. Factors associated with dysmenorrhea among workers in French poultry slaughterhouses and canneries. Journal of Occupational Medicine 1993; 35(5):493-500.

(67) Izzo A, Labriola D. Dysmenorrhoea and sports activities in adolescents. Clinical & Experimental Obstetrics & Gynecology 1991; 18(2):109-116.

(68) Bukman A, Hemelhorst FM, Hengeveld MW. Prevalence and experience of dysmenorrhea in infertility patients. J psychosom obstet gynaecol 1990; 11(2):147-153.

(69) Clarvit SR. Stress and menstrual dysfunction in medical students. Psychosomatics 1988; 29(4):404-409.

(70) Gruber VA, Wildman BG. The impact of dysmenorrhea on daily activities. Behav Res Ther 1987; 25(2):123-128.

(71) Iglesias R, Terres A, Chavarria A. Disorders of the menstrual cycle in airline stewardesses. Aviat Space Environ Med 1980; 51(5):518-520.

(72) Widholm O. Dysmenorrhea during adolescence. Acta Obstet Gynecol Scand Suppl 1979; 87:61-66.

(73) Malina RM, Spirduso WW, Tate C, Baylor AM. Age at menarche and selected menstrual characteristics in athletes at different competitive levels and in different sports. Medicine & Science in Sports 1978; 10(3):218-222.

(74) Sheldrake P, Cormack M. Variations in menstrual cycle symptom reporting. J Psychosom Res 1976; 20(3):169-177.

(75) Bergsjo P, Jenssen H, Vellar OD. Dysmenorrhea in industrial workers. Acta Obstet Gynecol Scand 1975; 54(3):255-259.

(76) Widholm O, Kantero R. A statistical analysis of the menstrual patterns of 8000 Finnish girls and their mothers. Acta Obstet Gynecol Scand 1971; 14(Suppl 14):1-36.

(77) Clow A. Discussion on dysmenorrhea in young women: its incidence, prevention and treatment. BMJ 1924; 2:558-561.

(78) Rojas J, Robles C, Rojas N. Dismenorrea en la adolescencia. Rev colomb obstet ginecol 1997; 48(2):95-105.

(79) Shye D, Jaffe B. Prevalence and correlates of perimenstrual symptoms: a study of Israeli teenage girls. J Adolesc Health 1991; 12(3):217-224.

(80) Yang JM, Chen QY, Jiang XZ. Effects of metallic mercury on the perimenstrual symptoms and menstrual outcomes of exposed workers. Am J Ind Med 2002; 42(5):403-409.

(81) Nuñez Troconis JT. Trastornos menstruales en estudiantes universitarias: II Menarquia y dismenorrea / Menstrual upsetting in the female universitary student: II Menarch and Dysmenorrhea . Rev obstet ginecol Venezuela 1991; 51(2):105-108.

(82) Peña Nina DE, Mora Cabrera RA, Chalas A. La dismenorrea como causa de ausentismo y bajo rendimiento laboral / Dismenorrea : cause of absence and low production at work . Acta méd domin 1993; 15(3):92-96.

(83) Montero P, Bernis C, Fernandez V, Castro S. Influence of body mass index and slimming habits on menstrual pain and cycle irregularity. Journal of Biosocial Science 1996; 28(3):315-323.

(84) Barros ACSDd, Takemoto AK, Coronado MRG, Marques JA, Nishimura A. Caracterizaçäo epidemiológica da dismenorréia / Dysmenorrhea epidemiological characterization . Rev IATROS 1987; 6(1):13-19.

(85) Lemasters GK, Hagen A, Samuels SJ. Reproductive outcomes in women exposed to solvents in 36 reinforced plastics companies. I. Menstrual dysfunction. J Occup Med 1985; 27(7):490-494.

(86) Toriola AL, Mathur DN. Menstrual dysfunction in Nigerian athletes. British Journal of Obstetrics & Gynaecology 1986; 93(9):979-985.

(87) Sogbanmu MO, Aregbesola YA. Menarchal age in Nigerian schoolgirls: its relationship to their height, weight and menstrual profile. Int J Gynaecol Obstet 1978; 16(4):339-340.

(88) Khatri R, Gupta AN. Effect of childbirth on menstrual pattern. Indian J Med Res 1978; 67:66-72.

(89) Timonen S, Procope BJ. The premenstrual syndrome; frequency and association of symptoms. Ann Chir Gynaecol Fenn 1973; 62(3):108-116.

(90) Rulin MC, Davidson AR, Philliber SG, Graves WL, Cushman LF. Long-term effect of tubal sterilization on menstrual indices and pelvic pain. Obstet Gynecol 1993; 82(1):118-121.

(91) El Defrawi, Dandash KF, Refaat AH, Eyada M. Female genital mutilation and its psychosexual impact. Journal of Sex & Marital Therapy 2001; 27(5):465-473.

(92) Gurel H, Gurel SA. Dyspareunia, back pain and chronic pelvic pain: The importance of this pain complex in gynecological practice and its relation with grandmultiparity and pelvic relaxation. Gynecologic & Obstetric Investigation 1999; 48(2):119-122.

(93) Jamieson DJ, Steege JF. The prevalence of dysmenorrhea, dyspareunia, pelvic pain, and irritable bowel syndrome in primary care practices. Obstet Gynecol 1996; 87(1):55-58.

(94) Shah PN, Smith JR, Wells C, Barton SE, Kitchen VS, Steer PJ. Menstrual symptoms in women infected by the human immunodeficiency virus. Obstet Gynecol 1994; 83(3):397-400.

(95) Walker EA, Katon WJ, Jemelka R, Alfrey H, Bowers M, Stenchever MA. The prevalence of chronic pelvic pain and irritable bowel syndrome in two university clinics. J Psychosom Obstet Gynecol 1991; 12(SUPPL.):65-75.

(96) Stambolov B. [Adnexal inflammatory diseases and their influence on menstrual function]. [Bulgarian]. Akusherstvo i Ginekologiia 1989; 28(4):35-37.

(97) Cavanaugh RM, Jr. Obtaining a personal and confidential history from adolescents. An opportunity for prevention. J Adolesc Health Care 1986; 7(2):118-122.

(98) Taner CE, Hakverdi AU, Erden AC, Satici O. Menstrual disorders and pelvic pain after sterilization. Adv Contracept 1995; 11(4):309-315.

(99) Mahmood TA, Templeton AA, Thomson L, Fraser C. Menstrual symptoms in women with pelvic endometriosis. BJOG 1991; 98(6):558-563.

(100) Liu DT, Hitchcock A. Endometriosis: its association with retrograde menstruation, dysmenorrhoea and tubal pathology. BJOG 1986; 93(8):859-862.

(101) Sobczyk R, Braunstein ML, Solberg L, Schuman SH. A case control survey and dysmenorrhea in a family practice population: a proposed disability index. J Fam Pract 1978; 7(2):285-290.

(102) Gray LA. Gynecology in adolescence. Pediatric clinics of North America 1960; 7:43-63.

(103) Cain VS, Johannes CB, Avis NE, Mohr B, Schocken M, Skurnick J et al. Sexual functioning and practices in a multi-ethnic study of midlife women: Baseline results from SWAN. Journal of Sex Research 2003; 40(3):266-276.

(104) Avis NE, Stellato R, Crawford S, Johannes C, Longcope C. Is there an association between menopause status and sexual functioning? Menopause 2000; 7(5):297-309.

(105) Laumann EO, Paik A, Rosen RC. Sexual dysfunction in the United States: prevalence and predictors. JAMA 1999; 281(6):537-544.

(106) Johnson SD, Phelps DL, Cottler LB. The association of sexual dysfunction and substance use among a community epidemiological sample. Arch Sex Behav 2004; 33(1):55-63.

(107) Oberg K, Fugl-Meyer AR, Fugl-Meyer KS. On categorization and quantification of women's sexual dysfunctions: an epidemiological approach. Int J Impot Res 2004; 16(3):261-269.

(108) Nazareth I, Boynton P, King M. Problems with sexual function in people attending London general practitioners: cross sectional study. BMJ 2003; 327(7412):423.

(109) Kadri N, McHichi Alami KH, McHakra TS. Sexual dysfunction in women: population based epidemiological study. Archives of Women's Mental Health 2002; 5(2):59-63.

(110) Bhurt, AW, Fikree A, Channa GZ, Soomro R, Bhurt n. Prevalence and Risk Factors of Symptoms of Pelvic Inflammatory Disease in a Rural Community of Jamshoro, Sindh, Pakistan. J Pak Med Assoc 1999; 49(8):188-194.

(111) Dunn KM, Croft PR, Hackett GI. Association of sexual problems with social, psychological, and physical problems in men and women: a cross sectional population survey. Journal of Epidemiology & Community Health 1999; 53(3):144-148.

(112) Ventegodt S. Sex and the quality of life in Denmark. Arch Sex Behav 1998; 27(3):295-307.

(113) Barlow DH, Samsioe G, van Geelen JM. A study of European womens' experience of the problems of urogenital ageing and its management. Maturitas 1997; 27(3):239-247.

(114) Stenberg A, Heimer G, Ulmsten U, Cnattingius S. Prevalence of genitourinary and other climacteric symptoms in 61-year-old women. Maturitas 1996; 24(1-2):31-36.

(115) Ernst C, Foldenyi M, Angst J. The Zurich Study: XXI. Sexual dysfunctions and disturbances in young adults. Data of a longitudinal epidemiological study. European Archives of Psychiatry & Clinical Neuroscience 1993; 243(3-4):179-188.

(116) Lindal E, Stefansson JG. The lifetime prevalence of psychosexual dysfunction among 55 to 57-year-olds in Iceland. Social Psychiatry & Psychiatric Epidemiology 1993; 28(2):91-95.

(117) Osborn M, Hawton K, Gath D. Sexual dysfunction among middle aged women in the community. BMJ 1988; British Medical Journal. 296(6627):959-962.

(118) Danielsson I, Sjoberg I, Stenlund H, Wikman M. Prevalence and incidence of prolonged and severe dyspareunia in women: results from a population study. Scandinavian Journal of Public Health 2003; 31(2):113-118.

(119) Desai VK, Kosambiya JK, Thakor HG, Umrigar DD, Khandwala BR, Bhuyan KK. Prevalence of sexually transmitted infections and performance of STI syndromes against aetiological diagnosis, in female sex workers of red light area in Surat, India. Sexually Transmitted Infections 2003; 79(2):111-115.

(120) Zhao G, Wang L, Yan R, Dennerstein L. Menopausal symptoms: experience of Chinese women. Climacteric 2000; 3(2):135-144.

(121) Thongkrajai P, Pengsaa P, Lulitanond V. An epidemiological survey of female reproductive health status: gynecological complaints and sexually-transmitted diseases. Southeast Asian J Trop Med Public Health 1999; 30(2):287-295.

(122) Ramoso-Jalbuena J. Climacteric Filipino women: a preliminary survey in the Philippines. Maturitas 1994; 19(3):183-190.

(123) Rekers H, Drogendijk AC, Valkenburg HA, Riphagen F. The menopause, urinary incontinence and other symptoms of the genito-urinary tract. Maturitas 1992; 15(2):101-111.

(124) Bang RA, Bang AT, Baitule M, Choudhary Y, Sarmukaddam S, Tale O. High prevalence of gynaecological diseases in rural Indian women. Lancet 1989; 1(8629):85-88.

(125) Berg G, Gottwall T, Hammar M, Lindgren R, Gottgall T. Climacteric symptoms among women aged 60-62 in Linkoping, Sweden, in 1986. Maturitas 1988; 10(3):193-199.

(126) Garde K, Lunde I. Female sexual behaviour. A study in a random sample of 40-year-old women. Maturitas 1980; 2(3):225-240.

(127) Glatt AE, Zinner SH, McCormack WM. The prevalence of dyspareunia. Obstet Gynecol 1990; 75(3 Pt 1):433-436.

(128) Whorwell PJ, McCallum M, Creed FH, Roberts CT. Non-colonic features of irritable bowel syndrome. Gut 1986; 27(1):37-40.

(129) Iosif CS, Bekassy Z. Prevalence of genito-urinary symptoms in the late menopause. Acta Obstet Gynecol Scand 1984; 63(3):257-260.

(130) Abdo CH, Oliveira WM, Jr., Moreira ED, Jr., Fittipaldi JA. Prevalence of sexual dysfunctions and correlated conditions in a sample of Brazilian women--results of the Brazilian study on sexual behavior (BSSB). Int J Impot Res 2004; 16(2):160-166.

(131) Castelo-Branco C, Blumel JE, Araya H, Riquelme R, Castro G, Haya J et al. Prevalence of sexual dysfunction in a cohort of middle-aged women: Influences of menopause and hormone replacement therapy. Journal of Obstetrics & Gynaecology 2003; 23(4):426-430.

(132) Shokrollahi P, Mirmohamadi M, Mehrabi F, Babaei G. Prevalence of sexual dysfunction in women seeking services at family planning centers in Tehran. Journal of Sex & Marital Therapy 1999; 25(3):211-215.

(133) Rosen RC, Taylor JF, Leiblum SR, Bachmann GA. Prevalence of sexual dysfunction in women: results of a survey study of 329 women in an outpatient gynecological clinic. Journal of Sex & Marital Therapy 1993; 19(3):171-188.

(134) Robinson JC, Plichta S, Weisman CS, Nathanson CA, Ensminger M. Dysmenorrhea and use of oral contraceptives in adolescent women attending a family planning clinic. American Journal of Obstetrics & Gynecology 1992; 166(2):578-583.

(135) Danaci AE, Oruc S, Adiguzel H, Yildirim Y, Aydemir O. Relationship of sexuality with psychological and hormonal features in the menopausal period. West Indian Medical Journal 2003; 52(1):27-30.

(136) Nappi RE, Verde JB, Polatti F, Genazzani AR, Zara C. Self-reported sexual symptoms in women attending menopause clinics. Gynecologic & Obstetric Investigation 2002; 53(3):181-187.

(137) El Defrawi, Dandash KF, Refaat AH, Eyada M. Female genital mutilation and its psychosexual impact. Journal of Sex & Marital Therapy 2001; 27(5):465-473.

(138) Versi E, Harvey MA, Cardozo L, Brincat M, Studd JW. Urogenital prolapse and atrophy at menopause: a prevalence study. International Urogynecology Journal 2001; 12(2):107-110.

(139) Shah PN, Smith JR, Wells C, Barton SE, Kitchen VS, Steer PJ. Menstrual symptoms in women infected by the human immunodeficiency virus. Obstet Gynecol 1994; 83(3):397-400.

(140) Pepe F, Garozzo G, Pepe P. Incidence of sexual dysfunction in healthy sexually active Sicilian women of fertile age spontaneously attending a private gynaecologist for routine examination. Journal of Obstetrics & Gynaecology 1991; 11(4):277-280.

(141) Mahmood TA, Templeton AA, Thomson L, Fraser C. Menstrual symptoms in women with pelvic endometriosis. BJOG 1991; 98(6):558-563.

(142) Bachmann GA, Leiblum SR, Grill J. Brief sexual inquiry in gynecologic practice. Obstet Gynecol 1989; 73(3 I):425-427.

(143) Schein M, Zyzanski SJ, Levine S, Medalie JH, Dickman RL, Alemagno SA. The frequency of sexual problems among family practice patients. Fam Pract Res J 1988; 7(3):122-134.

(144) Warner P. Psychiatric disorder and gynaecological symptoms in middle aged women. Br Med J (Clin Res Ed) 1987; 294(6578):1033-1034.

(145) Heisterberg L. Factors influencing spontaneous abortion, dyspareunia, dysmenorrhea, and pelvic pain. Obstet Gynecol 1993; 81(4):594-597.

(146) Levine SB, Yost MA, Jr. Frequency of sexual dysfunction in a general gynecological clinic: an epidemiological approach. Arch Sex Behav 1976; 5(3):229-238.

(147) Ismael NN. A study on the menopause in Malaysia. Maturitas 1994; 19(3):205-209.

(148) Dudley Chapman J. A longitudinal study of sexuality and gynecologic health in abused women. Journal of American Osteopathic Association 1989; 89(5):619-623.

(149) Plouffe L, Jr. Screening for sexual problems through a simple questionnaire. Am J Obstet Gynecol 1985; 151(2):166-169.

(150) Buddeberg C, Hess D, Merz J. Sexuelle Probleme von Patienten in der Allgemeinpraxis.[Sexual problems of patients in general practice]. Schweiz Rundsch Med Prax 1984; 73(37):1113-1118.

(151) Zondervan KT, Yudkin PL, Vessey MP, Dawes MG, Barlow DH, Kennedy SH. Prevalence and incidence of chronic pelvic pain in primary care: Evidence from a national general practice database. Br J Obstet Gynaecol 1999; 106(11):1149-1155.

(152) Rulin MC, Davidson AR, Philliber SG, Graves WL, Cushman LF. Long-term effect of tubal sterilization on menstrual indices and pelvic pain. Obstet Gynecol 1993; 82(1):118-121.

(153) Thongkrajai P, Pengsaa P, Lulitanond V. An epidemiological survey of female reproductive health status: gynecological complaints and sexually-transmitted diseases. Southeast Asian J Trop Med Public Health 1999; 30(2):287-295.

(154) Filippi V, Marshall T, Bulut A, Graham W, Yolsal N. Asking questions about women's reproductive health: validity and reliability of survey findings from Istanbul. Trop Med Int Health 1997; 2(1):47-56.

(155) Mathias SD, Kuppermann M, Liberman RF, Lipschutz RC, Steege JF. Chronic pelvic pain: prevalence, health-related quality of life, and economic correlates. Obstet Gynecol 1996; 87(3):321-327.

(156) Bhatia JC, Cleland J. Self-reported symptoms of gynecological morbidity and their treatment in south India. Studies in Family Planning 1995; 26(4):203-216.

(157) Kirkengen AL, Schei B, Steine S. Indicators of childhood sexual abuse in gynaecological patients in a general practice. Scand J Prim Health Care 1993; 11(4):276-280.

(158) Bang RA, Bang AT, Baitule M, Choudhary Y, Sarmukaddam S, Tale O. High prevalence of gynaecological diseases in rural Indian women. Lancet 1989; 1(8629):85-88.

(159) Frljak A, Cengic S, Hauser M, Schei B. Gynecological complaints and war traumas. A study from Zenica, Bosnia-Herzegovina during the war. Acta Obstetricia et Gynecologica Scandinavica 1997; 76(4):350-354.

(160) Desai VK, Kosambiya JK, Thakor HG, Umrigar DD, Khandwala BR, Bhuyan KK. Prevalence of sexually transmitted infections and performance of STI syndromes against aetiological diagnosis, in female sex workers of red light area in Surat, India. Sexually Transmitted Infections 2003; 79(2):111-115.

(161) Mahmood TA, Templeton AA, Thomson L, Fraser C. Menstrual symptoms in women with pelvic endometriosis. BJOG 1991; 98(6):558-563.

(162) Heisterberg L, Hebjorn S, Andersen LF, Petersen H. Sequelae of induced first-trimester abortion. A prospective study assessing the role of postabortal pelvic inflammatory disease and prophylactic antibiotics. Am J Obstet Gynecol 1986; 155(1):76-80.

(163) Iglesias R, Terres A, Chavarria A. Disorders of the menstrual cycle in airline stewardesses. Aviat Space Environ Med 1980; 51(5):518-520.
